# Ectopic ATP synthase stimulates the secretion of extracellular vesicles in cancer cells

**DOI:** 10.1038/s42003-023-05008-5

**Published:** 2023-06-15

**Authors:** Yi-Chun Kao, Yi-Wen Chang, Charles P. Lai, Nai-Wen Chang, Chen-Hao Huang, Chien-Sheng Chen, Hsuan-Cheng Huang, Hsueh-Fen Juan

**Affiliations:** 1 Department of Life Science, National Taiwan University, Taipei, 106 Taiwan; 2 Institute of Atomic and Molecular Sciences, Academia Sinica, Taipei, 106 Taiwan; 3 Institute of Molecular and Cellular Biology, National Taiwan University, Taipei, 106 Taiwan; 4 Graduate Institute of Biomedical Electronics and Bioinformatics, National Taiwan University, Taipei, 106 Taiwan; 5 Department of Food Safety / Hygiene and Risk Management, National Cheng Kung University, Tainan, Taiwan; 6 Institute of Biomedical Informatics, National Yang Ming Chiao Tung University, Taipei, 112 Taiwan; 7 Center for Computational and Systems Biology, National Taiwan University, Taipei, 106 Taiwan

**Keywords:** Cancer microenvironment, Cancer

## Abstract

Ectopic ATP synthase on the plasma membrane (eATP synthase) has been found in various cancer types and is a potential target for cancer therapy. However, whether it provides a functional role in tumor progression remains unclear. Here, quantitative proteomics reveals that cancer cells under starvation stress express higher eATP synthase and enhance the production of extracellular vesicles (EVs), which are vital regulators within the tumor microenvironment. Further results show that eATP synthase generates extracellular ATP to stimulate EV secretion by enhancing P2X_7_ receptor–triggered Ca^2+^ influx. Surprisingly, eATP synthase is also located on the surface of tumor-secreted EVs. The EVs-surface eATP synthase increases the uptake of tumor-secreted EVs in Jurkat T-cells via association with Fyn, a plasma membrane protein found in immune cells. The eATP synthase-coated EVs uptake subsequently represses the proliferation and cytokine secretion of Jurkat T-cells. This study clarifies the role of eATP synthase on EV secretion and its influence on immune cells.

## Abstract (web summary)

eATP synthase is expressed on the surface of cancer-derived extracellular vesicles (EVs), contributes to EV uptake and reduces Jurkat T cell proliferation and cytokine release.

## Introduction

FoF1-ATP synthase is a well-known protein complex that has been recognized as a nano-sized machine specifically localized on the inner membrane of mitochondria, with the F1 domain facing the mitochondrial matrix^1–4^. However, recent studies have shown that ATP synthase is also found on the cell surfaces with the F1 domain facing the extracellular space. This subtype of ATP synthase is called ectopic ATP (eATP) synthase, and eATP synthase can be found in many eukaryotic cell lines and most cancer tissues^5–8^. eATP synthase originates in mitochondria and is transported towards the cell surface as a result of mitochondrial fragmentation and cytoskeletal trafficking mediated by Drp1 and KIF5B^9^. Besides, other complexes related to the electron transport chain have also been detected on the cell membranes where they work alongside eATP synthase to produce ATP that is subsequently released into the extracellular space^5^. eATP synthase also regulates intracellular pH, cholesterol homeostasis, and cell proliferation and differentiation owing to its function as a proton channel and its actions as a receptor^10–14^. In cancer cells, eATP synthase is specifically involved in angiogenesis and in the immune recognition of tumor cells^15,16^. Due to these functions, eATP synthase has also been considered as a therapeutic target for the treatment of numerous cancers^17,18^. In multicellular organisms, cell-to-cell communication via the secretion of extracellular vesicles has become an area of research focus over the past decade^19–21^. Extracellular vesicles (EVs) are nano-sized, membrane-bound vesicles composed of a lipid bilayer containing transmembrane proteins, as well as enclosing proteins, lipids, and nucleic acids. Cells release EVs into their environment, whether that be blood, urine, or conditioned culture media, and EVs can affect neighboring cells and/or change the biochemical properties of the extracellular space^22–25^. Different kinds of EV subpopulations have been described, including microvesicles (100–1000 nm), exosomes (40–120 nm), apoptotic bodies (50–2000 nm), and large oncosomes (1–10 μm)^26^. EVs are released either via fusion of multi-vesicular bodies (MVBs) with the plasma membrane or via direct budding from the plasma membrane. Both processes are mediated by endosomal sorting complexes required for transport (ESCRT), tetraspanins, and subclasses of the Rat sarcoma virus (Ras) family of guanosine triphosphatases (GTPases)^27–30^. There are several ways to stimulate EV release. One of the most common factors is the calcium ion (Ca^2+^). Ca^2+^ concentration is a well-known intracellular signal regulating secretion in many cell types. It has been reported that Ca^2+^ participates in affecting the function of synaptosomal-associated protein (SNAP) receptors and protein unc-13 homolog D (Munc13-4)^31–35^. In addition, Rab binding protein is also required for Ca^2+^-dependent membrane fusion^36^. The way in which intracellular Ca^2+^ commonly mediates its actions is through either Ca^2+^ release from the endoplasmic reticulum (ER) or Ca^2+^ influx via transmembrane channels found on the cell plasma membrane — for example, the P2X_7_ receptor, which is an example of an ATP-gated channel^37,38^. The P2X_7_ receptor is highly expressed on cancer cells and requires a high concentration of ATP (at least 100 μM) for its activation^39,40^. While this does not tend to occur in healthy tissue (10–100 nM), tumor tissue micro-environments can easily reach such extracellular concentrations of ATP (100–500 μM)^41–44^. Additionally, EVs play an essential role in cancer. Many studies have indicated that EVs derived from tumor cells possess the ability to promote neighboring non-tumor cells to become tumor growths^45–48^. Furthermore, EVs can contribute to tumor growth and progression via several mechanisms, such as the promotion of angiogenesis and metastasis^49–51^. EVs can also protect tumor cells from antitumor drugs by exchanging drug transporters between cells, thereby contributing to drug resistance^52–54^. In addition, EVs-mediated immunosuppression plays a critical role for evading immune surveillance. Tumor derived EVs delivered cytokines and RNAs to regulate immune cell population and decrease the signaling of antitumor responses^55,56^. Understanding the mechanisms related to EVs will be beneficial for clinical therapy. In recent studies, EVs have already been targeted in commercial cancer tests^57,58^. Therefore, EVs have become a crucial area of study in cancer research^59,60^. In this study, cancer-related starvation stress induced the expression of eATP synthase and stimulated the release of EVs by providing extracellular ATP to the P2X_7_ receptor. EVs play a critical role in cancer progression through various ways as described above, so the regulation of EVs by eATP synthase is important for cancer cells under stress. Besides, eATP synthase was also identified in EVs derived from cancer cells, contributing to immunosuppression by interacting with Fyn, as shown in Jurkat T-cells. This illustrates that eATP synthase not only increased EV secretion, but also benefitted the tumor via EV communication. Therefore, an investigation into the mechanisms of eATP synthase in EV release and uptake is necessary for the comprehension of a regulator of cancer cell communication and the provision of a therapeutic target against tumor development.

## Results

### Serum starvation induces eATP synthase expression and release of EVs

**Fig. 1:**
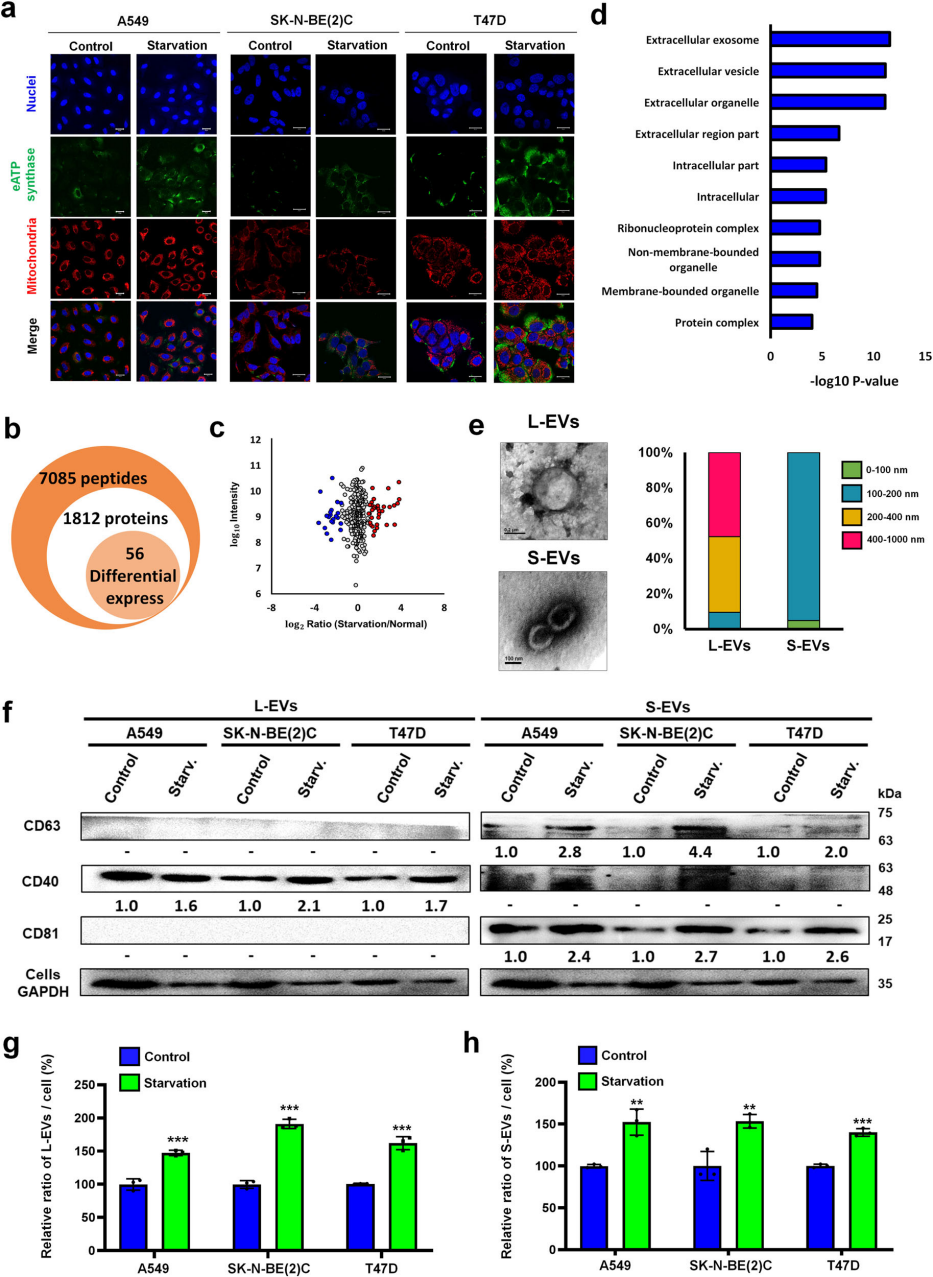
Quantitative proteomics shows that serum deprivation alters vesicle transport. **a** A549, SK-N-BE(2)C and T47D cells were treated with 0.1% FBS (starvation conditions) or 10% FBS (control conditions) and then probed with an anti-ATP synthase complex antibody (green) and MitoTracker (red). Scale bar, 20 μm. **b** The number of peptides, proteins, and differentially expressed proteins with a fold change >1.96 standard deviation and *p*-value < 0.05 revealed by proteomics. **c** Scatter plot showing the log_2_ of dimethyl-labeled ratios against the log_10_ intensity of each protein. Red and blue indicate significantly upregulated and downregulated proteins, respectively. **d** Horizontal bar chart of GO term enrichment (cell components). **e** Different types (L- and S-EVs) of EV from cell-conditioned media were isolated by serial ultracentrifugation and characterized using a transmission electron microscope. The size distribution were analyzed by NTA. **f** L- and S-EVs were isolated from the media of A549, SK-N-BE(2)C and T47D cells and performed by western blot. EV-specific markers were used as probes, and their intensities were quantified using ImageJ. **g**, **h** L- and S-EVs derived from A549, SK-N-BE(2)C, and T47D cells were quantified by NTA. The values represent the mean ± SD (*n* = 3).

Recent studies have shown that eATP synthase is expressed on cancer cells^5^. We further found that the expression of eATP synthase on A549, SK-N-BE(2)C and T47D cells was induced by serum starvation with medium containing 0.1% FBS (Fig. 1a). In this study, the cells were fixed and subsequently probed with an anti-ATP synthase antibody, without the use of any detergent to penetrate the cell membrane. This suggests that the signal detected from the probed ATP synthase (green) originated from the outside of the cell surface. To confirm that the probed ATP synthase was not present in the mitochondria, we stained the mitochondria (red) and observed that the two signals did not overlap. Additionally, our previous study revealed that eATP synthase was transported from the mitochondria to the plasma membrane via Drp1 and KIF5B carried along microtubules^9^, so we were curious whether starvation-induced expression of eATP synthase was also caused by the same mechanism. To elucidate the influence of serum starvation on the high-level expression of eATP synthase on cell surfaces, A549 cells were treated with 0.1% FBS medium (starvation) or 10% FBS medium, and were further analyzed by quantitative proteomics (Supplemental Fig. 1a). A total of 7085 peptides, belonging to 1812 proteins, were identified. In all, 305 quantified proteins (with H/L ratio, Supplementary Data 1) of them were further identified fold change >1.96 standard deviation and *p*-value < 0.05 as cut-offs to filter out the major differentially-expressed proteins after starvation (Fig. 1b, c). In addition to the above, the 56 differentially expressed proteins (Supplementary Data 1. Red, up-regulated. Blue, down-regulated) were analyzed via Gene Ontology (GO) analysis using the DAVID web tool. According to the GO analysis of biological processes, the term ‘mitochondrion transport along microtubules’, strongly corroborated with the pathway of eATP synthase biogenesis that, according to research, revealed that the increasing eATP synthase may have originated from the mitochondria during starvation (Supplemental Fig. 1b)^9^. On the other hand, the functional terms ‘extracellular exosome’ and ‘extracellular vesicle’ were strikingly enriched with the lowest p-value (Fig. 1d and Supplementary Data 2). The finding implied that serum starvation may influence EV release. We validated the proteomics results, using the lung cancer cell line A549, neuroblastoma cell line SK-N-BE(2)C and breast cancer cell line T47D, to see whether there were any generalized phenomena across cancer cells. To further confirm whether EV release is influenced by starvation, we first isolated EVs from the cell culture medium using differential ultra-centrifugation (Supplemental Fig. 2a), and the large EVs and small EVs (L- and S-EVs) were characterized by transmission electron microscopy and nanoparticle tracking analysis (NTA) (Fig. 1e and Supplemental Fig. 2b, c). After characterization, the L- and S-EVs derived from A549, SK-N-BE(2)C and T47D cells were isolated, and their quantities were compared by western blots using CD40 as the L-EV–specific marker and CD63 and CD81 as S-EV–specific markers^26^. We compared EV concentrations between groups by loading equal volumes of extract samples from the medium. Furthermore, we collected EVs and their cell lysates simultaneously under various conditions and used the cell lysates as a standard to normalize the EV concentration (Fig. 1f). Both results of L- and S-EVs were validated by NTA (Fig. 1g, h). The results showed that secretion of EVs was indeed increased under serum starvation, suggesting that eATP synthase likely plays a role in enhancing EV release.

### Interrupted biogenesis of eATP synthase decreases EV secretion

**Fig. 2:**
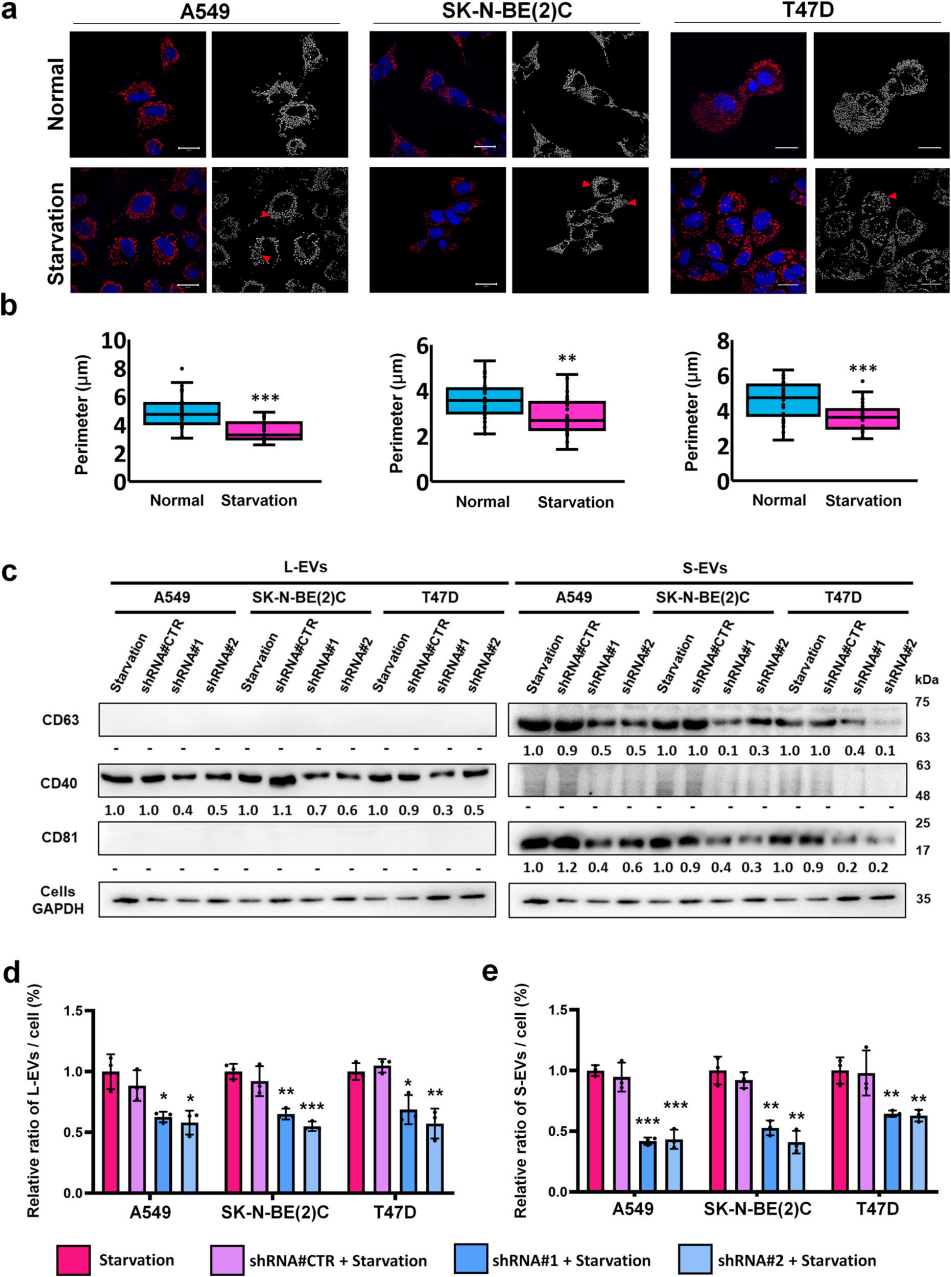
Mitochondrial fission stimulated by eATP synthase expression influenced extracellular vesicle release. **a** A549, SK-N-BE(2)C, and T47D cells were treated with either 0.1% FBS (starvation conditions) or 10% FBS (normal conditions) and then probed with MitoTracker, which detected fission using confocal microscopy. Scale bar, 20 μm. **b** The images in A were analyzed by Icy software. The values represent the mean ± SD (*n* = 30). **c**–**e** EVs derived from cancer cells or Drp1-knockdown cancer cells (shRNA#1, shRNA#2, and shRNA#CTR as a vector control) under starvation treatment were quantified using NTA and western blot. EV-specific markers were used as probes, and their intensities were quantified using ImageJ. The values represent the mean ± SD (*n* = 3).

In our recent work on cancer cells, we found that mitochondrial fragmentation promoted eATP synthase formation. A protein specific to mitochondrial fission, Drp1 (Dynamin-1-like protein), is involved in the trafficking of eATP synthase from the mitochondria to the cell surface along microtubules and increases the expression of eATP synthase^9^. Based on the DAVID analysis, mitochondria transport along microtubules enriched after starvation treatment, implying that the starvation-enhanced expression of eATP synthase may also originate from mitochondria fission (Supplemental Fig. 1b). Therefore, to confirm whether the serum starvation treatment promotes mitochondrial fission, we first analyzed the mitochondrial perimeter in A549, SK-N-BE(2)C, and T47D cells under starvation and normal conditions using Icy software (Supplemental Fig. 3). The mitochondria tended to undergo fission after serum starvation in A549, SK-N-BE(2)C, and T47D cells (Fig. 2a, b). To better understand whether EV release was influenced by eATP synthase, we decided to interfere with the generation of eATP synthase and check whether this affected EV release. We used mdivi-1, a Drp1 inhibitor, to inhibit mitochondrial fission^61,62^. After treatment of with 30 µM mdivi-1, the level of eATP synthase decreased (Supplemental Fig. 4a). We further treated A549, SK-N-BE(2)C, and T47D cells with 30 µM of mdivi-1 for 24 h under starvation before collecting and quantifying the EVs released under each condition. After treatment with mdivi-1, the statistics derived from the NTA showed that both L-EV and S-EV release were significantly decreased (Supplemental Fig. 4b, c). The results were also confirmed by immunoblots (Supplemental Fig. 4d). Furthermore, to check that EV release was reduced by mdivi-1 via the inhibition of Drp1, we directly knocked down the Drp1 protein in A549, SK-N-BE(2)C and T47D cells. The secretion of EVs was also decreased in A549, SK-N-BE(2)C and T47D cells after Drp1 knockdown (Fig. 2c–e). These results suggest that starvation induced mitochondria fission, which caused an increased expression of eATP synthase. Decreasing mitochondria fission reduced EV release, indicating that eATP synthase may play a vital role in EV release.

### eATP synthase supplies ATP to stimulate EV secretion

**Fig. 3:**
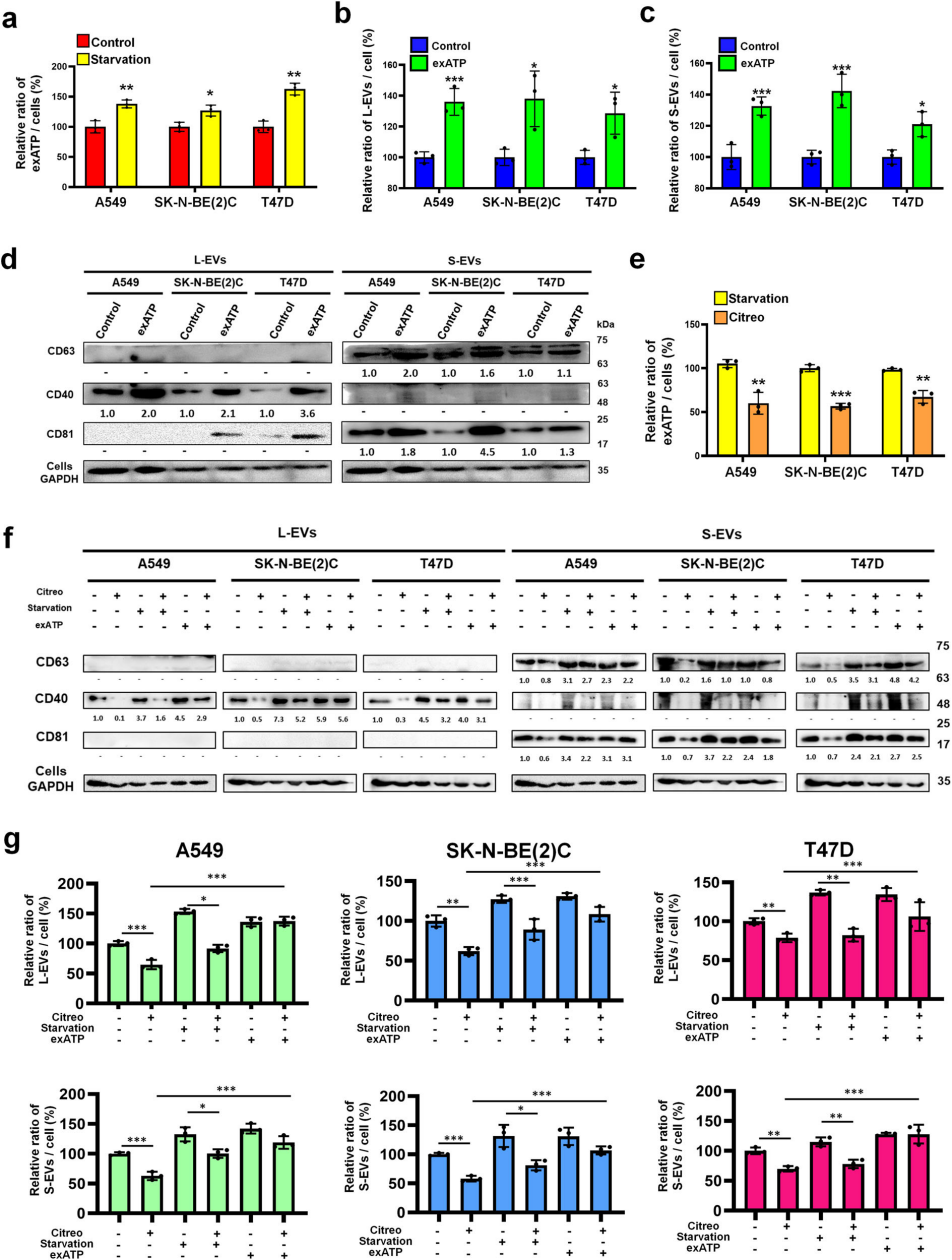
Extracellular ATP influences extracellular vesicle secretion. **a** Extracellular ATP concentrations around A549, SK-N-BE(2)C, and T47D cells were determined after starvation treatment for 18 h. **b**–**d** A549, SK-N-BE(2)C, and T47D cells were treated with 200 μM ATP (exATP) or DMSO (control) for 6 h, and EVs were isolated from media and detected using NTA and western blots. **e** Extracellular ATP concentrations from A549, SK-N-BE(2)C, and T47D cells were determined after treatment with citreoviridin under starvation (Citreo) or starvation for 24 h. **f**, **g** A549, SK-N-BE(2)C and T47D cells were treated with 2 μM citreoviridin (Citreo), DMSO, starvation or 200 μM ATP (exATP), and EVs were isolated from media and detected using NTA and western blot. The values represent the mean ± SD (*n* = 3).

Increasing (starvation) or decreasing (Drp1 knock-down) eATP synthase expression indeed influences EV secretion, but the mechanism is still unknown. According to recent research, eATP synthase can produce ATP in the extracellular space or serve as a receptor for several ligands^5,11^. We first compared the concentration of extracellular ATP after starvation treatment to confirm whether starvation induces an increase in extracellular ATP levels. Our results confirmed this hypothesis, showing that extracellular ATP levels are higher after the starvation treatment (Fig. 3a). The concentrations of extracellular ATP were increased at a 1.38-, 1.26- and 1.63-fold change for A549, SK-N-BE(2)C, T47D cells, respectively, after the starvation treatment. We then incubated A549, SK-N-BE(2)C and T47D cells with 200 μM ATP, which is a concentration similar to that observed under starvation conditions, for 6 h, and collected EVs from the media. The results revealed that the secretion of EVs in SK-N-BE(2)C, A549 and T47D cells was increased after extracellular ATP incubation (Fig. 3b–d). Although the experiments demonstrated that starvation enhanced the level of extracellular ATP and induced the release of EVs, we further confirmed whether the extracellular ATP that stimulated EV secretion was generated by eATP synthase. To block eATP synthase, we used citreoviridin, an eATP synthase inhibitor identified in previous studies, to inhibit extracellular ATP produced by the eATP synthase present on cell surfaces^17,18^. The release of extracellular ATP after citreoviridin treatment was lower than that of the control (Fig. 3e), indicating that citreoviridin inhibited the function of eATP synthase on the cell surface. Next, the EVs were isolated after citreoviridin treatment and quantified by NTA and western blot.

Under starvation treatment, the release of EVs was reduced when eATP synthase was blocked. Intriguingly, when citreoviridin was used to block eATP synthase, the release of EVs was rescued by the exogenously applied ATP (as shown in Fig. 3f, g). From these results, we propose that the secretion of EVs is stimulated by extracellularly accumulated ATP generated by eATP synthase after starvation treatment.

### P2X_7_ receptors utilize extracellular ATP for EV release

**Fig. 4:**
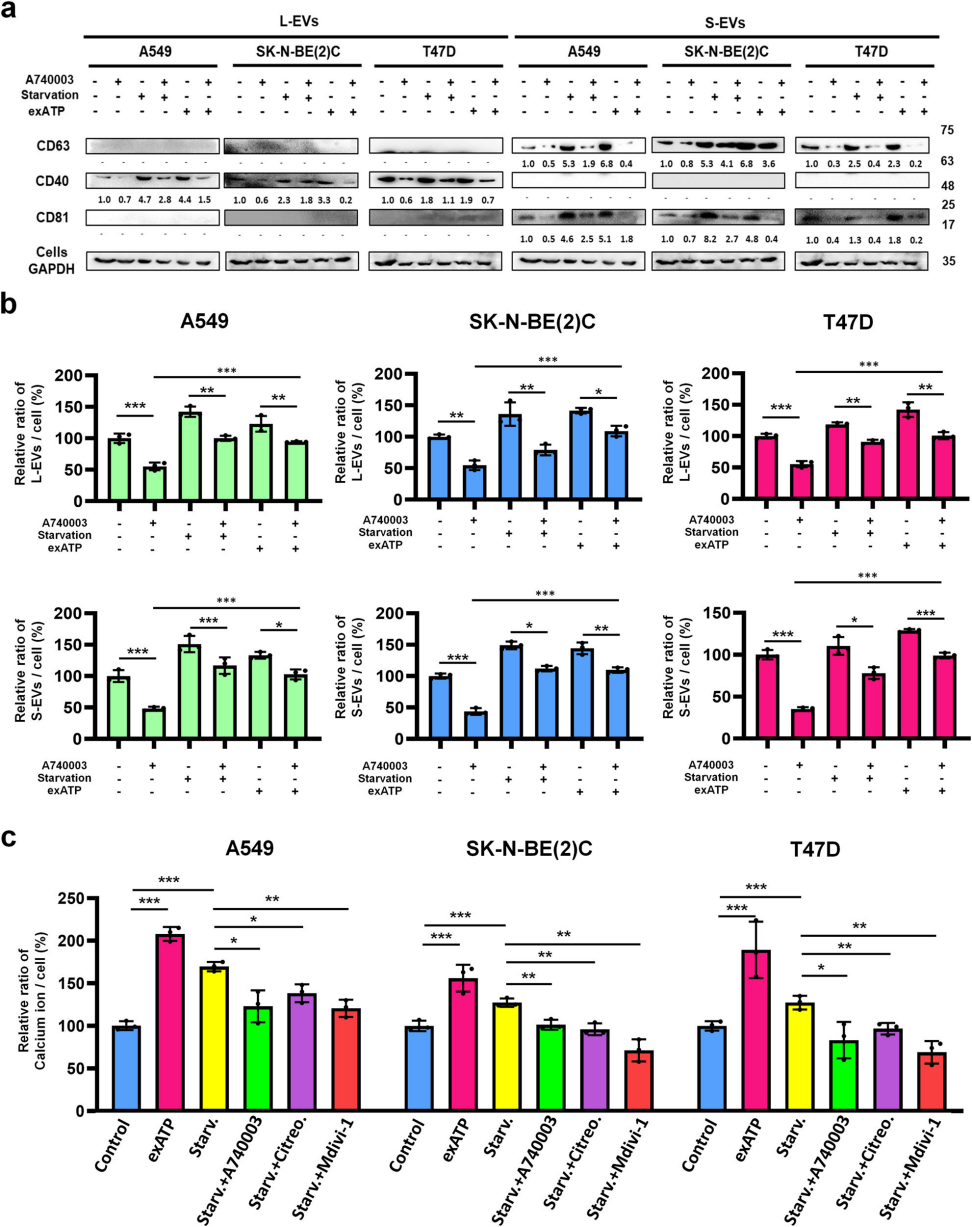
The effect of P2X_7_ receptors on extracellular vesicle secretion is stimulated by eATP synthase. **a**, **b** A549, SK-N-BE(2)C, and T47D cells were treated with 0.1% FBS (starvation conditions), 10% FBS (control conditions) or 20 μM A740003 for 24 h. Subsequently, 200 μM ATP was added, and cells were incubated for 6 h. EVs were isolated from media and detected using NTA and western blot. **c** A549, SK-N-BE(2)C and T47D cells were treated with DMSO (control), 0.1% FBS (starv.) for 18 h, 200 μM ATP (exATP) for 6 h, 2 μM citreoviridin (Citreo) for 24 h, 30 μM mdivi-1 for 24 h, or 20 μM A740003 for 24 h. The levels of Ca^2+^ in the cell lysates were determined according to the user manual of the calcium ion assay kit. The values represent the mean ± SD (*n* = 3).

To understand how extracellular ATP induces the release of EVs, we surveyed research related to the release of EVs, and identified the P2X_7_ receptor as a probable candidate. P2X_7_ is an ATP-gated cation channel that plays a role in exocytosis by increasing intracellular Ca^2+^ concentration. The increasing intracellular Ca^2+^ causes exocytosis of multi-vesicular bodies (MVB), which allows the derivation of small EVs. Moreover, previous studies have revealed the possibility of P2X_7_-induced large EV shedding^63,64^. We considered the possibility that the accumulated extracellular ATP supplied by eATP synthase may stimulate P2X_7_ receptors and lead to EV secretion. First, we blocked P2X_7_ receptors on A549, SK-N-BE(2)C and T47D cell surfaces with A740003, a P2X_7_ inhibitor. The release of EVs was decreased in A549, SK-N-BE(2)C and T47D cells (Fig. 4a, b), similar to what has been reported by other research^34,37^. Intriguingly, A740003 inhibition before starvation or extracellular ATP incubation weakened the stimulus for EV secretion (Fig. 4a, b). The results demonstrated that the secretion of EVs was induced by ATP production from increasing eATP synthase after starvation treatment, and was inhibited by P2X_7_ receptor blockade. We elucidated that the higher levels of eATP synthase under starvation conditions supplied large amounts of ATP to the extracellular space and stimulated the P2X_7_ receptor to influence the release of EVs. To further confirm the influence of eATP synthase on P2X_7_, we quantified cytosolic Ca^2+^ concentrations under several conditions, including starvation, P2X_7_ inhibition, Drp1 inhibition, eATP synthase inhibition, and extracellular ATP incubation. We found that starvation and extracellular ATP incubation resulted in increased intracellular Ca^2+^ concentrations, and this was supported by the fact that extracellular ATP stimulated P2X_7_ receptors to uptake Ca^2+^ (Fig. 4c). Furthermore, Drp1 inhibition, eATP synthase inhibition, or P2X_7_ inhibition downregulated Ca^2+^ uptake under starvation treatment, suggesting that eATP synthase plays a critical role in stimulating P2X_7_ receptors to take up Ca^2+^.

### S-EVs carried more eATP synthase on the surface during starvation

**Fig. 5:**
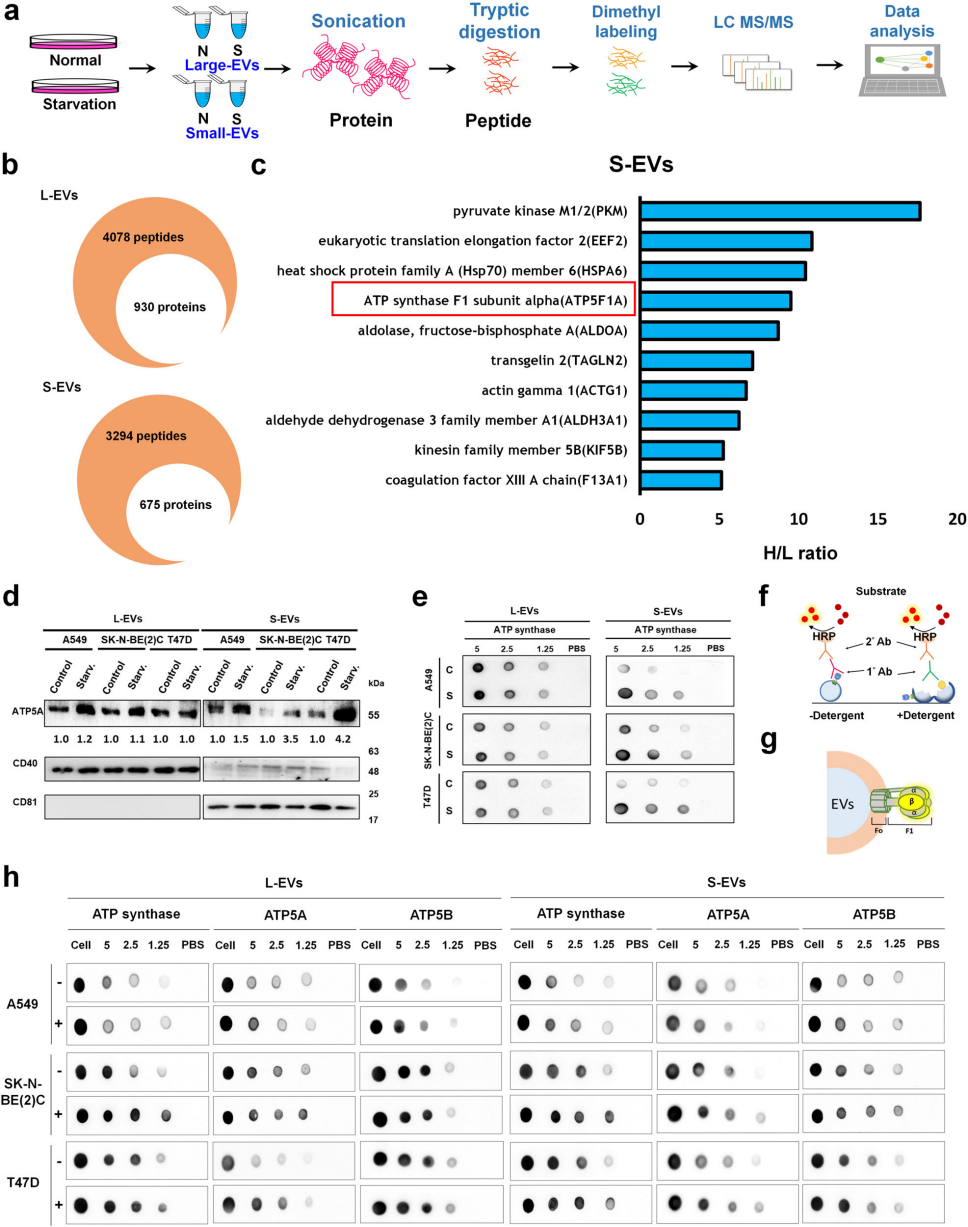
Proteomics and immunoblot demonstrated that ATP synthase was secreted to S-EVs and localized on surface of S-EVs. **a** Schematic overview of the methodology used in this experiment. **b** The numbers of peptides and proteins revealed by the proteomics analysis of L- and S-EVs. **c** Horizontal bar charts of top-10 up-regulated protein with *P*-value < 0.05 in S-EVs. **d** A549, SK-N-BE(2)C and T47D cells were treated with 0.1% FBS (starv.) or 10% FBS (control). The EV samples were collected and performed by western blot. **e** A549, SK-N-BE(2)C and T47D cells were treated with 0.1% FBS (S) or 10% FBS (C). The EV samples were collected and performed by dot blot. **f** Schematic of dot blot experiments. **g** The model of ATP synthase localized on the EV membrane, with the F1 domain facing out. **h** L- and S-EVs derived from A549, SK-N-BE(2)C and T47D cells were serially diluted and dotted on the NC membrane, incubated with or without detergent, and probed using a 1:1000 dilution of anti-ATP synthase complex, anti-ATP5A or anti-ATP5B antibody. +, detergent lysis (PBST); –, no detergent added.

After elucidating the mechanism of EV secretion under serum starvation, we next wondered about its function. First, we performed quantitative proteomics analysis of L- and S-EVs derived from 0.1% FBS DMEM- (starvation) or 10% FBS DMEM-treated A549 cells (Fig. 5a). The 930 and 675 proteins were identified in L- and S-EVs respectively (Fig. 5b). Among the 123 and 84 quantified proteins (with H/L ratio), we observed a significant upregulation of the ATP synthase subunit, ATP5A, in S-EVs, with a top-4 fold change, while no significant change was observed in L-EVs (Fig. 5c and Supplementary Data 3, 4). To further verify the proteome data, we confirmed that expression of ATP5A was increased in S-EVs after starvation treatment, but not in L-EVs (Fig. 5d). We also conducted dot blot experiments with anti-ATP synthase complex antibody to confirm that the expression of eATP synthase was carried along with the S-EVs and increased after starvation treatment (Fig. 5e). Based on these data, we were curious about the location of eATP synthase in S-EVs. We provided dot blot experiments with phosphate-buffered saline (PBS) or PBST (PBS containing 0.05% Tween-20) incubation. Incubation of L- and S-EVs with detergent made the membrane of EVs permeable, allowing antibodies to detect proteins in EVs and not just proteins on the surface (Fig. 5f). We used an anti-ATP synthase complex antibody to confirm the ATP synthase was on the L- or S-EV membrane, and used anti-ATP5B antibody and ATP5A antibody (both are one of the members of the F1 domain) to confirm the orientation of the F1 domain (Fig. 5g). We found that the signal from the ATP synthase complex could be detected without membrane penetration, demonstrating that eATP synthase is present on the both L- and S-EV membrane. ATP5B was also detected under the same conditions, suggesting that the F1 domain of eATP synthase was oriented outward on the L- and S-EV membrane (Fig. 5h).

### eATP synthase may interact with Fyn on the T cell surface

**Fig. 6:**
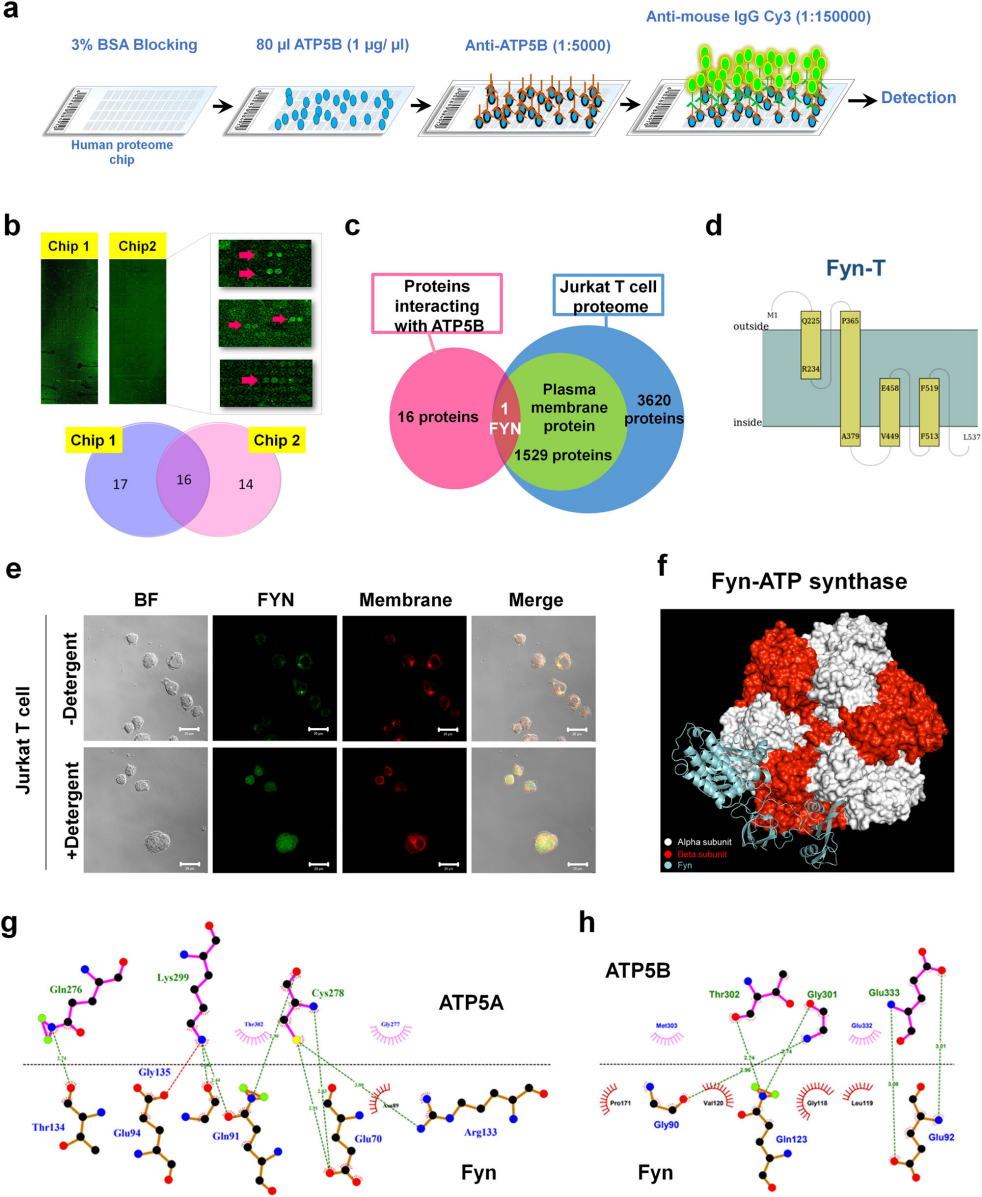
Fyn-T interacted with ATP synthase. **a** Schematic overview of the hybridization of ATP5B with the human proteome microarray. **b** Replicate experiments of the human proteome microarray of ATP5B-interacting proteins. The Venn diagram indicates the overlap among the candidates of the human proteome microarray. **c** Proteomic data of the membrane proteins of Jurkat T cells were obtained (Wu et al.^77^) and compared with our proteome microarray data. **d** The prediction transmembrane protein structure of Fyn-T was analyzed using Membrain. **e** The plasma membranes of Jurkat T cells were stained by Cell-Mask and probed with the anti-Fyn antibody. Scale bar, 20 μm. BF bright field. **f** The docking analysis was performed using ClusPro 2.0 and was presented using PyMOL. Human ATP synthase and Fyn-T were modeled using SWISS-MODEL. **g**, **h** The best binding models of ATP5A (**g**) and ATP5B (**h**) with Fyn-T obtained from docking were analyzed by LigPlot+ 2.2.5 to display the interaction diagrams (partial). The structure of ATP5A and ATP5B are presented on the black line, and Fyn is shown below the black line. Green dotted lines show hydrogen bond interactions. Red and pink semicircles indicate the hydrophobic interactions.

EVs are known to contribute to intercellular communication; ergo, eATP synthase on EVs may also play a role in communication. A function in protein interaction has been previously reported for outward eATP synthase^65^. We first constructed and produced human ATP5B proteins and identified ATP5B-interacting proteins using human proteomic microarrays (Fig. 6a). We then obtained 16 overlapping proteins from two independent biological replicates (Fig. 6b and Supplemental Fig. 5a). We believed that proteins localized to the plasma membrane had a higher chance of interacting with the eATP synthase on S-EVs. One of protein, Fyn, is usually expressed on the plasma membrane, and is associated with neural or immune cells. It has been reported that cancer cell-derived EVs suppress immune cells^66,67^. Therefore, immune cells can be suitable subjects for further experiments. We overlaid the 16 overlapped candidates from the proteomic microarray data with the reported proteome from Jurkat T cells, an immortal immune cell line, and identified Fyn as the only candidate protein (Fig. 6c)^68^. Although Fyn is reported to be localized on the inside of the plasma membrane, we performed a transmembrane prediction analysis of the Fyn-T sequence (the Fyn isoform that is highly expressed in T cells), and found that parts of Fyn are indeed oriented outward (Fig. 6d, Supplementary Data 5)^69–72^. Further, we confirmed this prediction by successfully detecting Fyn on the surface of Jurkat T cells using an anti-Fyn antibody (Fig. 6e). Proteome array data suggests in vitro interaction only between Fyn and ATP5B. To further investigate the interaction between Fyn and whole ATP synthase, we first modeled human ATP synthase with SWISS-MODEL^73^. Because of the high conservation in sequence alignment, we used resolved bovine ATP synthase structure as the template (PDB ID: 2W6J) for human ATP synthase modeling. The sequence of the Fyn-T structure was obtained from UniProt (P06241) and modeled using SWISS-MODEL. Human ATP synthase was further docked using ClusPro^74^ to Fyn-T, with the settings predicting outside residues (1-224, 235-364 amino acids of Fyn-T) as the attraction region (Fig. 6f and Supplementary Data 5). The results showed that the lowest ClusPro score of the ATP synthase-Fyn interaction was -4384.3. As a control, we also docked mitochondria processing peptidase subunit alpha (PMPCA, -988.7), a known ATP synthase interacting protein, and Fanconi anemia group E protein (FANCE, -732.5), a non-ATP synthase interacting protein, to ATP synthase (Supplemental Fig. 5b). Both proteins were composed of about 500 amino acids that were similar to Fyn. Our proteome array data showed that PMPCA interacts with multiple ATP synthase subunits as previously reported^75^, while FANCE has not been reported as an ATP synthase interacting protein and did not interact with ATP5B. Furthermore, we directly analyzed the interaction between the alpha and beta subunits of ATP synthase and the predicted outside sequence of Fyn-T. Numerous interactions, such as hydrogen bonds and hydrophobic contact, were predicted and visualized in Fig. 6g, h (partial data, with the remainder of the data shown in Supplemental Fig. 6a, b). These results illustrated that eATP synthase interacted with Fyn protein, therefore implying that Fyn located on the surface of Jurkat T cells may be a target of eATP synthase on S-EVs from cancer cells.

### Blockade of eATP synthase or Fyn affected Jurkat T cell proliferation, cytokine release and EV uptake

Fyn is a kinase related to multiple cell signaling, including T cell activation, development, and proliferation^76,77^. Our objective was to confirm whether EVs derived from starving cancer cells influenced Jurkat T cell proliferation via the altered EV components, rather than only by increasing EVs. We first collected L- and S-EVs derived from A549, SK-N-BE(2)C, and T47D cells under normal, starvation, and citreoviridin pre-incubated conditions, and washed the cells twice with PBS. To normalize the quantities of EVs from each group, EVs was quantified based on EV-protein levels using the BCA assay^78^, and 20 µg of L- and S-EVs from each group was incubated with Jurkat T cells for two days (Supplemental Fig. 7a, b). The results indicated that S-EVs significantly enhanced their ability to inhibit the growth of Jurkat T cells following starvation treatment, while L-EVs did not demonstrate a significant increase in their capacity to inhibit immune cell growth after starvation treatment (Supplemental Fig. 7c). This observation may reflect that the expression level of ATP synthase in L-EVs did not increase after starvation treatment (Fig. 5d, e). Conversely, the inhibitory potential of S-EVs was restored by citreoviridin treatment after starvation (Supplemental Fig. 7c). This finding confirms that the effect of S-EV is enhanced by starvation and attenuated by blocking eATP synthase on S-EVs. The results also demonstrated that eATP synthase on the S-EV surface may play an important role in influencing immune cells.

**Fig. 7:**
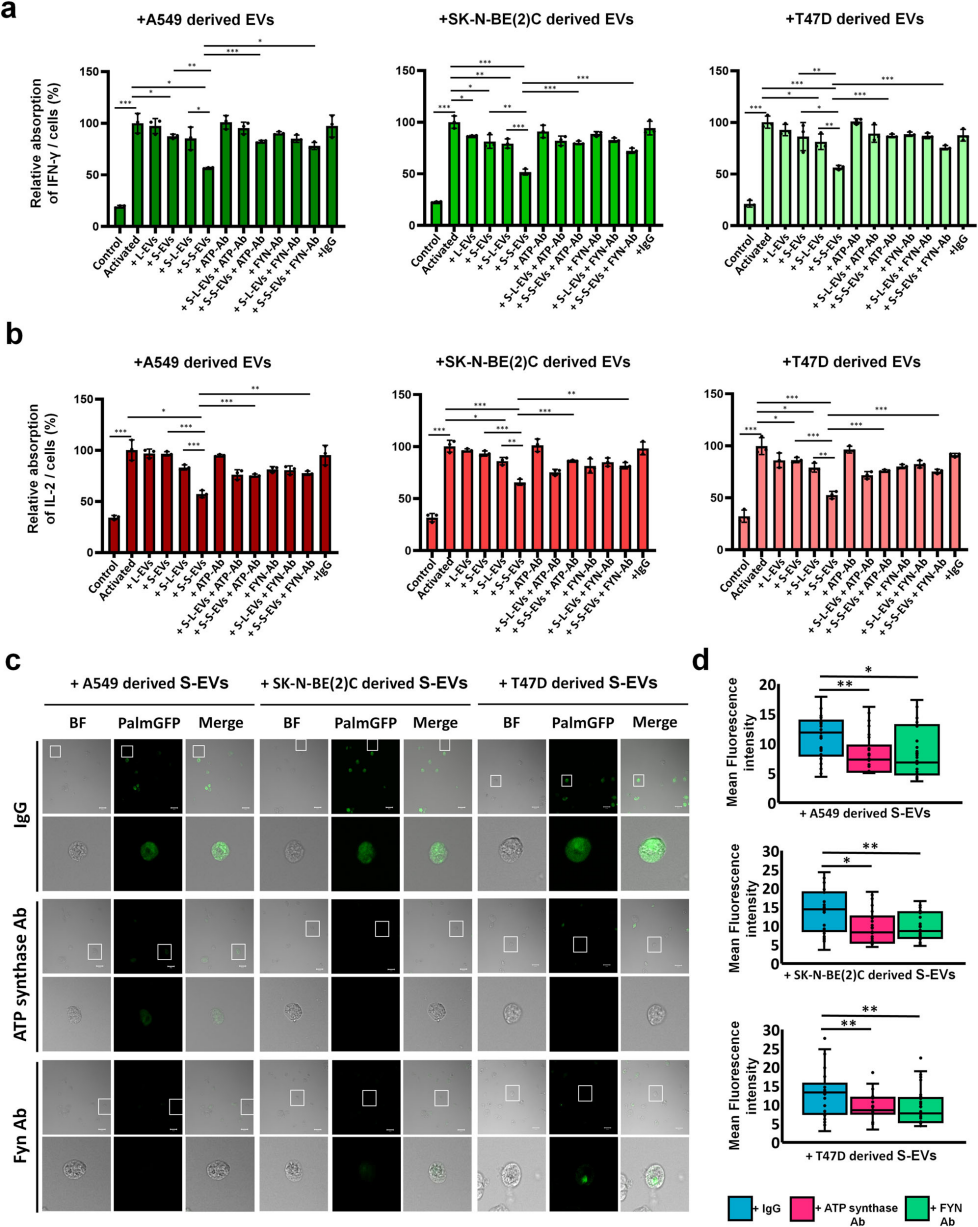
Blockade of ATP synthase on cancer-derived S-EVs or Fyn on Jurkat T cell-inhibited immune response of Jurkat T cells. **a**, **b** In ATP Ab group, the L- or S-EVs derived from A549, SK-N-BE(2)C, and T47D were incubated with the anti-ATP synthase complex antibody, and then added to activated Jurkat T cells. After incubation, Jurkat T cells were performed to IFN-γ and IL-2 ELISA assay to assess cytokine level. In Fyn Ab group, activated Jurkat T cells (1 × 10^4^) were incubated with the anti-Fyn antibody, and then incubated with L- or S-EVs derived from A549, SK-N-BE(2)C, and T47D. After incubation, Jurkat T cells were performed to IFN-γ and IL-2 ELISA assay to assess cytokine level. The cytokine level was normalized by cell number. Activated, PMA and ionomycin activation. S-L-VEs, starvation L-EVs. S-S-VEs, starvation S-EVs. ATP Ab, anti-ATP synthase complex antibody. Fyn Ab anti-Fyn antibody. The values represent the mean ± SD (*n* = 3). **c** The S-EVs (20 μg) derived from palmGFP-labeled A549, SK-N-BE(2)C, and T47D were incubated with the anti-ATP synthase complex antibody or IgG isotype antibodies. The Jurkat T cells were incubated with the anti-Fyn antibody or IgG isotype antibodies. After incubation, S-EVs were incubated with Jurkat T cells then imaged by confocal microscopy. Scale bar, 20 = μm. **d** The intensity of the images was measured using ZEN2009 software. The values represent the mean ± SD (*n* = 30).

To further confirm, targeted blockade of eATP synthase on L-EVs and S-EVs was achieved by pre-incubation with anti-ATP synthase antibody. Antibody incubation not only inhibited the decreasing survival of Jurkat T cells caused by S-EVs, but also rescued the reducing cytokine secretion (INF-γ and IL-2) of Jurkat T cells (Supplemental Fig. 7d and Fig. 7a, b). However, because eATP synthase on L-EVs did not increase with starvation, it exhibited less immune cell-inhibiting ability than S-EVs in previous experiments (Supplemental Fig. 7c). The effect of antibody blockade on L-EVs was not significant in this experiment. Following the study, we conversely blocked Fyn on the Jurkat T cell surface using an anti-Fyn antibody and incubated the cells with L- and S-EVs. Interestingly, inhibiting the proliferation and cytokine release of Jurkat T cells by S-EVs were also rescued via anti-Fyn antibody incubation (Supplemental Fig. 7d and Fig. 7a, b). This finding suggests that the interaction between eATP synthase on S-EVs and Fyn on Jurkat T cells decreased the proliferation and cytokine secretion of Jurkat T cells. We further conducted another experiment to demonstrate that eATP synthase affects the uptake of S-EVs by Jurkat T cells. To track the uptake of S-EVs, we used palmGFP-transfected A549, SK-N-BE(2)C and T47D cells, which were cell lines labeled with enhanced green fluorescence protein (EGFP) at the NH2-termini with a palmitoylation signal on the plasma membrane. The EGFP-labeled membrane will be taken up by EVs, rendering them trackable (Lai et al.^95^). We isolated palmGFP-labeled S-EVs from these cells and incubated them with either an anti-ATP synthase complex antibody or IgG before exposing them to Jurkat T cells. On the other hand, we blocked Fyn on the surface of Jurkat T cells using an anti-Fyn antibody and incubated the cells with labeled S-EVs. Confocal microscopy revealed that Jurkat T cells took up S-EVs, and the EGFP fluorescence signal showed that eATP synthase or Fyn blockade decreased the uptake of S-EVs by the cells (Fig. 7c). The mean fluorescence intensity of each cell was analyzed using statistics (Fig. 7d), and the results showed that eATP synthase on the surface of S-EVs and Fyn on the plasma membrane of Jurkat T cells played a critical role in cell-to-cell communication, indicating that Fyn could influence EV uptake by targeting eATP synthase on S-EVs. This again demonstrated that the eATP synthase contributed on communication between cancer and immune cells via S-EVs.

## Discussion

The mitochondrion is a dynamic organelle, and its continuous dynamics are controlled as a balance between fusion and fission^79,80^. It has been proven that eATP synthase originates from DRP1-dependent mitochondrial fission^9^. Mitochondrial dynamics are also influenced by stress, such as starvation. Prolonged stress induces mitochondrial fission^80^. Our experiments confirmed that the mitochondria of A549, SK-N-BE(2)C, and T47D cells tend to undergo fission after serum starvation, so we considered the possibility that starvation-enhanced expression of eATP synthase is induced by mitochondrial fission. By combining our proteomics data and functional enrichment analyses using DAVID, we found several enriched terms to be related to the mitochondria, particularly mitochondrion transport along microtubules (Supplemental Fig. 1b). A recent proteomic study showed that ATP synthase in the mitochondria is transported along microtubules via DRP1-KIF5B interaction with the cell surface^9^, which is consistent with our present study.

After confirming an increase in eATP synthase expression by starvation, we further investigated the influence of eATP synthase on EV secretion. In the last two decades, eATP synthase has been the focus of much research. Most studies of eATP synthase are related to cancer cell proliferation and survival, or its binding to high density lipoprotein (HDL). The eATP synthase on the cell surface mostly functions to stimulate ATP-gated transmembrane protein complexes, such as the P2X or P2Y family^65,81^. In our research, we found a new mechanism influenced by eATP synthase that is also based on stimulating an ATP-gated transmembrane protein complex. We used several methods to confirm the relationship between eATP synthase and EV release. The cancer microenvironment often contains ATP at a high concentration, especially in when under stress, such as in hypoxia^82,83^. In our study, we validated that starvation-induced eATP synthase expression occurs because of mitochondrial fission. Other stresses have also been found to cause mitochondrial fission^80^. It is possible that increased eATP synthase expression due to mitochondrial fission is one of the reasons why cancer microenvironments have elevated levels of extracellular ATP. Our data shows that ATP is still present in the extracellular space, even when eATP synthase is blocked (Fig. 3e).

Extracellular ATP is one of the major biochemical ingredients of the tumor microenvironment and plays a critical role in immunosuppression^42^. The level of extracellular ATP can be increased in several ways, such as transport through transmembrane channels, exocytosis, and delivery via EVs^44^. Our data confirmed another mechanism through which eATP synthase contributes to the formation of extracellular ATP. We also found that increasing extracellular ATP stimulated EV secretion and promoted immunosuppression in Jurkat T cells, which is another role for eATP synthase and extracellular ATP. Moreover, stress in the tumor microenvironment can lead to angiogenesis, tumorigenesis, or metastasis^84,85^. All these processes involve EV participation; therefore, EV communication is necessary for tumor progression. Recently, it has been reported that EV secretion is associated with the stimulation of the P2X_7_ receptor and the resultant Ca^2+^ influx in cancer^31,35^. Our research confirmed that the blockade of eATP synthase or of the P2X_7_ receptor reduced cytosolic Ca^2+^ concentration. The P2X_7_ receptor is displayed as an ATP-gated nonselective ion channel that is permeable to Ca^2+^. The evidence highlighted that Ca^2+^ in the cytosol was also derived from the P2X_7_ receptor in membrane channels and was stimulated by eATP synthase. Several reports have also previously concluded that the P2X_7_ receptor induces EV release^37,63^. Previous studies also show that eATP synthase influences cancer progression by affecting tumor proliferation, differentiation, and angiogenesis^5,17,65^. In our study, we found a mechanism describing the generation of ATP by eATP synthase under stress, which plays a critical role in triggering the release of EVs from cancer cells (Supplemental Fig. 8).

In the experiment exploring the distribution of large and small EV sizes, we confirmed that less than 10% of the total population had overlapping sizes, indicating that most of the large and small EVs were distinct (Fig. 1e). Our subsequent proteomic analysis of the L- and S-EVs further supported this finding. Among the 930 proteins identified in L-EVs and the 675 proteins identified in S-EVs, only 480 proteins were found to overlap, while the remaining 450 and 195 proteins were unique to L-EVs and S-EVs, respectively. This suggests that the L- and S-EVs in our experiment not only differ in size but also in their content. Additionally, the proteins up-regulated in response to starvation were found to differ between the two populations, with ATP5A being one example in our study. This led to differing impacts on immune cells, further demonstrating that the isolated L- and S-EVs were distinct vesicles. Other proteomic studies also showed that ATP synthase subunits were identified in EVs^86,87^, which corroborates our findings (Fig. 4). However, we also found that ATP5A was specifically enriched in S-EVs after starvation, which revealed that ATP synthase subunits were upregulated in S-EVs for specific functions during starvation. When comparing the results of proteomic analysis of cells and EVs, we found that some proteins only up-regulated in EVs under starvation conditions. This indicated that these proteins were intentionally packaged to EVs under starvation. The EVs derived from cancer cells suppresses various kinds of immune cells, as was previously reported^88,89^. Thus, the content of S-EVs may be the main factor inhibiting Jurkat T cell proliferation and cytokine secretion in the present study, and the increasing eATP synthase on S-EVs stimulated the uptake of them by immune cells. On the other hand, because we found that eATP synthase exists on EVs and interacts with Fyn, Fyn appeared to be a suitable target for the immunosuppression of Jurkat T cells. Fyn has been reported to influence T-cell activation, differentiation, and tolerance, via T cell receptor (TCR) stimulation^90–92^. Our data showed that only incubation with Fyn Ab significantly caused decreased the survival of Jurkat T cells (Supplemental Fig. 7d), suggesting that Fyn-ATP synthase interaction not only guides EV uptake, but also directly influences Jurkat T cell proliferation. According to previous studies, Fyn is a kinase whose structure mainly lies within the plasma membrane^93^. Since we have found that ATP synthase exists on EVs and interacts with Fyn, it is therefore unlikely that the ATP synthase on S-EVs interacts with Fyn on the cell surface. To verify the results, we selected the Fyn isoform related to T cells, based on the results of the proteome analysis, to confirm the possibility of its interaction with ATP synthase. Using transmembrane prediction and antibody labeling, we validated that a partial fragment of Fyn lies outside the membrane and predicted its interaction with ATP synthase. We further confirmed that blocking ATP synthase and Fyn reduced EV uptake. Although these results cannot definitively prove the interaction between ATP synthase and Fyn, they demonstrate that ATP synthase and Fyn on EV and T cells, respectively, directly affect EV uptake and have critical roles in EV communication (Supplemental Fig. 8).

## Methods

### Cell culture and conditioned medium

A549, SK-N-BE(2)C, and T47D cells were cultured in Dulbecco’s modified Eagle’s medium (DMEM; Gibco Laboratories, Grand Island, NY, USA) with 10% fetal bovine serum (FBS; Biological Industries, Kibbutz Beit Haemek, Israel) and incubated at 37 °C and 5% CO_2_ for 24 h. Following the one-day culture, the media was changed to DMEM supplemented with 0.1% exosome-depleted FBS for an additional 18 h (starvation treatment). The conditioned media was collected for further isolation and purification by differential centrifugation. EV-depleted FBS was obtained by ultra-centrifugation at 100,000×*g* for 18 h.

### Fluorescence staining and immunocytochemistry

Cells were cultured for 24 h and incubated with MitoTracker (1:10,000; Thermo Fisher Scientific, Waltham, MA, USA) for 30 min or Cell-Mask (1:100,00; Thermo Fisher Scientific). The cells were fixed in 4% paraformaldehyde (PFA; Sigma-Aldrich, St. Louis, MO, USA) for 15 min. The cells were probed with primary anti-ATP synthase complex antibodies (1:1000; Abcam, Cambridge, MA, USA) for 16 h at 4 °C. The cells were labeled with the corresponding secondary anti-mouse IgG-Alexa 488 antibodies (1:5000; Invitrogen, Carlsbad, CA, USA). The samples were then washed three times with PBS. The cell nuclei were stained with 4′,6-diamidino-2-phenylindole (DAPI; Invitrogen). The cells were analyzed with a Zeiss LSM780 confocal laser microscope (Zeiss, Oberkochen, Germany).

### Reduction, alkylation, and digestion of proteins

Cells or EVs were collected and proteins extracted in 12 mM sodium deoxycholate (Sigma), 50 mM triethylammonium bicarbonate (Sigma), 12 mM sodium lauroyl sarcosine, and protease inhibitor cocktail (BioShop, Burlington, Canada). The cells were sonicated by homogenizer (LABSONIC M ultrasonic homogenizer, Satorius AG, Göttingen, Germany). The supernatant was collected by centrifugation at 16,000×*g* for 20 min at 4 °C and the protein concentration was measured by Pierce BCA Protein Assay kit (Thermo Fisher Scientific). For each group, 500 μg cell lysates were prepared and 1 M triethylammonium bicarbonate buffer (TEABC; Sigma) was added to reach a final concentration of 50 mM TEABC. Protein samples were reduced by dithiothreitol (BioShop) in a 37 °C water bath for 30 min, and then were alkylated by 2 mM S-methyl methanethiosulfonate (MMTS; Sigma) in the dark at room temperature for 30 min. Next, the proteins were incubated with trypsin (Thermo Fisher Scientific) at 37 °C for 16 h.

### Dimethyl labeling of peptides

The peptides (100 μg) were dried and resolved in 300 μL of 100 mM TEABC solution. To the two groups of peptides, 16 μL of 4% (v/v) formaldehyde-H2, and 4% (v/v) formaldehyde-D2 (Sigma) were added, respectively. The mixtures then had 16 μL of freshly prepared 0.6 M sodium cyanoborohydride (Sigma) added as catalyst. After 1 h incubation, 64 μL of 1% ammonia (WAKO, Osaka, Japan) was added to the samples and they were placed on ice to stop the reaction. To further ensure the reaction had completely stopped, 32 μL of 10% (v/v) formic acid (Sigma) was added to acidify the sample to a pH of 3. Finally, the samples were combined in a 1:1 ratio (v:v).

### NanoLC-MS/MS analysis

This experiment was performed according to our previous studies^94^. The peptides were identified using NanoLC-MS/MS analysis with a nanoACQUITY UPLC (Waters, Milford, MA, USA) and an LTQ-Orbitrap XL (Thermo Electron, Bremen, Germany) system, as described previously. Peptide samples were loaded into a 2 cm × 180 μm capillary trap column and then separated in a 75 μm × 25 cm nanoACQUITY 1.7 μm BEH C18 column (Waters, Milford, MA) at a flow rate of 300 nL/min. Mobile phase A consisted of 0.1% formic acid, and mobile phase B consisted of 0.1% formic acid and 80% acetonitrile (ACN). A linear gradient of 10% to 40% B in 90 min and 40% to 85% B in 10 min was employed throughout this study. Mass spectra from the scans were acquired on the Orbitrap (*m*/*z* 350–1500). The resolution was set to 60,000 at *m*/*z* 400 and the automatic gain control (AGC) was set to 1 × 106 ions. The *m*/*z* values triggering the MS/MS were put on an exclusion list for 90 seconds. The top ten most intense precursor ions were selected from the MS scan for subsequent collision-induced dissociation MS/MS scan by ion trap (AGC target at 7000).

### Quantitative proteome data analysis

The raw MS data was analyzed for peak detection and quantification using MaxQuant software version 2.0.3.0 (Martinsried, Germany). The identified peptide sequences were analyzed with the Andromeda search engine and the Swiss-Prot database (released in April, 2021, subset human, 20,375 protein entries). Trypsin specificity, fixed modification of carbamidomethyl (C), and variable modifications of oxidation (M) were set as search criteria with allowing 2 missed cleavages and at least six amino acid. The type of label chose Dimethyl Lys0 and Dimethyl Nter0 in Light, and DIMETHYL LYS6 and DIMETHYL NTER6 in Heavy. The precursor mass tolerance was 3 ppm and the fragment ion tolerance was 0.5 Da. By using a decoy database strategy, peptide identification was accepted based on the posterior error probability with a false discovery rate of 1%. Precursor masses of already identified peptides were further searched and recalculated by using the “match between runs” option in MaxQuant. The data have been uploaded to the ProteomeXchange (http://www.proteomexchange.org/) with the dataset identifier PXD014995.

### DAVID GO enrichment analysis

The MaxQuant output file “proteinGroups.txt” was used for this analysis. The heavy/light ratio and intensity for each protein were loaded to Perseus software version 1.6.15.0 and calculated significant B of each protein as p-value. Using fold change >1.96 standard deviation and p-value < 0.05 as cut-off to filter out differential expression of proteins. The remaining 56 proteins (Supplementary Data 1. Red, Up-regulated. Blue, Down-regulated.) were then subjected as target set to analysis by DAVID (https://david.ncifcrf.gov/tools.jsp). The DAVID human genome database was used as background set and annotated with their GO terms.

### EV isolation

EVs contained in cell-conditioned medium produced from A549, SK-N-BE(2)C and T47D cells were isolated using differential centrifugation. To remove the suspended cells, the cell-conditioned medium was centrifuged at 300×*g* for 10 min. The supernatant medium was then centrifuged at 2000×*g* for 20 min to obtain apoptotic bodies. The supernatant was centrifuged at 15,000×*g* for 30 min thereafter to obtain large EVs. The remaining supernatant was then subjected to ultracentrifugation at 120,000×*g* for 70 min at 4 °C to obtain small EVs. Each type of EV was washed by resuspending with 20 ml 0.1 M PBS and stored at –80 °C.

### Nanoparticle tracking analysis

The cell culture supernatants containing EVs were analyzed using NanoSight NS300 (Duxbury, MA). Each sample was analyzed in triplicate by taking a 30-second video at a rate of 30 frames per second and at a camera level of 10 under 25 °C. Approximately 30–150 particles were analyzed in each field of view, and the recorded video was then analyzed to measure the distribution of particle sizes and particle concentrations. In addition, cells were collected, dyed with trypan blue (Invitrogen) and counted to assess cell viability to normalize EV quantification.

### Transmission electron microscopy

The EV pellets of each fraction were individually re-suspended in 0.1 M PBS with pH 7.4 and fixed with 4% PFA. For each fraction, 5 μL was added onto glow discharged 200 mesh formvar copper grids (EMSTM) and incubated for 1 min at room temperature. The grids were incubated with 0.1% glutaraldehyde (Sigma) in 0.1 M PBS for 5 min at room temperature. After being washed several times with 0.1 M PBS, they were negatively stained with 2% aqueous uranyl acetate (Sigma) for 1 min and blotted dry with filter paper. The grids were air dried for more than 20 min, before they were visualized with a Hitachi H-7650 transmission electron microscope (TEM) at 80 kV.

### Western blotting

The eEV pellets of each fraction were individually re-suspended in 0.1 M PBS with 1% Triton X-100 (Sigma), and then lysed by sonication. In all, 20 μg of each sample were resolved by 10% SDS-PAGE and transferred onto 0.45 μm polyvinyl-difluoride (PVDF) membranes (Millipore, Billerica, MA, USA). The PVDF membranes were blocked with 3% non-fat milk in PBST (PBS containing 0.05% Tween-20; Sigma) for 1 h. Anti-CD40, CD63, and CD81 (GeneTex, Irvine, CA, USA) primary antibodies were diluted in 3% milk/PBST and incubated with the membranes for 16 h at 4 °C. Anti-rabbit IgG (Abcam; 1:5000) or anti-mouse IgG (Abcam; 1:5000) secondary antibodies conjugated with horseradish peroxidase (HRP) enzyme were then incubated with the membranes for 1 h and were detected using a luminescent image analyzer (ProteinSimple, San Jose, CA, USA). The band intensities were quantified using ImageJ and normalized according to GAPDH level. All the western uncropped data were presented in Supplemental Figure 9.

### Mitochondrial image analysis

Confocal images were analyzed using Icy software (http://icy.bioimageanalysis.org/). First, we set the mitochondrial channel (Channel 1) and adjusted the image contrast to enhance the weak signals. Customized programming was set and the selected channel from imaged mitochondria was extracted. We further blurred images for the networked mitochondria by a Gaussian filter tool. The results were automatically adjusted by a k-means thresholder. Lastly, spots (i.e., mitochondria) were detected on the output-processed images using parameters of 100 for the specificity threshold and a 3-pixel detection scale. The perimeters, areas, and contour levels of each spot were displayed.

### Transfection of plasmid DNAs and selection

The shRNA against Drp1 (shRNA#1, ID: TRCN0000318424; target sequence: GCTACTTTACTCCAACTTATT; shRNA#2, ID: TRCN0000318426; target sequence: CGAGATTGTGAGGTTATTGAA) and the control vector (shRNA#CTR, ID: ASN0000000006) were obtained from the National RNAi Core Facility at the Genomic Research Center, Academia Sinica. Cells were optimal at 60%–80% confluence. The commercial shRNA against Drp1 or the control vector were introduced into cells by transfection using linear polyethyleneimine (AlfaAesar, Lancashire, UK), as per the manufacturer’s protocol. After 16–24 h of incubation, the medium was removed and replaced with fresh medium containing 1% puromycin (Selleckchem, USA). The cells were incubated for 48 h to remove non-transfected cells. PalmGFP constructed plasmid^95^ were transfected to A549, SK-N-BE(2)C, and T47D by linear polyethyleneimine as mentioned above.

### ATP bioluminescence assay

The levels of extracellular ATP secreted by A549, SK-N-BE(2)C, and T47D cells were assayed with a bioluminescence assay kit (Sigma) according to the manual. Cultures of 1 × 10^4^ cells of each type were incubated for 24 h. The cells were refreshed with medium containing 2 μM citreoviridin (Cayman Chemical Company, Ann Arbor, MI, USA) or dimethyl sulfoxide (DMSO, sigma) for 24 h at 37 °C. Next, 200 μM ADP (Sigma) was added for 10 min. The concentration of extracellular ATP was determined using the bioluminescence assay kit. In Brief, we added 100 μl ATP assay mix solution to the assay tube and stand for 3 min at room temperature for hydrolyzing endogenous ATP. ATP standard solution were diluted with DMEM from 10^−3^ to 10^−7^ moles/liter. In all, 100 μl of each sample and standard were added and the plate was read immediately using a luminometer. For the starvation experiments, 1 × 10^4^ cells of each type were seeded in 12-well plates and incubated for 24 h. The cells were refreshed with medium containing 10% or 0.1% FBS for a further 18 h incubation.

### Calcium ion assay

Conditioned cells were washed twice with PBS and re-suspended in lysis buffer (0.1 M phosphate buffer with 1% Triton X-100 (Sigma)), before being lysed by sonication. The calcium in the cell lysate was then measured using a calcium assay kit (Abcam). Briefly, the calcium standard was diluted with H_2_O. Then, 50 μL of each standard and sample were loaded into 96-well plate. The lysis buffer was also loaded into 96-well plate as blank. Next, 50 μL of assay reagent mixture was added to each well. After being incubated for 20 min at room temperature, the fluorescence intensity was detected with an ELISA reader (Ex/Em = 540/590 nm).

### Inhibitor treatments

ATP (Sigma) was solubilized in nuclease-free water to create a stock concentration of 25 mM. Cells were treated with 200 μM ATP for 6 h. A740003, a competitive P2X_7_ receptor antagonist (MCE), was solubilized in DMSO (Sigma) at a stock concentration of 5 mM. Cells were pre-treated with 20 μM A740003 for 24 h. Citreoviridin (Cayman Chemical Company) is an impermeable ATP synthase inhibitor. We solubilized citreoviridin in DMSO to create a stock concentration of 40 mM, before diluting it down to 2 μM for cell treatment. Inhibitors of mitochondrial fission markers Drp1 and mdivi-1 (Sigma) were solubilized in DMSO at a stock concentration of 50 mM, before being diluted to 30 μM for cell treatments lasting 24 h.

### Dot blot

The freshly collected EVs were directly dropped onto the 0.45 μm nitrocellulose membrane (GE healthcare). After blocking with 5% BSA in PBS for 1 h, the membrane was incubated with the primary antibody diluted in 5% BSA/PBS for 16 h at 4 °C. The membranes were then incubated with horseradish peroxidase (HRP)-conjugated secondary antibody for 1 h at room temperature and detected by a luminescent image analyzer (ProteinSimple, San Jose, California, USA).

### ATP5B protein construction, amplification, and purification

Total RNA was extracted from MCF-7 cells and reverse-transcribed into its complementary DNA (cDNA) using cDNA Synthesis Kit (MBI Fermentas, Vilnius, Lithuania). The full-length human *ATP5B* was amplified from the cDNA by polymerase chain reaction (PCR; *KAPA* Biosystems, Cape Town, South Africa) using two sets of oligonucleotide primers (Genomics, Hsinchu, Taiwan) listed below:

Forward, 5′-GGGGAATTCATGTTGGGGTTTGTGGGTCGG-3′

Reverse, 5′-GGGCTCGAG**TCA**CGATGAATGCTCTTCAGC-3′

Forward, 5′-GGGGAATTCATGTTGGGGTTTGTGGGTCGG-3′

Reverse, 5′-GGGCTCGAGCGATGAATGCTCTTCAGCCAG-3′

Both sets of primers incorporated the EcoRI (New England Biolabs, Beverly, MA, USA) and XhoI (New England Biolabs) restriction sites (underlined). The first set of oligonucleotide primers was designed with a translational stop codon (bold). The PCR program was set as follows: 95 °C for 5 min, followed by 35 cycles of denaturation at 98 °C for 20 s, annealing at 65 °C for 15 s, and extension at 72 °C for 55 s, and a final extension at 72 °C for 5 min. The PCR product was isolated by electrophoresis on a 1% agarose gel and observed by staining with a nucleic acid stain (Seeing Bioscience, Taipei, Taiwan). The *ATP5B* PCR products were digested with *EcoRI* and *XhoI* and ligated (RBC Bioscience, Taipei, Taiwan) into the pET-22b (+), pET-43.1a (+), pGEX-4T1, and pMAL-c2X plasmids, which were digested with the corresponding enzymes. All the ligated plasmids were transformed into *E. coli* DH5α cells (RBC Bioscience) and selected on Luria-Bertani (LB) agar plates containing 50 μg/ml ampicillin (Bioshop). The size of the construct was confirmed by agarose gel electrophoresis after digestion by *EcoRI* and *XhoI*. Constructs with the correctly sized DNA inserts were sequenced (Genomics) to ensure mutation-free PCR products. Finally, the sequence-verified constructs were transformed into *E. coli* BL21-codonplus competent cells or *E. coli* BL21-codonplus-RIL competent cells to allow induced expression of ATP5B. To express the recombinant protein, liquid culture was grown in LB medium supplemented with 50 μg/ml ampicillin and incubated overnight at 37 °C with 200 rpm shaking. Subsequently, the overnight culture was inoculated into fresh medium in a 3:100 (v/v) dilution. Cells were grown at 37 °C with 200 rpm shaking until absorbance at 600 nm (A_600_) reached 0.4–0.6. To induce the production of recombinant proteins, 0.1 mM isopropyl-b-D-thiogalactopyranoside (IPTG; Bioshop) was supplemented for 5 h at 37 °C with 140 rpm shaking. Finally, cells were harvested by centrifugation at 4000 rpm for 20 min at 4 °C. Pellets were resuspended in PBS and disrupted by sonication (20 s sonication followed by 10 sec rest, repeated for a total of 10 times) on ice. The lysates were centrifuged at 12,500 rpm for 20 min at 4 °C. The majority of the ATP5B proteins were contained within the insoluble pellet fraction as inclusion bodies. As a result, pellets were washed after centrifugation to remove the unbroken cells. The pellet containing inclusion bodies from the 1 L original culture was resuspended and denatured in 10 ml PBS containing 6 M guanidine hydrochloride (JT Baker, PA, United States*)*. The samples were centrifuged at 125,000 rpm for 15 min at 4 °C. The clear supernatant was diluted to a concentration of 3 M urea (Bioshop) with PBS and transferred to a dialysis membrane (Membrane Filtration Products, Inc., San Antonia, TX, USA*)*. The denatured protein samples were initially dialyzed at a 1:25 ratio against PBS containing 2 M urea. Afterwards, dialysis was performed in several steps, and in each step the urea concentration in PBS was decreased as follows: 2, 1, 0.5, 0.25, and 0.125 M. Protein dialysis was performed in at least 25–50 volumes of dialysis buffer for 6–12 h. Finally, the sample was dialyzed twice against 100 volumes of PBS in the absence of urea. Refolding occurred during dialysis at 4 °C with slow-speed stirring. After dialysis, the protein samples were centrifuged at 12,500 rpm for 15 min at 4 °C, and the remaining pellets were collected and dialyzed once again.

### Probing of ATP5B with a human proteome microarray

The human proteome microarray chip, containing 16368 human proteins (purchased from CDI Labs, Baltimore, USA), was blocked with 3% BSA in Tris-Buffered Saline and Tween-20 (TBST) buffer for 2 h with gentle shaking. Subsequently, 80 μl of ATP5B protein (1 μg/μl) was added to the chip, which was incubated in the hybridization chamber on a 3D shaker for 1 h at 37 °C. After the 1-h hybridization, the chip was washed with TBST buffer at room temperature for 5 min and then incubated with a 1500-fold diluted anti-ATP5B antibody (Abcam) for 1 h. The chip was then washed with TBST buffer for another 5 min and incubated with a 150,000-fold diluted anti-mouse IgG antibody labeled with Cy-3 at 25 °C for 30 min. Finally, the chip was washed three times with TBST buffer for 5 min and spun dry. The chip was scanned with a microarray scanner (Axon GenePix® 4000B; Axon Instrument, Union City, CA) and analyzed using the GenePix Pro 6.0 software (Molecular Devices, Sunnyvale, CA, USA).

### Protein-protein docking simulation

The sequences of human ATP synthase subunits and tyrosine-protein kinase Fyn (isoform Fyn-T) were obtained from UniProt (https://www.uniprot.org/) and constructed by SWISS-MODEL (http://swissmodel.expasy.org/)^73^. Protein-protein docking simulation were performed using ClusPro 2.0 (https://cluspro.bu.edu/)^74^. PMPCA and FANCE were also constructed by SWISS-MODEL and used as the control group for docking with ATP synthase. From 70,000 rotations, 1000 rotation/position combinations with the lowest scores were chosen with a 9 Å C-alpha root mean square deviation radius. The results were visualized using PyMOL (http://pymol.org/). The best binding models for each ligand obtained from docking were analyzed by LigPlot^+^ 2.2.5 to display the interaction diagrams^96^.

### Transmembrane protein predictions

The Fyn-T protein sequence was obtained from UniProt (https://www.uniprot.org/). Membrain, which was developed by the pattern recognition and bioinformatics group of Shanghai Jiao Tong university (http://www.csbio.sjtu.edu.cn/bioinf/MemBrain/)^97^, was also used to predict the transmembrane domain of Fyn-T. We choose “α-TMP Topology prediction using deep learning” with “transmembrane helix prediction”. The N-term signal peptide was chosen unknown and human protein were selected.

### Treatment of Jurkat T-cells with EVs

To block ATP synthase on the surface of EVs or Fyn on Jukat T cell, 20 μg of EVs derived from cancer cell lines were incubated with an anti-ATP synthase complex antibody (10 μg/ml, Abcam) or IgG isotype antibodies (10 μg/ml, Abcam) in 50 μl PBS at 4 °C for 16 h. The EVs were then washed with 1.5 ml PBS and pelleted by ultracentrifugation to remove any non-bound free antibodies. Jurkat T cell were activated by 0.2% of the Cell Activation Cocktail (Biolegend, San Diego, California, USA.) in media for 6 h incubation. Activated Jurkat T cells, 1 × 10^4^ cells were incubated with an anti-Fyn antibody (10 μg/ml, GeneTex) or IgG isotype antibodies (10 μg/ml, Abcam) in media for 1 h at 37 °C, and then washed three times with PBS. The EVs were incubated directly with activated Jurkat T cells for 48 h. After incubation, the Jurkat cells were collected, stained with trypan blue, and counted to determine cell viability.

### Cytokine assay

The cytokine levels were quantified using IL-2 and INF-γ ELISA kits (Biolegend). To perform the assay, 100 μl of capture antibodies were coated onto ELISA plates and incubated at 4 °C for 16 h. The plates were then blocked with 200 μl of Assay Diluent A per well for 1 h at room temperature. Next, we added 100 μl of conditioned media per well and incubated for 2 h at room temperature. After incubation with 100 μl of detection antibody, we added 100 μl of avidin-HRP buffer per well for 30 min. Finally, the plates were incubated with 100 μl of substrate buffer per well for 20 min and the cytokine levels were detected using an ELISA reader.

### EV uptake detection in Jurkat T cell

We first incubated 20 μg palmGFP-labeled S-EVs with either an anti-ATP synthase complex antibody or IgG (10 μg/ml) in 50 μl PBS at 37 °C for 1 h, and the 1 × 10^4^ Jurkat T cells were incubated with anti-Fyn antibody or IgG (10 μg/ml) in media at 37 °C for 1 h. Then, incubated the Jurkat T cell with palmGFP-labeled S-EVs for 1 h. The sample were observed by confocal microscope and the intensity of the images was measured using ZEN2009 software. The values represent the mean ± SD (*N* = 30).

### Resource

All detailed information on the chemicals, antibodies, and reagents used is listed in the Supplementary Data 6.

### Statistics and reproducibility

The data were expressed as the mean ± SD of experimental replicates and subjected to Student’s unpaired two-tailed *t*-test to analyze their statistical significance. Differences were considered significant at a *p*-value of <0.05. The sample size used for each statistical calculation were indicated in the Figure legend.

### Reporting summary

Further information on research design is available in the Nature Portfolio Reporting Summary linked to this article.

## Supplementary information

Supplementary Information

Description of Additional Supplementary Files

supplementary data 1

supplementary data 2

supplementary data 3

supplementary data 4

supplementary data 5

supplementary data 6

supplementary data 7

Reporting Summary

## Data Availability

All original mass spectrometry data have been deposited to the ProteomeXchange (Project accession PXD014995). All the western uncropped data were presented in Supplemental Figure 9. The source data behind the graphs can be found in Supplementary Data 7. Other data supporting our study are available from the corresponding author on reasonable request.
