# Supplementary material for: Ectopic ATP synthase stimulates the secretion of extracellular vesicles in cancer cells
Source: Commun Biol. 2023 Jun 15;6:642. doi: 10.1038/s42003-023-05008-5 (PMC10272197; doi:10.1038/s42003-023-05008-5)
Supplement: Supplementary file 1 — Supplementary Information [file 42003_2023_5008_MOESM1_ESM.pdf]

Supplemental figures

Ectopic ATP synthase stimulates the secretion of extracellular vesicles in cancer cells

Yi-Chun Kao[1], Yi-Wen Chang[1], Charles P. Lai[2], Nai-Wen Chang[3], Chen-Hao Huang[4], Chien-Sheng Chen[5], Hsuan-Cheng Huang[6,*], and Hsueh-Fen Juan [1,3,4,7,*]

[1]Department of Life Science, National Taiwan University, Taipei 106, Taiwan.

[2]Institute of Atomic and Molecular Sciences, Academia Sinica, Taipei 106, Taiwan.

[3]Institute of Molecular and Cellular Biology, National Taiwan University, Taipei 106, Taiwan.

[4]Graduate Institute of Biomedical Electronics and Bioinformatics, National Taiwan University, Taipei 106, Taiwan.

[5] Department of Food Safety / Hygiene and Risk Management, National Cheng Kung University, Tainan, Taiwan

[6]Institute of Biomedical Informatics, National Yang Ming Chiao Tung University, Taipei 112, Taiwan.

[7]Center for Computational and Systems Biology, National Taiwan University, Taipei 106, Taiwan.

*Correspondence:

Hsuan-Cheng Huang, Ph.D.

Institute of Biomedical Informatics, National Yang Ming Chiao Tung University, No.155, Sec.2, Linong Street, Taipei 11221, Taiwan; Tel: +886-2-28267357; Fax: +886-2-28202508; E-mail: hsuancheng@nycu.edu.tw

Hsueh-Fen Juan, Ph.D.

Department of Life Science, Graduate Institute of Biomedical Electronics and Bioinformatics, National Taiwan University, No. 1, Sec. 4, Roosevelt Rd., 106 Taipei, Taiwan. Tel: +886-2-3366-4536; Fax: +886-2-23673374; E-mail: yukijuan@ntu.edu.tw

Supplemental Fig. 1

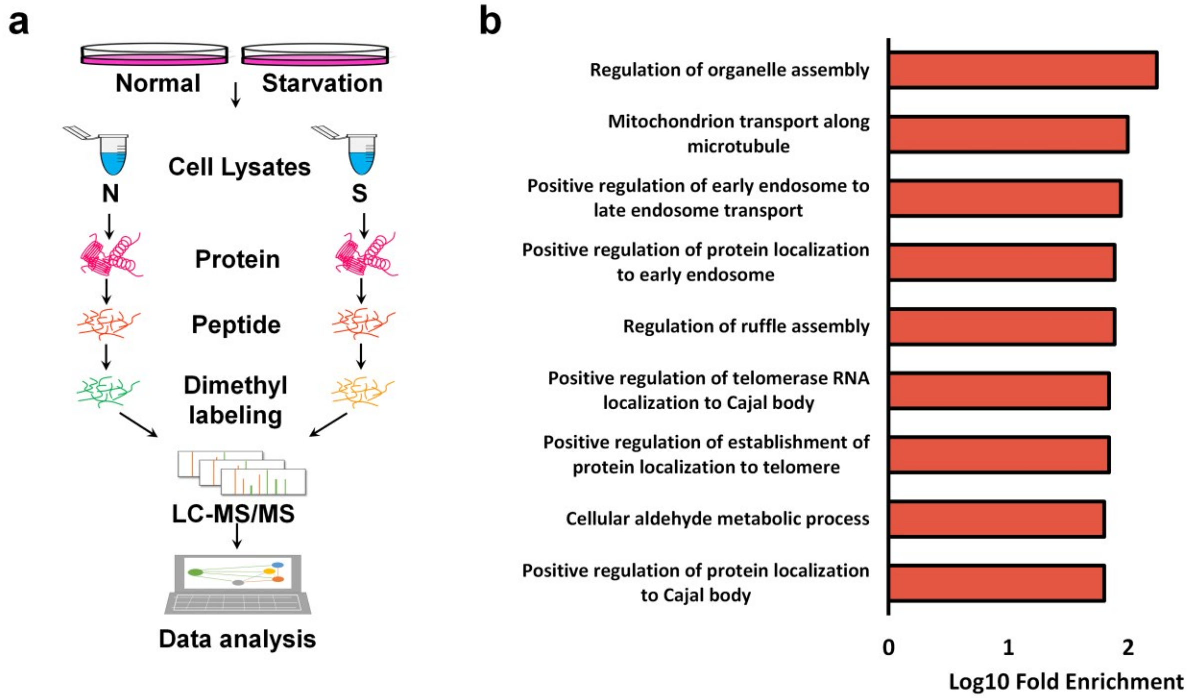

Supplemental Fig. 1: Quantitative proteomics of A549 under starvation treatment. (a) The total proteins in 18 hours starvation-treated and control A549 cells were digested and underwent dimethyl labeling (Starvation labeled heavy, Normal label light). Mixed peptides were analyzed using mass spectrometry and quantified by MaxQuant software. The data were further analyzed by DAVID (6.8 version) gene ontology. (b) Horizontal bar chart of GO term enrichment (biological process).

Supplemental Fig. 2

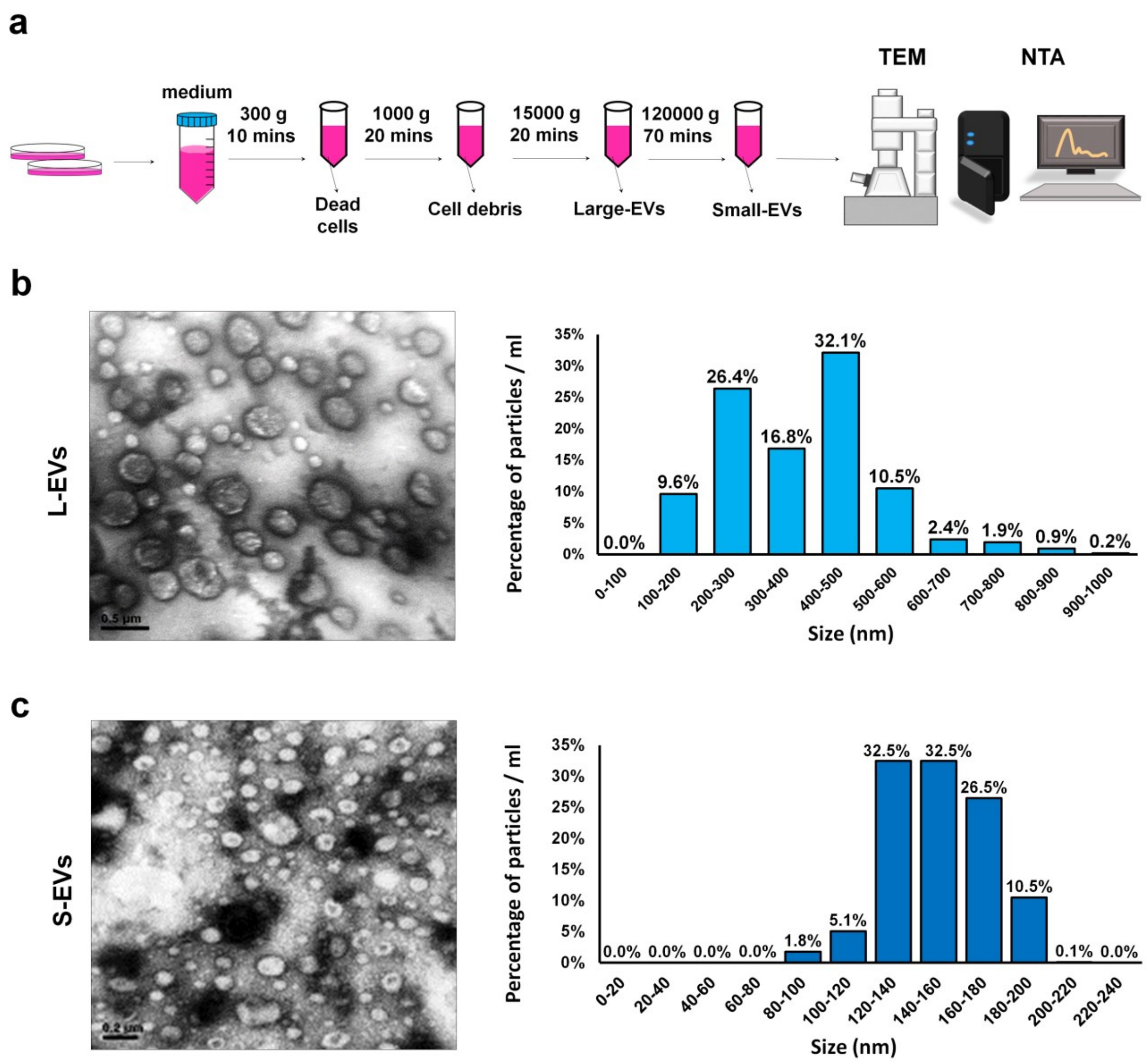

Supplemental Fig. 2: Characterization of extracellular vesicles.

(a) Flowchart of L- and S-EVs isolation. (b-c) L-EVs and S-EVs from cell-conditioned media were isolated and characterized using transmission electron microscopy and nanoparticle tracking analysis, respectively.

Supplemental Fig. 3

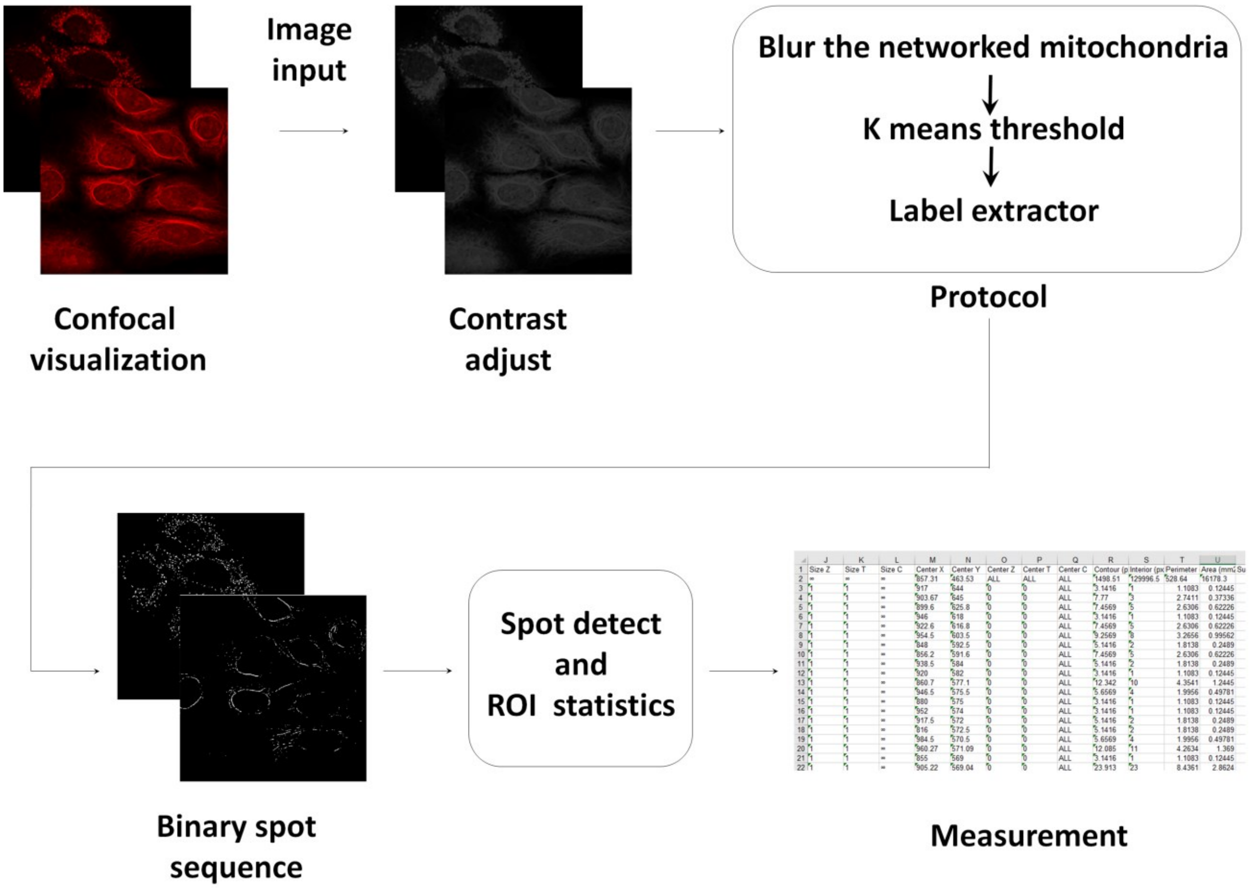

Supplemental Fig. 3: Overview of the Icy software pipeline.

Confocal images labeled with mitochondrial fluorescence were loaded into the Icy software. The contrast was adjusted to enhance the signal. The images were further blurred for networked mitochondria with a Gaussian filter tool and the results were loaded into a k-means thresholder for automatic adjustment. After the pictures were adjusted to create a binary threshold, the processed images were loaded for spot detection and the ROIs (regions of interest) were automatically calculated. The results show the areas, perimeters, and contour levels of each detected spot.

Supplemental Fig. 4

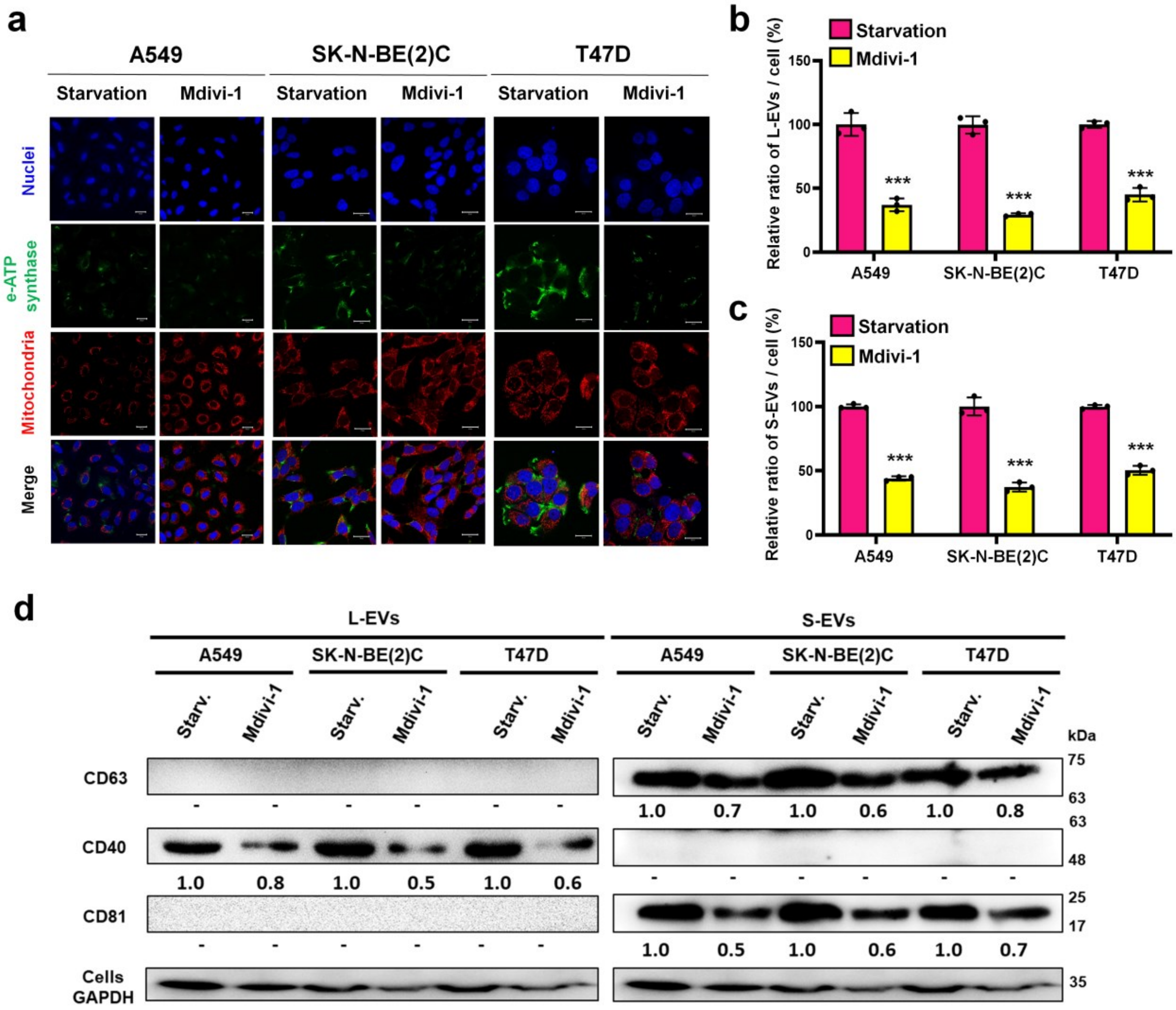

Supplemental Fig. 4: EV release decreases after Drp1 is inhibited.

(a) A549, SK-N-BE(2)C and T47D cells treated with 30 μM mdivi-1 or DMSO under starvation for 24 hours and visualized using immunocytochemistry. Scale bar, 20 μm. (b-d). A549, SK-N-BE(2)C and T47D cells were treated with 30 μM mdivi-1 or DMSO for 24 hours under starvation and EVs were isolated from media and quantified by NTA and western blot. The values represent the mean ± SD (n=3).

Supplemental Fig. 5

a

Gene symbol	Description	Chip1	Chip2
FYN	Proto-oncogene tyrosine-protein kinase Fyn		
PGK1	phosphoglycerate kinase 1		
PTS	6-pyruvoyl-tetrahydropterin synthase		
CPNE6	copine VI		
ACYP1	acylphosphatase 1		
LRRFIP1	leucine rich repeat (in FLII) interacting protein 1		
TNIK	TRAF2 and NCK interacting kinase		
ANXA11	annexin A11		
DNMT3A	DNA (cytosine-5-) -methyltransferase 3 alpha		
MAPK12	mitogen-activated protein kinase 12		
NAP1L1	nucleosome assembly protein 1-like 1		
THYN1	thymocyte nuclear protein 1		
BTF3L4	basic transcription factor 3-like 4		
ANKRD22	ankyrin repeat domain 22		
MAGEB1	melanoma antigen family B, 1		
NUDT9	nudix (nucleoside diphosphate linked moiety X)-type motif 9		

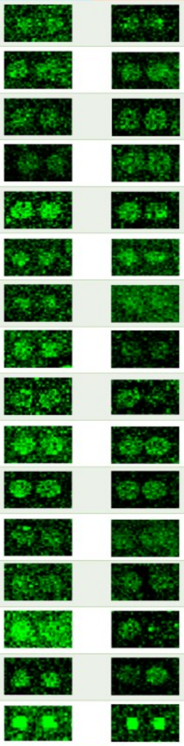

b

Ligand	Docking visualization	Score (kcal/mol)
FYN	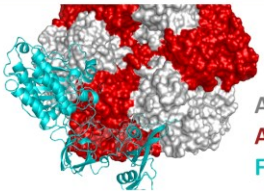 ATP synthase α subunit<br>ATP synthase β subunit<br>FYN	-4384.3
PMPCA	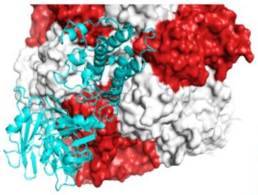 ATP synthase α subunit<br>ATP synthase β subunit<br>PMPCA	-988.7
FANCE	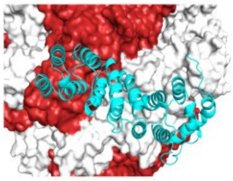 ATP synthase α subunit<br>ATP synthase β subunit<br>FANCE	-732.5

Supplemental Fig. 5: Candidates of ATP5B interacting proteins.

(a) List of 16 overlap protein candidates between respective replication of human proteome array. (b) Fyn, PMPCA (positive control) and FANCE (negative control) were constructed by SWISS-MODEL and dock to ATP synthase. The best binding model for each ligand obtained from docking was visualized using PyMOL. The predicted lowest weight score of each model was showed (kcal/mol).

Supplemental Fig. 6

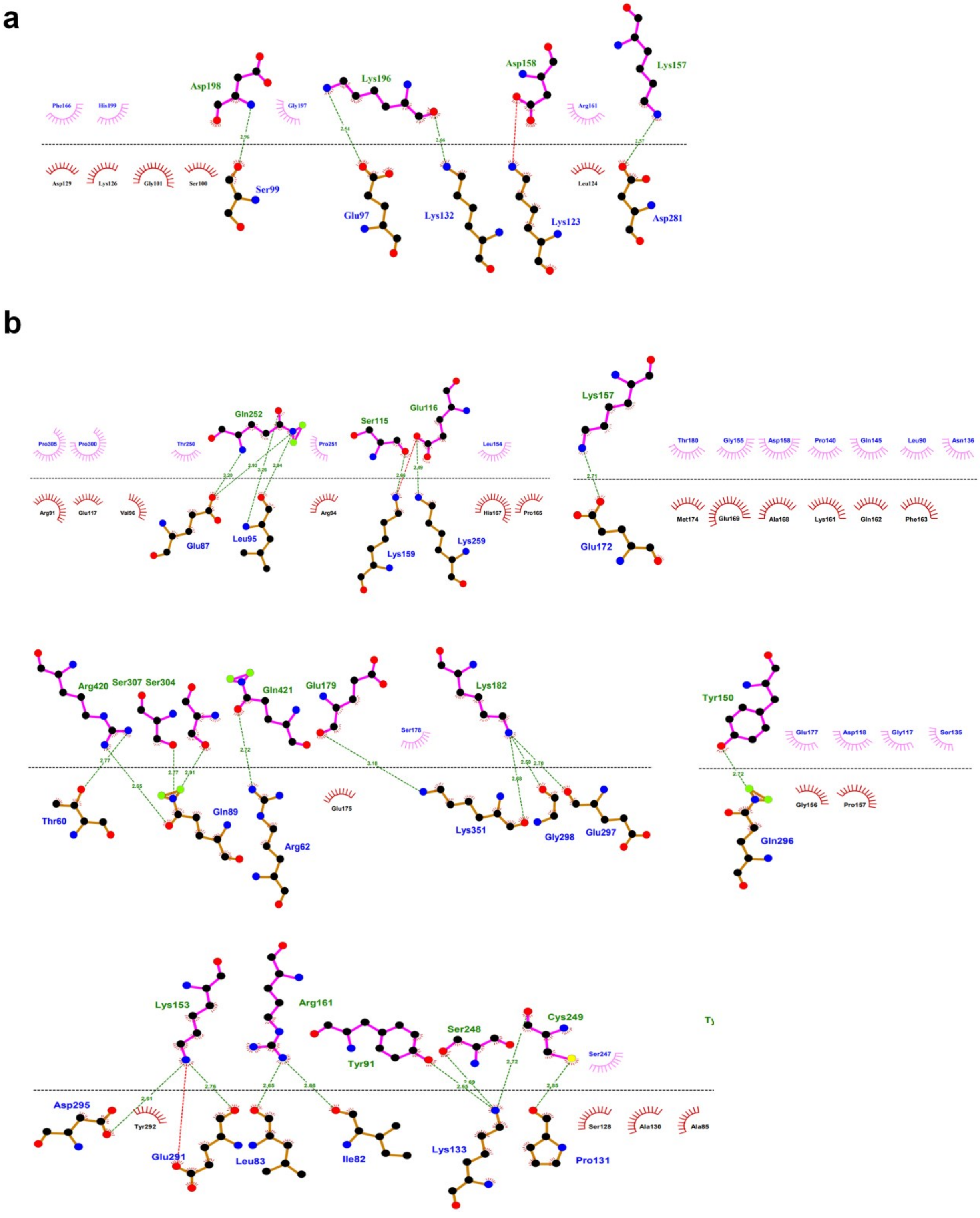

Supplemental Fig. 6: Interaction analysis of ATP synthase to Fyn-T.

(a-b) LigPlot interaction diagrams of the best binding models of ATP5A (a) and ATP5B (b) with Fyn-T. Structure of ATP5A and ATP5B were presented on the black line and Fyn were below the black line. Green dotted lines are hydrogen bond interactions and red. Pink semicircles are the hydrophobic interactions.

Supplemental Fig. 7

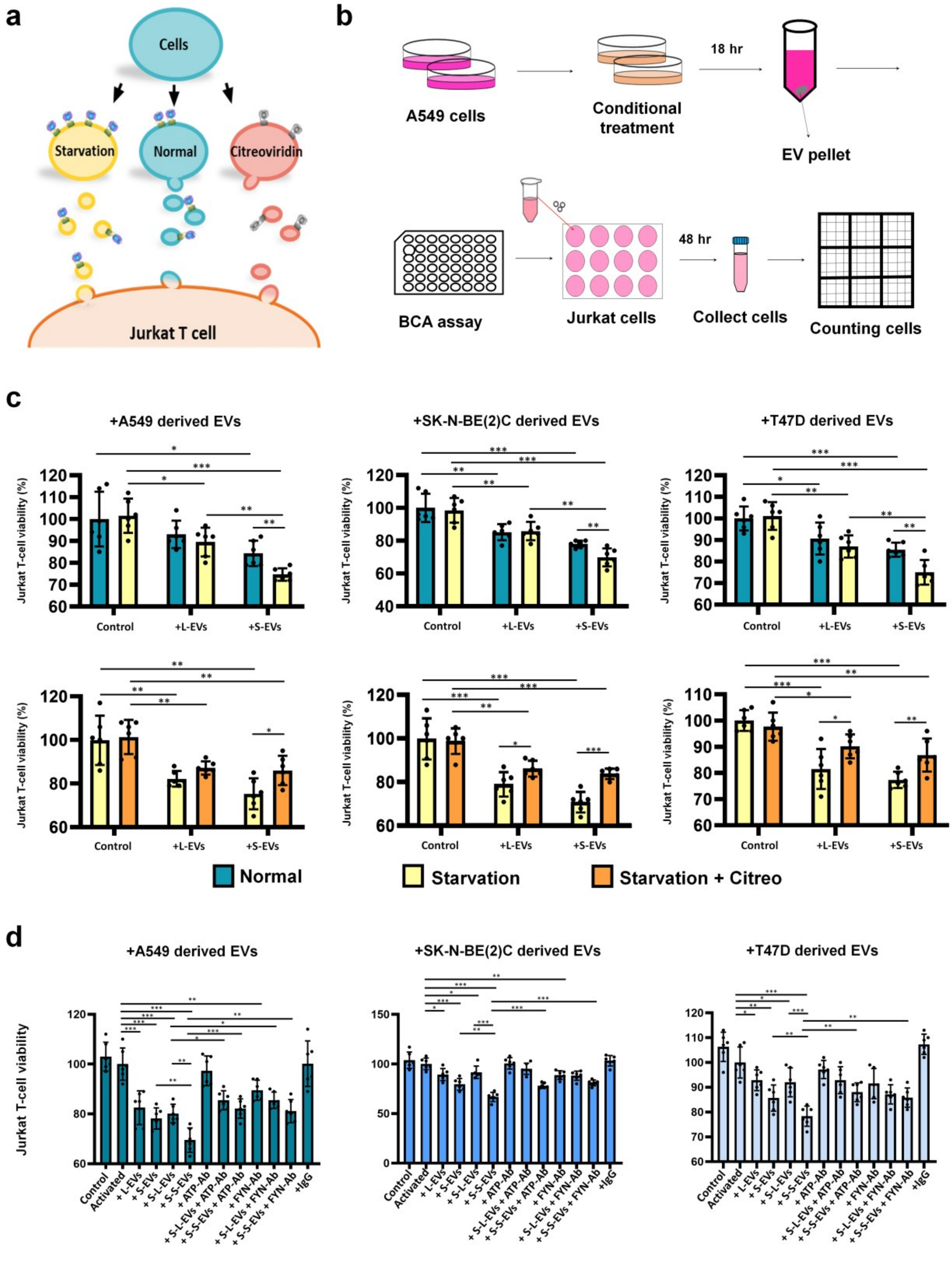

Supplemental Fig. 7: S-EVs derived from cancer cells under starvation reduced survival of Jurkat T-cells.

(a-b) Flowchart and schematic of treatment and proliferation assay of Jurkat T-cells. (c) 20μg L- or S-EVs derived from A549, SK-N-BE(2)C and T47D culture medium which had been treated with DMEM containing 10% FBS or 0.1% FBS for 18 hours or 2μM citreoviridin for 24 hours were washed by PBS and added to activated Jurkat T cells. After incubation for 48 hours, Jurkat cells were collected and dyed with trypan blue, then counted cells to calculate the viability. The values represent the mean ± SD (n=3). (d) The L- or S-EVs derived from A549, SK-N-BE(2)C, and T47D were incubated with the anti-ATP synthase complex antibody, and then added to activated Jurkat T cells. On the other hand, activated Jurkat T cells ($1x10^4$) were incubated with the anti-Fyn antibody, and then incubated with L- or S-EVs derived from A549, SK-N-BE(2)C, and T47D. After incubation for 48 hours, Jurkat cells were collected and dyed with trypan blue, then counted cells to calculate the viability. The values represent the mean ± SD (n=6).

Supplemental Fig. 8

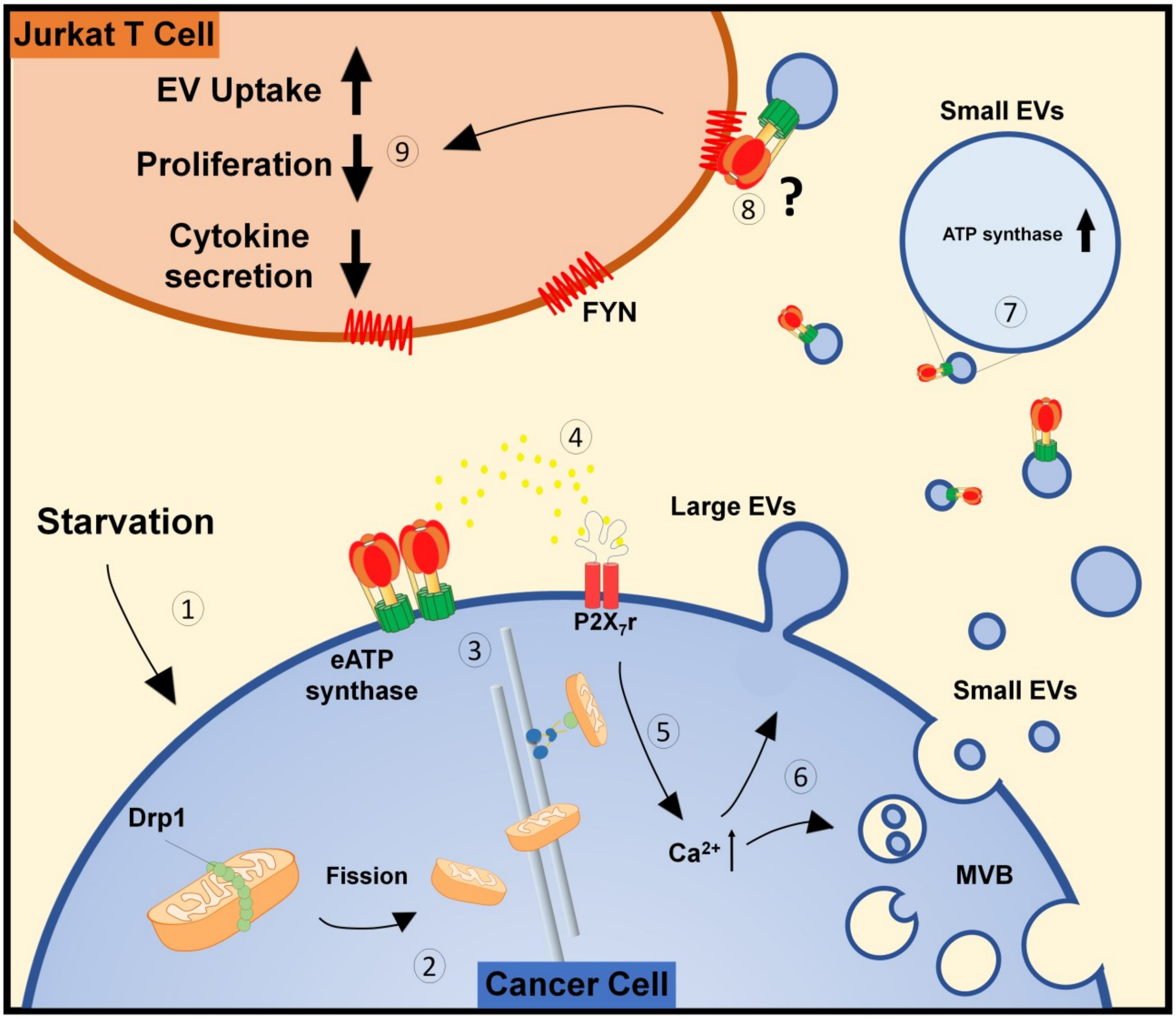

Supplemental Fig. 8: Proposed model for the role of ectopic ATP synthase in cell communication via extracellular vesicles.

(1) Serum starvation alters the mitochondrial function. (2) Mitochondria fission occurs. (3) The mitochondrial fission protein, Drp1, increases the expression of eATP synthase on the cell surface. (4) eATP synthase on the cell surface produces ATP in the extracellular space, and the ATP is utilized by the $P2X_7$ receptor. (5) After stimulation by ATP, the P2X7 receptor induces a Ca^{2+} influx. (6) The Ca^{2+} influx triggers extracellular vesicle secretion. (7) S-EVs derived from cancer cells carried more ATP synthase on the surface.

(8) ATP synthase on EVs may interact to Fyn on Jurkat T cell. (9) ATP synthase on S-EVs and Fyn on Jurkat T-cell contributed on EV uptake and decreasing proliferation and cytokine secretion of Jurkat T cell.

Supplemental Fig. 9

Figure S4D

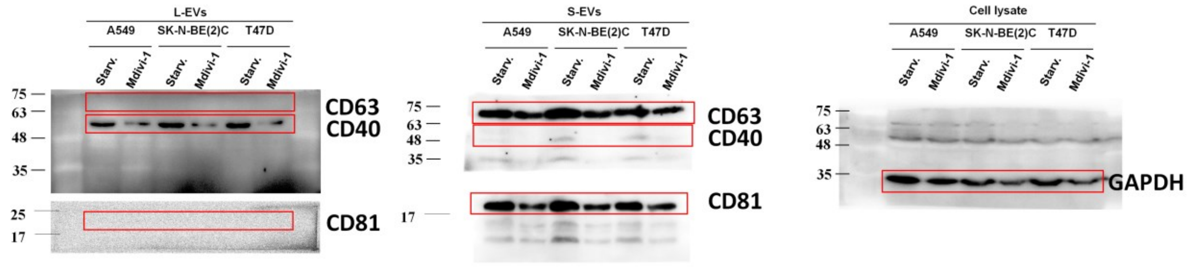

Figure 1F

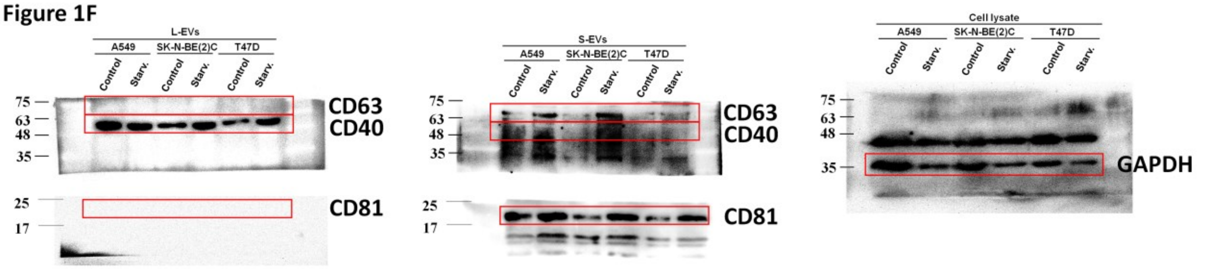

Figure 2C

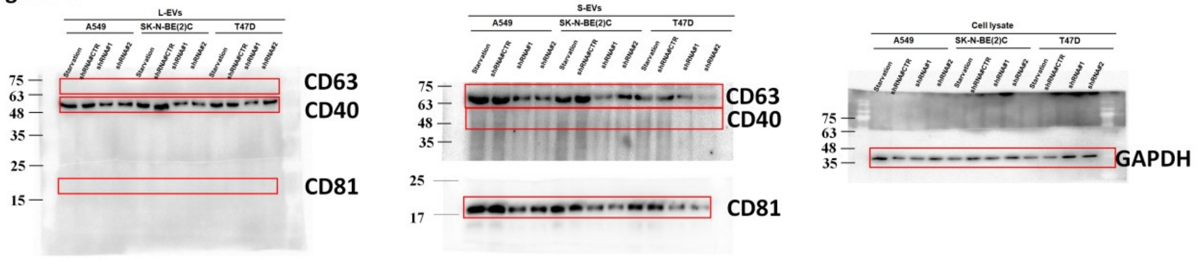

Figure 3D

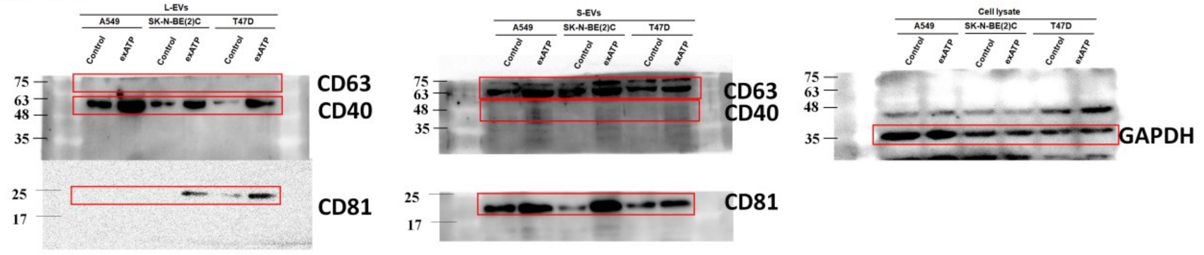

Figure 5D

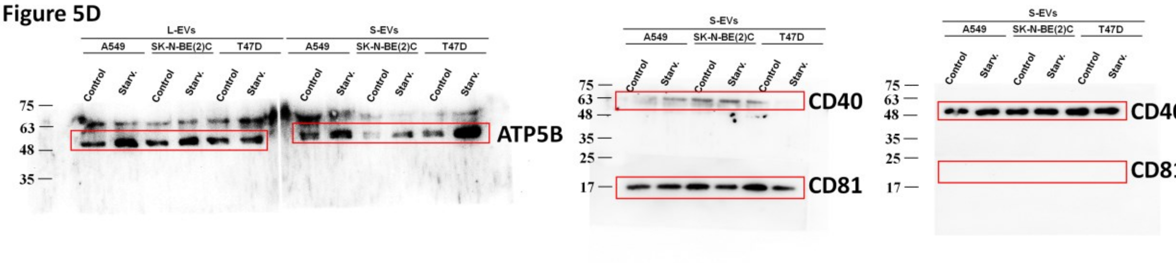

Figure 3F

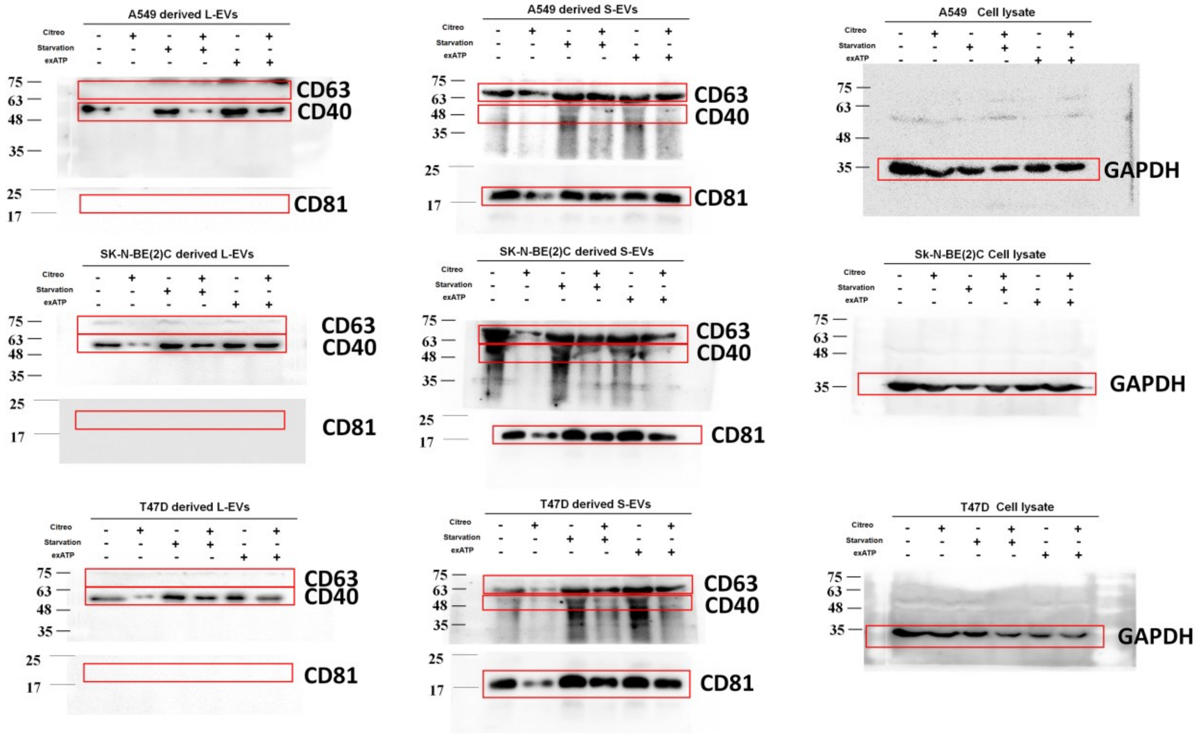

Figure 4A

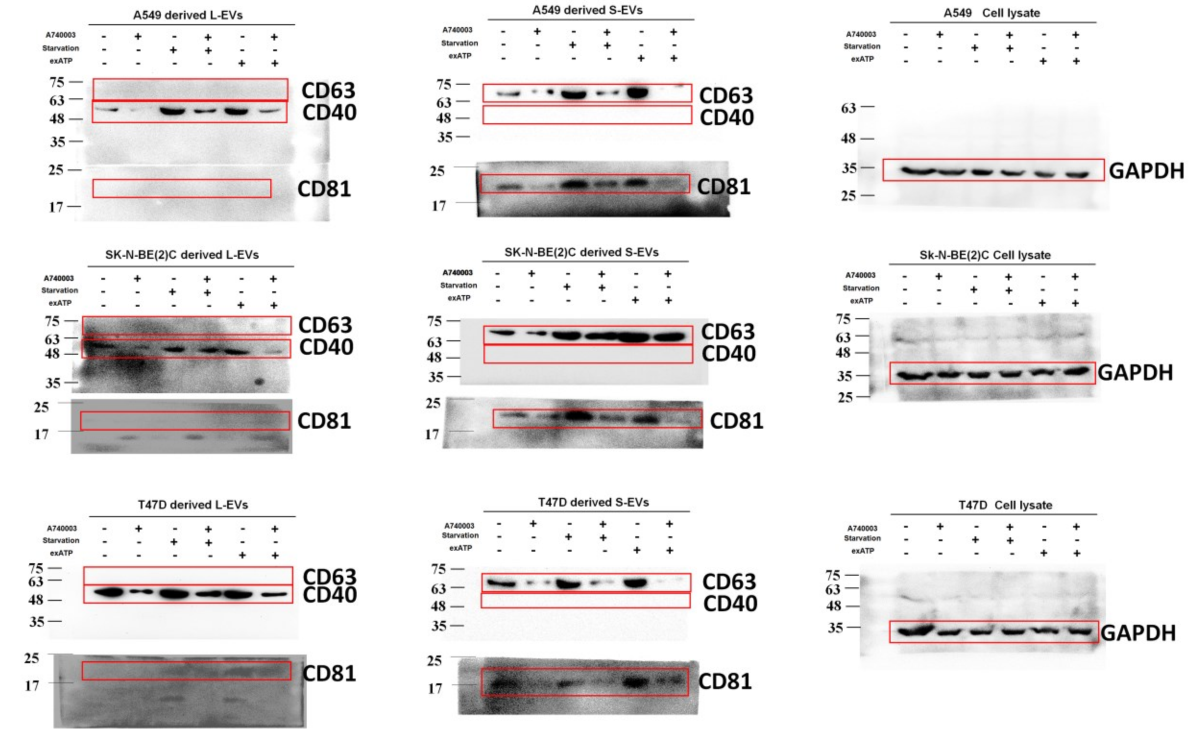

Supplemental Fig. 9: Full images of immunoblots.

Full uncropped images and signals detected by immunoblotting, with the regions used in the corresponding main figure panels indicated by red frames.
