# Supplementary material for: Ectopic ATP synthase stimulates the secretion of extracellular vesicles in cancer cells
Source: Commun Biol. 2023 Jun 15;6:642. doi: 10.1038/s42003-023-05008-5 (PMC10272197; doi:10.1038/s42003-023-05008-5)
Supplement: Supplementary file 2 — Description of Additional Supplementary Files [file 42003_2023_5008_MOESM2_ESM.pdf]

Description of Additional Supplementary Files

File name: Supplementary Data 1

Description: Detailed proteomics data of 305 quantified proteins in A549

File name: Supplementary Data 2

Description: Detailed GO analysis data of 56 differential expressed proteins in A549

File name: Supplementary Data 3

Description: Detailed proteomics data of 123 quantified proteins in L-EVs derived from A549

File name: Supplementary Data 4

Description: Detailed proteomics data of 84 quantified proteins in S-EVs derived from A549

File name: Supplementary Data 5

Description: Transmembrane prediction of Fyn-T

File name: Supplementary Data 6

Description: Key resource table

File name: Supplementary Data 7

Description: Raw data of graphs and charts
