# Supplementary material for: Ectopic ATP synthase stimulates the secretion of extracellular vesicles in cancer cells
Source: Commun Biol. 2023 Jun 15;6:642. doi: 10.1038/s42003-023-05008-5 (PMC10272197; doi:10.1038/s42003-023-05008-5)
Supplement: Supplementary file 3 — supplementary data 1 [file 42003_2023_5008_MOESM3_ESM.docx]

| **Supplementary Data 1. Detailed proteomics data of 305 quantified proteins in A549** | | | | | | | | | |
| --- | --- | --- | --- | --- | --- | --- | --- | --- | --- |
| **Protein ID** | **Protein**  **name** | **Peptide**  **counts (all)** | **Unique peptides** | **Sequence coverage [%]** | **Sequence length** | **p-value** | **Ratio H/L** | **Ratio H/L normalized** | **Intensity** |
| **P11216** | **glycogen phosphorylase B(PYGB)** | **17;4** | **15** | **27.8** | **843** | **1.2E-137** | **13.548** | **14.094** | **4.9E+09** |
| **P06733** | **enolase 1(ENO1)** | **13;1** | **12** | **46.8** | **434** | **9.9E-137** | **13.783** | **14.050** | **2.3E+10** |
| **P60842** | **eukaryotic translation initiation factor 4A1(EIF4A1)** | **10;4** | **9** | **32** | **406** | **6.8E-115** | **13.185** | **12.942** | **3.4E+09** |
| **P05198** | **eukaryotic translation initiation factor 2 subunit alpha(EIF2S1)** | **5** | **5** | **29.2** | **315** | **1.6E-74** | **10.174** | **10.565** | **5.0E+08** |
| **P09874** | **poly(ADP-ribose) polymerase 1(PARP1)** | **7** | **7** | **10** | **1014** | **5.4E-71** | **10.423** | **10.328** | **2.8E+09** |
| **P29692** | **eukaryotic translation elongation factor 1 delta(EEF1D)** | **6** | **6** | **35.9** | **281** | **1.6E-37** | **8.415** | **7.689** | **2.4E+09** |
| **P68104** | **eukaryotic translation elongation factor 1 alpha 1(EEF1A1)** | **13;12** | **7** | **39** | **462** | **1.3E-31** | **6.231** | **7.109** | **1.3E+10** |
| **P48643** | **chaperonin containing TCP1 subunit 5(CCT5)** | **7** | **7** | **15.5** | **541** | **6.7E-25** | **7.797** | **6.377** | **4.8E+08** |
| **P00390** | **glutathione-disulfide reductase(GSR)** | **7** | **7** | **24.9** | **522** | **1.7E-16** | **5.143** | **5.292** | **2.5E+09** |
| **P68366** | **tubulin alpha 4a(TUBA4A)** | **9** | **2** | **31** | **448** | **3.0E-13** | **4.994** | **4.793** | **1.7E+09** |
| **O15371** | **eukaryotic translation initiation factor 3 subunit D(EIF3D)** | **3** | **3** | **6.2** | **548** | **1.9E-08** | **4.908** | **3.913** | **1.3E+09** |
| **P49748** | **acyl-CoA dehydrogenase very long chain(ACADVL)** | **9** | **9** | **16.9** | **655** | **1.9E-08** | **3.610** | **3.913** | **1.7E+09** |
| **Q9Y6N5** | **sulfide quinone oxidoreductase(SQOR)** | **6** | **6** | **16.2** | **450** | **8.2E-08** | **4.499** | **3.776** | **9.5E+08** |
| **Q86VP6** | **cullin associated and neddylation dissociated 1(CAND1)** | **15** | **15** | **17.3** | **1230** | **2.2E-07** | **3.346** | **3.681** | **2.2E+09** |
| **P42704** | **leucine rich pentatricopeptide repeat containing(LRPPRC)** | **27** | **27** | **26.7** | **1394** | **5.5E-07** | **3.663** | **3.590** | **2.8E+09** |
| **Q9Y295** | **developmentally regulated GTP binding protein 1(DRG1)** | **3** | **3** | **10.4** | **367** | **2.4E-06** | **3.132** | **3.436** | **5.0E+08** |
| **Q2TB90** | **hexokinase domain containing 1(HKDC1)** | **2** | **2** | **2.4** | **917** | **3.3E-06** | **3.567** | **3.400** | **3.7E+08** |
| **Q16891** | **inner membrane mitochondrial protein(IMMT)** | **10** | **10** | **22.3** | **758** | **5.4E-05** | **3.020** | **3.080** | **2.4E+09** |
| **P40939** | **hydroxyacyl-CoA dehydrogenase trifunctional multienzyme complex subunit alpha(HADHA)** | **18** | **18** | **36.6** | **763** | **1.2E-04** | **3.042** | **2.976** | **2.4E+09** |
| **Q8NBQ5** | **hydroxysteroid 17-beta dehydrogenase 11(HSD17B11)** | **2** | **2** | **12** | **300** | **1.6E-04** | **2.618** | **2.943** | **2.9E+08** |
| **P27824** | **calnexin(CANX)** | **9** | **9** | **21.3** | **592** | **3.6E-04** | **2.992** | **2.830** | **2.5E+09** |
| **P50914** | **ribosomal protein L14(RPL14)** | **2** | **2** | **11.2** | **215** | **4.5E-04** | **2.711** | **2.800** | **5.0E+09** |
| **Q9NR45** | **N-acetylneuraminate synthase(NANS)** | **2** | **2** | **7.8** | **359** | **6.1E-04** | **2.911** | **2.757** | **1.9E+08** |
| **P39656** | **dolichyl-diphosphooligosaccharide--protein glycosyltransferase non-catalytic subunit(DDOST)** | **7** | **7** | **27** | **456** | **7.0E-04** | **2.920** | **2.737** | **2.1E+09** |
| **P52895** | **aldo-keto reductase family 1 member C2(AKR1C2)** | **15** | **4** | **57.3** | **323** | **2.2E-03** | **2.378** | **2.562** | **1.4E+10** |
| **P46821** | **microtubule associated protein 1B(MAP1B)** | **8** | **8** | **5.5** | **2468** | **2.6E-03** | **2.703** | **2.542** | **4.2E+08** |
| **Q9Y230** | **RuvB like AAA ATPase 2(RUVBL2)** | **7** | **7** | **16** | **463** | **3.0E-03** | **2.586** | **2.517** | **1.9E+09** |
| **P78371** | **chaperonin containing TCP1 subunit 2(CCT2)** | **15** | **15** | **45.6** | **535** | **4.5E-03** | **2.296** | **2.448** | **3.7E+09** |
| **P42166** | **thymopoietin(TMPO)** | **8** | **3** | **18.6** | **694** | **4.9E-03** | **2.789** | **2.433** | **9.4E+08** |
| **P12814** | **actinin alpha 1(ACTN1)** | **27;8;5** | **16** | **38.7** | **892** | **5.7E-03** | **2.217** | **2.408** | **2.9E+09** |
| **P07339** | **cathepsin D(CTSD)** | **4** | **4** | **14.3** | **412** | **1.1E-02** | **2.052** | **2.295** | **5.0E+08** |
| **Q92499** | **DEAD-box helicase 1(DDX1)** | **5** | **5** | **10.7** | **740** | **1.7E-02** | **1.238** | **2.212** | **6.1E+08** |
| **P46776** | **ribosomal protein L27a(RPL27A)** | **3** | **3** | **19.6** | **148** | **2.0E-02** | **2.283** | **2.175** | **2.9E+09** |
| **P68400** | **casein kinase 2 alpha 1(CSNK2A1)** | **5;4** | **5** | **20.2** | **391** | **2.4E-02** | **1.890** | **2.140** | **1.4E+09** |
| **Q14847** | **LIM and SH3 protein 1(LASP1)** | **5** | **5** | **34.1** | **261** | **4.3E-02** | **1.879** | **2.020** | **1.4E+09** |
| **P11177** | **pyruvate dehydrogenase E1 subunit beta(PDHB)** | **5** | **5** | **18.4** | **359** | **5.7E-02** | **1.932** | **1.958** | **6.5E+08** |
| **Q96HE7** | **endoplasmic reticulum oxidoreductase 1 alpha(ERO1A)** | **4** | **4** | **12.2** | **468** | **6.4E-02** | **1.883** | **1.927** | **5.8E+08** |
| **O00410** | **importin 5(IPO5)** | **10;2** | **10** | **12.9** | **1097** | **7.5E-02** | **1.785** | **1.892** | **8.9E+08** |
| **P49411** | **Tu translation elongation factor, mitochondrial(TUFM)** | **12** | **12** | **35** | **452** | **1.2E-01** | **1.773** | **1.768** | **3.2E+09** |
| **P23246** | **splicing factor proline and glutamine rich(SFPQ)** | **13** | **12** | **23.6** | **707** | **1.7E-01** | **1.562** | **1.682** | **4.0E+09** |
| **P50148** | **G protein subunit alpha q(GNAQ)** | **1** | **1** | **5.3** | **359** | **1.7E-01** | **1.565** | **1.668** | **4.4E+07** |
| **P09525** | **annexin A4(ANXA4)** | **10** | **10** | **37.6** | **319** | **1.8E-01** | **1.507** | **1.658** | **8.0E+09** |
| **P06744** | **glucose-6-phosphate isomerase(GPI)** | **13** | **9** | **30.8** | **558** | **1.9E-01** | **1.732** | **1.646** | **6.0E+09** |
| **O60306** | **aquarius intron-binding spliceosomal factor(AQR)** | **2** | **2** | **2.4** | **1485** | **2.6E-01** | **1.527** | **1.553** | **8.2E+07** |
| **Q16798** | **malic enzyme 3(ME3)** | **1** | **1** | **4.5** | **604** | **2.6E-01** | **1.491** | **1.541** | **3.7E+07** |
| **Q9NPD3** | **exosome component 4(EXOSC4)** | **2** | **2** | **10.6** | **245** | **2.8E-01** | **1.519** | **1.525** | **1.8E+08** |
| **P50395** | **GDP dissociation inhibitor 2(GDI2)** | **10** | **5** | **32.1** | **445** | **2.9E-01** | **1.432** | **1.505** | **3.0E+09** |
| **Q1KMD3** | **heterogeneous nuclear ribonucleoprotein U like 2(HNRNPUL2)** | **3** | **3** | **6.7** | **747** | **3.0E-01** | **1.460** | **1.503** | **4.0E+08** |
| **Q15008** | **proteasome 26S subunit, non-ATPase 6(PSMD6)** | **5** | **5** | **16.7** | **389** | **3.2E-01** | **1.429** | **1.480** | **6.7E+08** |
| **Q14697** | **glucosidase II alpha subunit(GANAB)** | **14** | **14** | **22.6** | **944** | **3.3E-01** | **1.479** | **1.469** | **2.4E+09** |
| **Q15393** | **splicing factor 3b subunit 3(SF3B3)** | **6** | **6** | **8.3** | **1217** | **3.3E-01** | **1.470** | **1.467** | **5.4E+08** |
| **Q14839** | **chromodomain helicase DNA binding protein 4(CHD4)** | **8;2;1** | **8** | **5.8** | **1912** | **3.4E-01** | **1.389** | **1.456** | **5.9E+07** |
| **P52209** | **phosphogluconate dehydrogenase(PGD)** | **10** | **10** | **26.7** | **483** | **3.4E-01** | **1.378** | **1.454** | **1.1E+10** |
| **P17655** | **calpain 2(CAPN2)** | **11** | **11** | **25.9** | **700** | **3.5E-01** | **1.414** | **1.449** | **1.1E+09** |
| **O60716** | **catenin delta 1(CTNND1)** | **3** | **3** | **4.2** | **968** | **3.7E-01** | **1.430** | **1.427** | **1.7E+09** |
| **O75874** | **isocitrate dehydrogenase (NADP(+)) 1(IDH1)** | **11** | **10** | **37.4** | **414** | **3.7E-01** | **1.328** | **1.425** | **1.4E+09** |
| **Q9Y5S9** | **RNA binding motif protein 8A(RBM8A)** | **3** | **3** | **31** | **174** | **3.8E-01** | **1.421** | **1.420** | **1.2E+08** |
| **P16435** | **cytochrome p450 oxidoreductase(POR)** | **5** | **5** | **11.4** | **677** | **3.8E-01** | **1.240** | **1.416** | **2.7E+08** |
| **P68363** | **tubulin alpha 1b(TUBA1B)** | **11;6;1** | **0** | **39.9** | **451** | **3.8E-01** | **1.290** | **1.414** | **2.4E+10** |
| **P13639** | **eukaryotic translation elongation factor 2(EEF2)** | **21** | **20** | **26.8** | **858** | **4.0E-01** | **1.192** | **1.394** | **9.0E+09** |
| **P23396** | **ribosomal protein S3(RPS3)** | **9** | **9** | **45.7** | **243** | **4.3E-01** | **1.111** | **1.368** | **4.2E+09** |
| **P60709** | **actin beta(ACTB)** | **14;14;5** | **5** | **48** | **375** | **4.6E-01** | **1.336** | **1.340** | **8.1E+10** |
| **O75348** | **ATPase H+ transporting V1 subunit G1(ATP6V1G1)** | **1** | **1** | **9.3** | **118** | **4.8E-01** | **1.145** | **1.326** | **7.5E+07** |
| **P61204** | **ADP ribosylation factor 3(ARF3)** | **7;7;4** | **3** | **47** | **181** | **4.8E-01** | **1.227** | **1.325** | **4.8E+09** |
| **P37108** | **signal recognition particle 14(SRP14)** | **1** | **1** | **10.3** | **136** | **4.8E-01** | **1.217** | **1.323** | **6.1E+08** |
| **Q16555** | **dihydropyrimidinase like 2(DPYSL2)** | **14;1** | **13** | **41.8** | **572** | **4.9E-01** | **1.267** | **1.316** | **3.0E+09** |
| **Q99880** | **H2B clustered histone 13(H2BC13)** | **5;5;5;5;5;5;5;5;5;3;1** | **1** | **37.3** | **126** | **5.1E-01** | **1.208** | **1.301** | **2.3E+10** |
| **O14617** | **adaptor related protein complex 3 subunit delta 1(AP3D1)** | **4** | **4** | **5.6** | **1153** | **5.3E-01** | **1.276** | **1.288** | **2.2E+08** |
| **P08758** | **annexin A5(ANXA5)** | **11** | **11** | **44.4** | **320** | **5.3E-01** | **1.186** | **1.288** | **4.4E+09** |
| **P62753** | **ribosomal protein S6(RPS6)** | **3** | **3** | **14.1** | **249** | **5.3E-01** | **1.281** | **1.283** | **1.7E+09** |
| **O60701** | **UDP-glucose 6-dehydrogenase(UGDH)** | **13** | **13** | **39.3** | **494** | **5.4E-01** | **1.160** | **1.280** | **8.5E+09** |
| **Q53FA7** | **tumor protein p53 inducible protein 3(TP53I3)** | **3** | **3** | **13** | **332** | **5.4E-01** | **1.158** | **1.273** | **5.4E+08** |
| **Q14974** | **karyopherin subunit beta 1(KPNB1)** | **17** | **17** | **25.5** | **876** | **5.5E-01** | **1.141** | **1.270** | **4.7E+09** |
| **Q96TA1** | **niban apoptosis regulator 2(NIBAN2)** | **8** | **8** | **18.5** | **746** | **5.5E-01** | **1.243** | **1.269** | **4.7E+08** |
| **P62854** | **ribosomal protein S26(RPS26)** | **2;1** | **2** | **20.9** | **115** | **5.5E-01** | **1.374** | **1.266** | **4.1E+09** |
| **P05783** | **keratin 18(KRT18)** | **30;3;2;2;2;1;1;1;1;1;1;1;1;1;1;1;1;1;1;1;1;1;1;1;1;1** | **21** | **70.9** | **430** | **5.6E-01** | **1.236** | **1.261** | **7.0E+10** |
| **P15559** | **NAD(P)H quinone dehydrogenase 1(NQO1)** | **9** | **9** | **35.4** | **274** | **5.6E-01** | **1.186** | **1.261** | **8.6E+09** |
| **Q5T4S7** | **ubiquitin protein ligase E3 component n-recognin 4(UBR4)** | **9** | **9** | **3.7** | **5183** | **5.8E-01** | **1.113** | **1.248** | **2.2E+08** |
| **P78347** | **general transcription factor IIi(GTF2I)** | **7** | **7** | **11.8** | **998** | **5.8E-01** | **1.192** | **1.246** | **5.1E+08** |
| **O60884** | **DnaJ heat shock protein family (Hsp40) member A2(DNAJA2)** | **3** | **3** | **17.7** | **412** | **5.8E-01** | **1.161** | **1.245** | **1.8E+08** |
| **Q08211** | **DExH-box helicase 9(DHX9)** | **14** | **14** | **14.7** | **1270** | **5.9E-01** | **1.109** | **1.239** | **4.8E+09** |
| **Q16181** | **septin 7(SEPTIN7)** | **3** | **3** | **12.4** | **437** | **5.9E-01** | **1.118** | **1.239** | **4.1E+08** |
| **Q14558** | **phosphoribosyl pyrophosphate synthetase associated protein 1(PRPSAP1)** | **3** | **3** | **12.9** | **356** | **5.9E-01** | **1.146** | **1.237** | **1.4E+08** |
| **P49327** | **fatty acid synthase(FASN)** | **34** | **34** | **21.6** | **2511** | **6.1E-01** | **1.217** | **1.223** | **5.6E+09** |
| **Q15019** | **septin 2(SEPTIN2)** | **6** | **6** | **25.2** | **361** | **6.2E-01** | **1.135** | **1.214** | **1.1E+09** |
| **P06737** | **glycogen phosphorylase L(PYGL)** | **9** | **7** | **17.1** | **847** | **6.3E-01** | **1.096** | **1.206** | **7.0E+08** |
| **Q00610** | **clathrin heavy chain(CLTC)** | **35** | **27** | **32.5** | **1675** | **6.4E-01** | **1.185** | **1.197** | **1.4E+10** |
| **P22087** | **fibrillarin(FBL)** | **2** | **1** | **15** | **321** | **6.5E-01** | **1.184** | **1.192** | **4.6E+08** |
| **P14625** | **heat shock protein 90 beta family member 1(HSP90B1)** | **23;2** | **22** | **31.8** | **803** | **6.6E-01** | **1.037** | **1.185** | **1.3E+10** |
| **Q14914** | **prostaglandin reductase 1(PTGR1)** | **8** | **8** | **29.2** | **329** | **6.6E-01** | **1.066** | **1.184** | **1.3E+09** |
| **Q9NY33** | **dipeptidyl peptidase 3(DPP3)** | **6** | **6** | **14.4** | **737** | **6.7E-01** | **1.127** | **1.180** | **1.8E+08** |
| **P12429** | **annexin A3(ANXA3)** | **8** | **8** | **34.4** | **323** | **6.7E-01** | **1.093** | **1.178** | **1.9E+09** |
| **P55769** | **small nuclear ribonucleoprotein 13(SNU13)** | **1** | **1** | **9.4** | **128** | **6.8E-01** | **1.015** | **1.175** | **1.7E+08** |
| **Q16836** | **hydroxyacyl-CoA dehydrogenase(HADH)** | **2** | **2** | **20.1** | **314** | **6.8E-01** | **1.164** | **1.169** | **1.2E+08** |
| **O75390** | **citrate synthase(CS)** | **4** | **4** | **14.8** | **466** | **6.9E-01** | **1.070** | **1.166** | **1.6E+09** |
| **Q15257** | **protein phosphatase 2 phosphatase activator(PTPA)** | **2** | **2** | **11.7** | **358** | **6.9E-01** | **1.156** | **1.163** | **3.0E+08** |
| **Q9UHD1** | **cysteine and histidine rich domain containing 1(CHORDC1)** | **2** | **2** | **7.5** | **332** | **6.9E-01** | **1.095** | **1.163** | **1.6E+08** |
| **P46109** | **CRK like proto-oncogene, adaptor protein(CRKL)** | **2** | **2** | **10.6** | **303** | **7.0E-01** | **1.086** | **1.155** | **1.8E+08** |
| **P14866** | **heterogeneous nuclear ribonucleoprotein L(HNRNPL)** | **7** | **7** | **28.2** | **589** | **7.0E-01** | **0.995** | **1.154** | **3.3E+09** |
| **Q9HDC9** | **adipocyte plasma membrane associated protein(APMAP)** | **4** | **4** | **18.3** | **416** | **7.1E-01** | **1.053** | **1.148** | **6.0E+08** |
| **Q14980** | **nuclear mitotic apparatus protein 1(NUMA1)** | **10** | **10** | **8.2** | **2115** | **7.2E-01** | **1.103** | **1.142** | **2.5E+08** |
| **P20700** | **lamin B1(LMNB1)** | **10** | **10** | **22.2** | **586** | **7.3E-01** | **1.040** | **1.136** | **1.2E+09** |
| **P31930** | **ubiquinol-cytochrome c reductase core protein 1(UQCRC1)** | **7** | **7** | **23.8** | **480** | **7.3E-01** | **1.050** | **1.136** | **1.0E+09** |
| **P26599** | **polypyrimidine tract binding protein 1(PTBP1)** | **7** | **7** | **31.6** | **531** | **7.3E-01** | **1.097** | **1.135** | **2.2E+09** |
| **Q96AJ9** | **vesicle transport through interaction with t-SNAREs 1A(VTI1A)** | **1** | **1** | **12.4** | **217** | **7.3E-01** | **1.105** | **1.133** | **4.3E+07** |
| **P41250** | **glycyl-tRNA synthetase 1(GARS1)** | **6** | **6** | **14.2** | **739** | **7.4E-01** | **1.032** | **1.131** | **2.9E+08** |
| **P25705** | **ATP synthase F1 subunit alpha(ATP5F1A)** | **16** | **16** | **41.6** | **553** | **7.4E-01** | **1.006** | **1.130** | **6.6E+09** |
| **P46940** | **IQ motif containing GTPase activating protein 1(IQGAP1)** | **30;1** | **30** | **28.2** | **1657** | **7.4E-01** | **1.115** | **1.128** | **3.8E+09** |
| **P11413** | **glucose-6-phosphate dehydrogenase(G6PD)** | **14** | **14** | **38.8** | **515** | **7.5E-01** | **0.927** | **1.124** | **1.1E+10** |
| **P63241** | **eukaryotic translation initiation factor 5A(EIF5A)** | **3;1;1** | **3** | **23.4** | **154** | **7.5E-01** | **1.023** | **1.121** | **6.8E+08** |
| **P21980** | **transglutaminase 2(TGM2)** | **10** | **10** | **23.1** | **687** | **7.6E-01** | **1.089** | **1.118** | **5.4E+09** |
| **Q01813** | **phosphofructokinase, platelet(PFKP)** | **17** | **14** | **27.9** | **784** | **7.6E-01** | **1.139** | **1.116** | **8.4E+09** |
| **O43169** | **cytochrome b5 type B(CYB5B)** | **4** | **4** | **49.3** | **150** | **7.6E-01** | **1.023** | **1.114** | **3.4E+09** |
| **O00159** | **myosin IC(MYO1C)** | **16** | **16** | **22.1** | **1063** | **7.6E-01** | **1.016** | **1.114** | **1.5E+09** |
| **P02751** | **fibronectin 1(FN1)** | **21** | **21** | **14.3** | **2477** | **7.7E-01** | **0.980** | **1.108** | **7.7E+09** |
| **Q8TCD5** | **5', 3'-nucleotidase, cytosolic(NT5C)** | **2** | **2** | **18.9** | **201** | **7.8E-01** | **1.072** | **1.102** | **1.7E+08** |
| **P55084** | **hydroxyacyl-CoA dehydrogenase trifunctional multienzyme complex subunit beta(HADHB)** | **4** | **4** | **13.5** | **474** | **7.8E-01** | **1.052** | **1.101** | **9.2E+08** |
| **P06576** | **ATP synthase F1 subunit beta(ATP5F1B)** | **19** | **19** | **64.1** | **529** | **7.8E-01** | **1.040** | **1.098** | **8.8E+09** |
| **Q01082** | **spectrin beta, non-erythrocytic 1(SPTBN1)** | **39;2;2** | **39** | **24** | **2364** | **7.9E-01** | **1.014** | **1.094** | **4.3E+09** |
| **P62847** | **ribosomal protein S24(RPS24)** | **3** | **3** | **28.6** | **133** | **7.9E-01** | **1.385** | **1.093** | **1.2E+09** |
| **Q12904** | **aminoacyl tRNA synthetase complex interacting multifunctional protein 1(AIMP1)** | **2** | **2** | **12.5** | **312** | **8.0E-01** | **1.048** | **1.090** | **8.8E+08** |
| **Q9NX58** | **Ly1 antibody reactive(LYAR)** | **3** | **3** | **13.7** | **379** | **8.3E-01** | **1.005** | **1.069** | **1.6E+08** |
| **P40925** | **malate dehydrogenase 1(MDH1)** | **6** | **6** | **22.2** | **334** | **8.3E-01** | **0.976** | **1.064** | **1.1E+09** |
| **P57678** | **gem nuclear organelle associated protein 4(GEMIN4)** | **1** | **1** | **1.4** | **1058** | **8.4E-01** | **1.054** | **1.061** | **5.1E+07** |
| **O00299** | **chloride intracellular channel 1(CLIC1)** | **6** | **6** | **35.7** | **241** | **8.4E-01** | **0.899** | **1.057** | **2.6E+09** |
| **Q12906** | **interleukin enhancer binding factor 3(ILF3)** | **14;2** | **14** | **23.5** | **894** | **8.5E-01** | **0.982** | **1.057** | **5.7E+09** |
| **P21333** | **filamin A(FLNA)** | **54** | **51** | **32.4** | **2647** | **8.5E-01** | **0.960** | **1.053** | **2.3E+10** |
| **Q07065** | **cytoskeleton associated protein 4(CKAP4)** | **12** | **12** | **27.9** | **602** | **8.5E-01** | **1.012** | **1.053** | **1.4E+09** |
| **P26641** | **eukaryotic translation elongation factor 1 gamma(EEF1G)** | **11** | **11** | **28.6** | **437** | **8.6E-01** | **0.937** | **1.048** | **2.8E+09** |
| **P13667** | **protein disulfide isomerase family A member 4(PDIA4)** | **13** | **13** | **26.2** | **645** | **8.6E-01** | **0.902** | **1.046** | **2.2E+09** |
| **O75533** | **splicing factor 3b subunit 1(SF3B1)** | **8** | **8** | **9.1** | **1304** | **8.7E-01** | **1.095** | **1.043** | **6.1E+08** |
| **P22695** | **ubiquinol-cytochrome c reductase core protein 2(UQCRC2)** | **7** | **7** | **27.2** | **453** | **8.7E-01** | **1.012** | **1.038** | **2.9E+09** |
| **Q9GZY8** | **mitochondrial fission factor(MFF)** | **1** | **1** | **7.9** | **342** | **8.8E-01** | **0.950** | **1.034** | **3.3E+08** |
| **P78527** | **protein kinase, DNA-activated, catalytic subunit(PRKDC)** | **41** | **41** | **13.8** | **4128** | **8.8E-01** | **1.307** | **1.031** | **7.6E+09** |
| **P17987** | **t-complex 1(TCP1)** | **10** | **10** | **27** | **556** | **9.0E-01** | **0.986** | **1.021** | **1.7E+09** |
| **P35580** | **myosin heavy chain 10(MYH10)** | **8** | **3** | **5.1** | **1976** | **9.0E-01** | **0.988** | **1.021** | **1.2E+08** |
| **P26640** | **valyl-tRNA synthetase 1(VARS1)** | **5** | **5** | **6** | **1264** | **9.1E-01** | **0.960** | **1.013** | **2.5E+08** |
| **Q9Y490** | **talin 1(TLN1)** | **29;2** | **29** | **18.9** | **2541** | **9.2E-01** | **0.979** | **1.005** | **3.0E+09** |
| **P22102** | **phosphoribosylglycinamide formyltransferase, phosphoribosylglycinamide synthetase, phosphoribosylaminoimidazole synthetase(GART)** | **6** | **6** | **9.3** | **1010** | **9.3E-01** | **0.818** | **1.003** | **4.5E+08** |
| **Q12931** | **TNF receptor associated protein 1(TRAP1)** | **11** | **11** | **23.7** | **704** | **9.4E-01** | **0.961** | **0.992** | **5.0E+08** |
| **P49902** | **5'-nucleotidase, cytosolic II(NT5C2)** | **7** | **7** | **21.9** | **561** | **9.6E-01** | **0.971** | **0.983** | **4.9E+08** |
| **P30101** | **protein disulfide isomerase family A member 3(PDIA3)** | **14** | **14** | **35.4** | **505** | **9.6E-01** | **0.890** | **0.981** | **5.0E+09** |
| **P25786** | **proteasome 20S subunit alpha 1(PSMA1)** | **6** | **6** | **32.7** | **263** | **9.6E-01** | **0.922** | **0.978** | **1.5E+09** |
| **P04843** | **ribophorin I(RPN1)** | **17** | **17** | **43.5** | **607** | **9.7E-01** | **0.952** | **0.974** | **4.1E+09** |
| **Q14126** | **desmoglein 2(DSG2)** | **2** | **2** | **3.9** | **1118** | **9.7E-01** | **0.951** | **0.971** | **2.4E+08** |
| **Q13409** | **dynein cytoplasmic 1 intermediate chain 2(DYNC1I2)** | **3** | **3** | **8.3** | **638** | **9.8E-01** | **0.908** | **0.966** | **8.9E+07** |
| **P84098** | **ribosomal protein L19(RPL19)** | **3** | **3** | **17.3** | **196** | **9.9E-01** | **0.841** | **0.964** | **1.0E+09** |
| **O00429** | **dynamin 1 like(DNM1L)** | **6** | **6** | **16.8** | **736** | **9.9E-01** | **0.874** | **0.962** | **2.6E+08** |
| **Q3ZCQ8** | **translocase of inner mitochondrial membrane 50(TIMM50)** | **3** | **3** | **12.5** | **353** | **9.9E-01** | **0.832** | **0.958** | **2.1E+08** |
| **Q15149** | **plectin(PLEC)** | **110;3** | **109** | **31** | **4684** | **1.0E+00** | **0.884** | **0.957** | **2.9E+10** |
| **Q9NR31** | **secretion associated Ras related GTPase 1A(SAR1A)** | **2** | **2** | **28.8** | **198** | **1.0E+00** | **0.950** | **0.956** | **2.1E+08** |
| **Q16881** | **thioredoxin reductase 1(TXNRD1)** | **19** | **19** | **40.4** | **649** | **1.0E+00** | **0.877** | **0.955** | **1.1E+10** |
| **P10809** | **heat shock protein family D (Hsp60) member 1(HSPD1)** | **20** | **20** | **51.1** | **573** | **1.0E+00** | **0.858** | **0.954** | **1.5E+10** |
| **P22314** | **ubiquitin like modifier activating enzyme 1(UBA1)** | **18** | **18** | **27** | **1058** | **9.9E-01** | **0.909** | **0.950** | **3.2E+09** |
| **Q15813** | **tubulin folding cofactor E(TBCE)** | **6** | **6** | **19** | **527** | **9.9E-01** | **0.873** | **0.949** | **1.7E+08** |
| **P30043** | **biliverdin reductase B(BLVRB)** | **6** | **6** | **47.1** | **206** | **9.7E-01** | **0.816** | **0.944** | **4.1E+09** |
| **Q12769** | **nucleoporin 160(NUP160)** | **3** | **3** | **4** | **1436** | **9.7E-01** | **0.917** | **0.943** | **2.9E+07** |
| **P10253** | **alpha glucosidase(GAA)** | **5** | **5** | **11.4** | **952** | **9.6E-01** | **0.926** | **0.937** | **2.2E+08** |
| **Q15477** | **SKI2 subunit of superkiller complex(SKIC2)** | **2** | **2** | **2.2** | **1246** | **9.5E-01** | **0.880** | **0.936** | **9.2E+07** |
| **Q9BSJ8** | **extended synaptotagmin 1(ESYT1)** | **7** | **7** | **9.8** | **1104** | **9.5E-01** | **0.900** | **0.935** | **4.9E+08** |
| **P29401** | **transketolase(TKT)** | **15** | **15** | **34.5** | **623** | **9.5E-01** | **0.816** | **0.934** | **1.6E+10** |
| **P30048** | **peroxiredoxin 3(PRDX3)** | **6** | **6** | **28.1** | **256** | **9.5E-01** | **0.982** | **0.934** | **7.4E+08** |
| **P27708** | **carbamoyl-phosphate synthetase 2, aspartate transcarbamylase, and dihydroorotase(CAD)** | **11** | **11** | **9.6** | **2225** | **9.4E-01** | **0.827** | **0.932** | **1.1E+09** |
| **O60218** | **aldo-keto reductase family 1 member B10(AKR1B10)** | **12** | **6** | **43.4** | **316** | **9.4E-01** | **0.767** | **0.930** | **2.5E+10** |
| **P29218** | **inositol monophosphatase 1(IMPA1)** | **1** | **1** | **8.3** | **277** | **9.4E-01** | **0.901** | **0.929** | **6.3E+07** |
| **O95299** | **NADH:ubiquinone oxidoreductase subunit A10(NDUFA10)** | **2** | **2** | **13.2** | **355** | **9.4E-01** | **0.784** | **0.929** | **7.1E+08** |
| **Q07960** | **Rho GTPase activating protein 1(ARHGAP1)** | **7** | **7** | **24.1** | **439** | **9.4E-01** | **0.885** | **0.929** | **6.3E+08** |
| **P51665** | **proteasome 26S subunit, non-ATPase 7(PSMD7)** | **5** | **5** | **28.1** | **324** | **9.1E-01** | **0.870** | **0.920** | **1.5E+09** |
| **P04844** | **ribophorin II(RPN2)** | **4** | **4** | **12.8** | **631** | **9.1E-01** | **0.890** | **0.918** | **6.3E+08** |
| **P05141** | **solute carrier family 25 member 5(SLC25A5)** | **10;5;3** | **3** | **29.9** | **298** | **9.0E-01** | **0.831** | **0.915** | **4.2E+09** |
| **P09960** | **leukotriene A4 hydrolase(LTA4H)** | **11** | **11** | **25.4** | **611** | **8.9E-01** | **0.916** | **0.912** | **1.2E+09** |
| **P55795** | **heterogeneous nuclear ribonucleoprotein H2(HNRNPH2)** | **4** | **1** | **13.4** | **449** | **8.9E-01** | **0.900** | **0.910** | **6.4E+07** |
| **P42330** | **aldo-keto reductase family 1 member C3(AKR1C3)** | **15;5** | **8** | **52.9** | **323** | **8.9E-01** | **0.816** | **0.909** | **2.8E+10** |
| **P17812** | **CTP synthase 1(CTPS1)** | **9;2** | **9** | **22.3** | **591** | **8.8E-01** | **0.850** | **0.908** | **8.1E+08** |
| **P25325** | **mercaptopyruvate sulfurtransferase(MPST)** | **4** | **4** | **25.6** | **297** | **8.8E-01** | **0.857** | **0.905** | **3.2E+08** |
| **Q99714** | **hydroxysteroid 17-beta dehydrogenase 10(HSD17B10)** | **7** | **7** | **39.8** | **261** | **8.7E-01** | **0.826** | **0.905** | **8.6E+08** |
| **A1L0T0** | **ilvB acetolactate synthase like(ILVBL)** | **1** | **1** | **3.6** | **632** | **8.7E-01** | **0.858** | **0.904** | **1.9E+07** |
| **P08670** | **vimentin(VIM)** | **26;4;4;1;1;1;1** | **24** | **58.4** | **466** | **8.4E-01** | **0.816** | **0.893** | **1.3E+10** |
| **P09211** | **glutathione S-transferase pi 1(GSTP1)** | **6** | **6** | **38.6** | **210** | **8.4E-01** | **0.756** | **0.890** | **3.8E+09** |
| **P35579** | **myosin heavy chain 9(MYH9)** | **52;1** | **44** | **34.4** | **1960** | **8.3E-01** | **0.836** | **0.887** | **1.8E+10** |
| **P0DMV9** | **heat shock protein family A (Hsp70) member 1B(HSPA1B)** | **14;14** | **7** | **31.2** | **641** | **8.2E-01** | **0.776** | **0.885** | **4.1E+09** |
| **P62333** | **proteasome 26S subunit, ATPase 6(PSMC6)** | **4** | **4** | **14.7** | **389** | **8.2E-01** | **0.831** | **0.883** | **4.5E+08** |
| **P31943** | **heterogeneous nuclear ribonucleoprotein H1(HNRNPH1)** | **5** | **1** | **17.8** | **449** | **8.2E-01** | **0.848** | **0.882** | **1.5E+09** |
| **Q7Z417** | **nuclear FMR1 interacting protein 2(NUFIP2)** | **1** | **1** | **4.2** | **695** | **8.1E-01** | **0.816** | **0.881** | **2.2E+06** |
| **Q15084** | **protein disulfide isomerase family A member 6(PDIA6)** | **10** | **10** | **36.4** | **440** | **8.1E-01** | **0.757** | **0.879** | **4.7E+09** |
| **Q7Z2Z2** | **elongation factor like GTPase 1(EFL1)** | **3** | **3** | **3.8** | **1120** | **8.0E-01** | **0.811** | **0.877** | **1.2E+08** |
| **P54652** | **heat shock protein family A (Hsp70) member 2(HSPA2)** | **16** | **9** | **32.7** | **639** | **7.8E-01** | **0.893** | **0.869** | **1.1E+09** |
| **Q9BQ67** | **glutamate rich WD repeat containing 1(GRWD1)** | **2** | **2** | **10.5** | **446** | **7.8E-01** | **0.815** | **0.867** | **1.5E+08** |
| **Q14137** | **BOP1 ribosomal biogenesis factor(BOP1)** | **1** | **1** | **3.4** | **746** | **7.7E-01** | **0.782** | **0.864** | **9.2E+07** |
| **P08238** | **heat shock protein 90 alpha family class B member 1(HSP90AB1)** | **21;7;2** | **12** | **36** | **724** | **7.6E-01** | **0.798** | **0.861** | **2.3E+10** |
| **P01116** | **KRAS proto-oncogene, GTPase(KRAS)** | **1;1;1** | **1** | **13.2** | **189** | **7.6E-01** | **0.857** | **0.859** | **1.1E+08** |
| **P12931** | **SRC proto-oncogene, non-receptor tyrosine kinase(SRC)** | **4;1;1;1;1** | **4** | **11.6** | **536** | **7.6E-01** | **0.828** | **0.859** | **2.8E+08** |
| **Q9UBT2** | **ubiquitin like modifier activating enzyme 2(UBA2)** | **6** | **6** | **14.4** | **640** | **7.6E-01** | **0.646** | **0.858** | **4.5E+08** |
| **P04075** | **aldolase, fructose-bisphosphate A(ALDOA)** | **11;1** | **10** | **42.6** | **364** | **7.5E-01** | **0.748** | **0.857** | **1.3E+10** |
| **P49915** | **guanine monophosphate synthase(GMPS)** | **11** | **11** | **29.1** | **693** | **7.5E-01** | **0.702** | **0.853** | **6.6E+08** |
| **O15067** | **phosphoribosylformylglycinamidine synthase(PFAS)** | **7** | **7** | **8.4** | **1338** | **7.3E-01** | **0.830** | **0.849** | **1.2E+09** |
| **P50991** | **chaperonin containing TCP1 subunit 4(CCT4)** | **10** | **10** | **23.7** | **539** | **7.3E-01** | **0.757** | **0.848** | **3.3E+09** |
| **P52597** | **heterogeneous nuclear ribonucleoprotein F(HNRNPF)** | **6** | **4** | **23.4** | **415** | **7.3E-01** | **0.731** | **0.848** | **1.7E+09** |
| **P37802** | **transgelin 2(TAGLN2)** | **6;1** | **6** | **41.7** | **199** | **7.2E-01** | **0.936** | **0.843** | **5.8E+09** |
| **P67809** | **Y-box binding protein 1(YBX1)** | **7** | **4** | **39.2** | **324** | **7.1E-01** | **0.759** | **0.840** | **8.8E+08** |
| **Q6NZI2** | **caveolae associated protein 1(CAVIN1)** | **7** | **7** | **25.4** | **390** | **7.0E-01** | **0.737** | **0.836** | **1.1E+09** |
| **Q8IVF2** | **AHNAK nucleoprotein 2(AHNAK2)** | **10** | **10** | **7.8** | **5795** | **7.0E-01** | **0.756** | **0.836** | **7.1E+08** |
| **Q15126** | **phosphomevalonate kinase(PMVK)** | **2** | **2** | **12.5** | **192** | **7.0E-01** | **0.795** | **0.833** | **9.5E+07** |
| **P06748** | **nucleophosmin 1(NPM1)** | **4** | **4** | **22.8** | **294** | **6.9E-01** | **0.745** | **0.832** | **3.8E+09** |
| **P31942** | **heterogeneous nuclear ribonucleoprotein H3(HNRNPH3)** | **3** | **3** | **11.3** | **346** | **6.9E-01** | **0.729** | **0.831** | **6.0E+08** |
| **Q13813** | **spectrin alpha, non-erythrocytic 1(SPTAN1)** | **48** | **48** | **26.9** | **2472** | **6.9E-01** | **0.777** | **0.829** | **7.7E+09** |
| **P50454** | **serpin family H member 1(SERPINH1)** | **8** | **8** | **23.4** | **418** | **6.8E-01** | **1.125** | **0.826** | **1.8E+09** |
| **P35637** | **FUS RNA binding protein(FUS)** | **4;1** | **4** | **9.9** | **526** | **6.7E-01** | **0.786** | **0.822** | **5.2E+08** |
| **Q13283** | **G3BP stress granule assembly factor 1(G3BP1)** | **7** | **7** | **24.5** | **466** | **6.7E-01** | **0.742** | **0.821** | **1.2E+09** |
| **Q15942** | **zyxin(ZYX)** | **1** | **1** | **3.7** | **572** | **6.6E-01** | **0.737** | **0.819** | **1.2E+08** |
| **O95433** | **activator of HSP90 ATPase activity 1(AHSA1)** | **6** | **6** | **30.8** | **338** | **6.6E-01** | **0.774** | **0.816** | **2.1E+09** |
| **Q15366** | **poly(rC) binding protein 2(PCBP2)** | **5** | **4** | **23.3** | **365** | **6.5E-01** | **0.686** | **0.814** | **4.6E+08** |
| **P04406** | **glyceraldehyde-3-phosphate dehydrogenase(GAPDH)** | **11** | **11** | **56.1** | **335** | **6.5E-01** | **0.801** | **0.812** | **2.7E+10** |
| **P10768** | **esterase D(ESD)** | **2** | **2** | **16** | **282** | **6.5E-01** | **0.729** | **0.811** | **2.1E+08** |
| **Q12797** | **aspartate beta-hydroxylase(ASPH)** | **11** | **11** | **21.6** | **758** | **6.4E-01** | **0.766** | **0.809** | **1.4E+09** |
| **P31939** | **5-aminoimidazole-4-carboxamide ribonucleotide formyltransferase/IMP cyclohydrolase(ATIC)** | **19** | **19** | **46.3** | **592** | **6.3E-01** | **0.753** | **0.807** | **2.6E+09** |
| **P13010** | **X-ray repair cross complementing 5(XRCC5)** | **11** | **11** | **28.3** | **732** | **6.3E-01** | **0.833** | **0.803** | **2.5E+09** |
| **P04632** | **calpain small subunit 1(CAPNS1)** | **5** | **5** | **47** | **268** | **6.2E-01** | **0.718** | **0.802** | **1.4E+09** |
| **P60228** | **eukaryotic translation initiation factor 3 subunit E(EIF3E)** | **7** | **7** | **22.2** | **445** | **6.2E-01** | **0.737** | **0.799** | **4.7E+08** |
| **Q86U42** | **poly(A) binding protein nuclear 1(PABPN1)** | **2** | **2** | **6.9** | **306** | **6.1E-01** | **0.787** | **0.796** | **4.3E+08** |
| **Q9H4A4** | **arginyl aminopeptidase(RNPEP)** | **6** | **6** | **13.7** | **650** | **6.1E-01** | **0.780** | **0.795** | **6.6E+08** |
| **P67775** | **protein phosphatase 2 catalytic subunit alpha(PPP2CA)** | **3;3** | **3** | **16.5** | **309** | **5.9E-01** | **0.743** | **0.789** | **4.6E+08** |
| **O75369** | **filamin B(FLNB)** | **54** | **51** | **31.6** | **2602** | **5.8E-01** | **0.718** | **0.783** | **1.1E+10** |
| **P16144** | **integrin subunit beta 4(ITGB4)** | **2** | **2** | **1.9** | **1822** | **5.7E-01** | **0.750** | **0.778** | **3.1E+07** |
| **Q9P2J5** | **leucyl-tRNA synthetase 1(LARS1)** | **10** | **10** | **14.2** | **1176** | **5.7E-01** | **0.749** | **0.778** | **9.3E+08** |
| **Q9UHD8** | **septin 9(SEPTIN9)** | **9** | **9** | **17.7** | **586** | **5.7E-01** | **0.715** | **0.778** | **1.5E+09** |
| **P49368** | **chaperonin containing TCP1 subunit 3(CCT3)** | **13** | **13** | **34.5** | **545** | **5.7E-01** | **0.688** | **0.777** | **2.9E+09** |
| **P39023** | **ribosomal protein L3(RPL3)** | **4** | **4** | **15.6** | **403** | **5.7E-01** | **0.746** | **0.777** | **1.5E+09** |
| **P07437** | **tubulin beta class I(TUBB)** | **15;3** | **4** | **49.3** | **444** | **5.5E-01** | **0.783** | **0.768** | **1.9E+10** |
| **P36776** | **lon peptidase 1, mitochondrial(LONP1)** | **4** | **4** | **9.9** | **959** | **5.4E-01** | **0.718** | **0.766** | **2.2E+08** |
| **P04792** | **heat shock protein family B (small) member 1(HSPB1)** | **8** | **8** | **54.1** | **205** | **5.3E-01** | **0.725** | **0.759** | **1.8E+09** |
| **Q14152** | **eukaryotic translation initiation factor 3 subunit A(EIF3A)** | **14** | **14** | **12.8** | **1382** | **5.2E-01** | **0.712** | **0.755** | **1.4E+09** |
| **O43707** | **actinin alpha 4(ACTN4)** | **37;1** | **26** | **51.9** | **911** | **5.0E-01** | **0.728** | **0.746** | **2.4E+10** |
| **P20290** | **basic transcription factor 3(BTF3)** | **6** | **6** | **48.1** | **206** | **5.0E-01** | **0.684** | **0.746** | **1.6E+09** |
| **P34897** | **serine hydroxymethyltransferase 2(SHMT2)** | **7** | **6** | **19.6** | **504** | **4.8E-01** | **0.708** | **0.737** | **1.0E+09** |
| **Q8TEX9** | **importin 4(IPO4)** | **3** | **3** | **4.1** | **1081** | **4.8E-01** | **0.708** | **0.736** | **9.6E+07** |
| **Q7Z6Z7** | **HECT, UBA and WWE domain containing E3 ubiquitin protein ligase 1(HUWE1)** | **14** | **14** | **5.9** | **4374** | **4.8E-01** | **0.651** | **0.733** | **4.9E+08** |
| **Q14204** | **dynein cytoplasmic 1 heavy chain 1(DYNC1H1)** | **43** | **43** | **14** | **4646** | **4.7E-01** | **0.697** | **0.732** | **3.8E+09** |
| **O60664** | **perilipin 3(PLIN3)** | **9** | **9** | **36.9** | **434** | **4.6E-01** | **0.633** | **0.724** | **5.9E+08** |
| **Q15233** | **non-POU domain containing octamer binding(NONO)** | **10** | **9** | **28.9** | **471** | **4.6E-01** | **0.648** | **0.723** | **8.8E+08** |
| **Q9BQG0** | **MYB binding protein 1a(MYBBP1A)** | **9** | **9** | **11.1** | **1328** | **4.4E-01** | **0.699** | **0.717** | **4.5E+08** |
| **P21912** | **succinate dehydrogenase complex iron sulfur subunit B(SDHB)** | **2** | **2** | **9.3** | **280** | **4.4E-01** | **0.657** | **0.716** | **3.0E+08** |
| **P14618** | **pyruvate kinase M1/2(PKM)** | **21;1** | **21** | **46.7** | **531** | **4.3E-01** | **0.652** | **0.710** | **1.8E+10** |
| **P53396** | **ATP citrate lyase(ACLY)** | **22** | **22** | **26** | **1101** | **4.3E-01** | **0.639** | **0.707** | **6.1E+09** |
| **P61803** | **defender against cell death 1(DAD1)** | **2** | **2** | **19.5** | **113** | **4.1E-01** | **0.674** | **0.699** | **2.5E+08** |
| **P11021** | **heat shock protein family A (Hsp70) member 5(HSPA5)** | **18** | **17** | **32.3** | **654** | **4.0E-01** | **0.639** | **0.692** | **3.7E+09** |
| **P23921** | **ribonucleotide reductase catalytic subunit M1(RRM1)** | **3** | **3** | **7.3** | **792** | **4.0E-01** | **0.795** | **0.691** | **2.2E+08** |
| **P26639** | **threonyl-tRNA synthetase 1(TARS1)** | **7** | **7** | **10.5** | **723** | **4.0E-01** | **0.646** | **0.691** | **6.0E+08** |
| **P16152** | **carbonyl reductase 1(CBR1)** | **7;2** | **7** | **39.7** | **277** | **3.9E-01** | **0.665** | **0.685** | **2.5E+09** |
| **O00151** | **PDZ and LIM domain 1(PDLIM1)** | **3** | **3** | **18.8** | **329** | **3.7E-01** | **0.669** | **0.677** | **3.5E+08** |
| **Q13509** | **tubulin beta 3 class III(TUBB3)** | **14** | **5** | **46.7** | **450** | **3.6E-01** | **0.609** | **0.670** | **1.7E+09** |
| **Q16719** | **kynureninase(KYNU)** | **9** | **9** | **29** | **465** | **3.4E-01** | **0.577** | **0.661** | **1.5E+09** |
| **O95373** | **importin 7(IPO7)** | **8** | **8** | **10.9** | **1038** | **3.3E-01** | **0.572** | **0.654** | **1.7E+09** |
| **Q63HN8** | **ring finger protein 213(RNF213)** | **11** | **11** | **3.4** | **5207** | **3.3E-01** | **0.641** | **0.651** | **3.3E+08** |
| **O43795** | **myosin IB(MYO1B)** | **8;2** | **8** | **10.7** | **1136** | **3.3E-01** | **0.569** | **0.649** | **2.9E+08** |
| **O14980** | **exportin 1(XPO1)** | **13** | **13** | **16.5** | **1071** | **3.3E-01** | **0.606** | **0.649** | **8.8E+08** |
| **P07099** | **epoxide hydrolase 1(EPHX1)** | **6** | **6** | **17.1** | **455** | **3.2E-01** | **0.607** | **0.644** | **3.7E+09** |
| **P50995** | **annexin A11(ANXA11)** | **8;1** | **8** | **19.6** | **505** | **3.1E-01** | **0.570** | **0.638** | **1.2E+09** |
| **P14923** | **junction plakoglobin(JUP)** | **6** | **6** | **13.3** | **745** | **3.1E-01** | **0.558** | **0.636** | **4.4E+08** |
| **P68371** | **tubulin beta 4B class IVb(TUBB4B)** | **14;4** | **0** | **46.5** | **445** | **3.0E-01** | **0.613** | **0.636** | **5.5E+09** |
| **P07900** | **heat shock protein 90 alpha family class A member 1(HSP90AA1)** | **20;5;4;1** | **12** | **34** | **732** | **2.8E-01** | **0.533** | **0.620** | **1.2E+10** |
| **P35232** | **prohibitin 1(PHB1)** | **9** | **9** | **44.5** | **272** | **2.7E-01** | **0.575** | **0.609** | **1.6E+09** |
| **O75643** | **small nuclear ribonucleoprotein U5 subunit 200(SNRNP200)** | **17** | **17** | **11.5** | **2136** | **2.4E-01** | **0.552** | **0.589** | **1.0E+09** |
| **Q7KZF4** | **staphylococcal nuclease and tudor domain containing 1(SND1)** | **14** | **14** | **23** | **910** | **2.3E-01** | **0.541** | **0.584** | **2.2E+09** |
| **P45974** | **ubiquitin specific peptidase 5(USP5)** | **9;1** | **9** | **20.9** | **858** | **1.9E-01** | **0.627** | **0.551** | **6.0E+08** |
| **Q9H9B4** | **sideroflexin 1(SFXN1)** | **6** | **6** | **24.8** | **322** | **1.9E-01** | **0.493** | **0.546** | **5.4E+08** |
| **Q6P5R6** | **ribosomal protein L22 like 1(RPL22L1)** | **1** | **1** | **19.7** | **122** | **1.8E-01** | **0.507** | **0.539** | **7.0E+07** |
| **Q9BVI4** | **nucleolar complex associated 4 homolog(NOC4L)** | **1** | **1** | **4.1** | **516** | **1.7E-01** | **0.520** | **0.529** | **1.3E+08** |
| **Q14444** | **cell cycle associated protein 1(CAPRIN1)** | **5** | **5** | **9** | **709** | **1.6E-01** | **0.536** | **0.518** | **7.2E+08** |
| **Q14684** | **ribosomal RNA processing 1B(RRP1B)** | **1** | **1** | **2.8** | **758** | **1.6E-01** | **0.486** | **0.515** | **7.2E+07** |
| **P02545** | **lamin A/C(LMNA)** | **26** | **26** | **44.4** | **664** | **1.5E-01** | **0.478** | **0.511** | **1.2E+10** |
| **P30086** | **phosphatidylethanolamine binding protein 1(PEBP1)** | **5** | **5** | **48.7** | **187** | **1.5E-01** | **0.462** | **0.506** | **1.7E+09** |
| **Q15365** | **poly(rC) binding protein 1(PCBP1)** | **6;1** | **5** | **30.9** | **356** | **1.5E-01** | **0.453** | **0.503** | **1.0E+09** |
| **Q15029** | **elongation factor Tu GTP binding domain containing 2(EFTUD2)** | **12** | **11** | **17.3** | **972** | **1.4E-01** | **0.481** | **0.491** | **1.4E+09** |
| **P00491** | **purine nucleoside phosphorylase(PNP)** | **4** | **4** | **24.9** | **289** | **1.3E-01** | **0.428** | **0.489** | **6.4E+08** |
| **P62258** | **tyrosine 3-monooxygenase/tryptophan 5-monooxygenase activation protein epsilon(YWHAE)** | **12** | **10** | **58** | **255** | **9.9E-02** | **0.420** | **0.442** | **7.3E+09** |
| **Q6P2Q9** | **pre-mRNA processing factor 8(PRPF8)** | **16** | **16** | **10.5** | **2335** | **8.8E-02** | **0.381** | **0.425** | **9.2E+08** |
| **P49588** | **alanyl-tRNA synthetase 1(AARS1)** | **12** | **12** | **20.5** | **968** | **8.3E-02** | **0.425** | **0.416** | **1.4E+09** |
| **Q13363** | **C-terminal binding protein 1(CTBP1)** | **2** | **2** | **6.6** | **440** | **8.1E-02** | **0.395** | **0.412** | **1.4E+08** |
| **P04083** | **annexin A1(ANXA1)** | **16** | **16** | **54.6** | **346** | **7.3E-02** | **0.370** | **0.397** | **1.5E+10** |
| **Q99497** | **Parkinsonism associated deglycase(PARK7)** | **4** | **4** | **29.6** | **189** | **5.9E-02** | **0.396** | **0.369** | **1.8E+09** |
| **P53004** | **biliverdin reductase A(BLVRA)** | **7** | **7** | **35.8** | **296** | **5.9E-02** | **0.349** | **0.368** | **1.0E+09** |
| **P07910** | **heterogeneous nuclear ribonucleoprotein C(HNRNPC)** | **6;3;3;3;3** | **6** | **28.4** | **306** | **5.8E-02** | **0.437** | **0.366** | **2.9E+09** |
| **P11142** | **heat shock protein family A (Hsp70) member 8(HSPA8)** | **22** | **15** | **45.2** | **646** | **5.0E-02** | **0.340** | **0.347** | **1.9E+10** |
| **Q06830** | **peroxiredoxin 1(PRDX1)** | **8;1** | **7** | **41.2** | **199** | **4.9E-02** | **0.368** | **0.344** | **3.5E+09** |
| **Q92841** | **DEAD-box helicase 17(DDX17)** | **9** | **5** | **15.2** | **729** | **4.6E-02** | **0.320** | **0.335** | **5.6E+08** |
| **Q15758** | **solute carrier family 1 member 5(SLC1A5)** | **6** | **6** | **14.8** | **541** | **4.5E-02** | **0.291** | **0.331** | **3.2E+08** |
| **Q5TZA2** | **ciliary rootlet coiled-coil, rootletin(CROCC)** | **1** | **1** | **0.4** | **2017** | **2.9E-02** | **0.273** | **0.279** | **1.4E+09** |
| **P51648** | **aldehyde dehydrogenase 3 family member A2(ALDH3A2)** | **8** | **7** | **19.4** | **485** | **2.9E-02** | **0.249** | **0.277** | **9.4E+08** |
| **P26038** | **moesin(MSN)** | **17** | **13** | **27** | **577** | **2.8E-02** | **0.256** | **0.270** | **3.9E+09** |
| **Q04637** | **eukaryotic translation initiation factor 4 gamma 1(EIF4G1)** | **8** | **8** | **6.5** | **1599** | **2.2E-02** | **0.234** | **0.246** | **1.1E+09** |
| **Q03135** | **caveolin 1(CAV1)** | **2** | **2** | **19.7** | **178** | **1.6E-02** | **0.206** | **0.205** | **8.2E+08** |
| **P22061** | **protein-L-isoaspartate (D-aspartate) O-methyltransferase(PCMT1)** | **3** | **3** | **26** | **227** | **1.5E-02** | **0.197** | **0.200** | **6.3E+08** |
| **P00352** | **aldehyde dehydrogenase 1 family member A1(ALDH1A1)** | **20** | **18** | **50.7** | **501** | **1.4E-02** | **0.160** | **0.194** | **3.3E+10** |
| **P32969** | **ribosomal protein L9(RPL9)** | **5** | **5** | **40.1** | **192** | **1.3E-02** | **0.170** | **0.184** | **1.9E+09** |
| **Q06323** | **proteasome activator subunit 1(PSME1)** | **8** | **8** | **36.1** | **249** | **1.3E-02** | **0.230** | **0.184** | **6.8E+08** |
| **Q96G03** | **phosphoglucomutase 2(PGM2)** | **1** | **1** | **2.9** | **612** | **1.3E-02** | **0.164** | **0.181** | **1.3E+08** |
| **P41252** | **isoleucyl-tRNA synthetase 1(IARS1)** | **15** | **15** | **15.3** | **1262** | **1.2E-02** | **0.166** | **0.172** | **1.2E+09** |
| **P35221** | **catenin alpha 1(CTNNA1)** | **4** | **4** | **7.7** | **906** | **1.2E-02** | **0.179** | **0.171** | **1.5E+09** |
| **P35241** | **radixin(RDX)** | **9** | **5** | **15.6** | **583** | **1.0E-02** | **0.141** | **0.156** | **1.0E+09** |
| **P43487** | **RAN binding protein 1(RANBP1)** | **2** | **2** | **10.9** | **201** | **8.5E-03** | **0.131** | **0.137** | **2.3E+08** |
| **P54136** | **arginyl-tRNA synthetase 1(RARS1)** | **11** | **11** | **19.4** | **660** | **8.4E-03** | **0.145** | **0.136** | **1.1E+09** |
| **P53597** | **succinate-CoA ligase GDP/ADP-forming subunit alpha(SUCLG1)** | **3** | **3** | **12.1** | **346** | **7.3E-03** | **0.120** | **0.121** | **3.7E+08** |
| **P00338** | **lactate dehydrogenase A(LDHA)** | **10** | **10** | **32.5** | **332** | **5.4E-03** | **0.085** | **0.090** | **9.8E+09** |
| **A5YKK6** | **CCR4-NOT transcription complex subunit 1(CNOT1)** | **7** | **7** | **4** | **2376** | **4.9E-03** | **0.090** | **0.081** | **5.9E+08** |
