# Supplementary material for: Ectopic ATP synthase stimulates the secretion of extracellular vesicles in cancer cells
Source: Commun Biol. 2023 Jun 15;6:642. doi: 10.1038/s42003-023-05008-5 (PMC10272197; doi:10.1038/s42003-023-05008-5)
Supplement: Supplementary file 4 — supplementary data 2 [file 42003_2023_5008_MOESM4_ESM.docx]

| **Supplementary Data 2. Detailed GO analysis data of 56 differential expressed proteins in A549** | | | | | |
| --- | --- | --- | --- | --- | --- |
| **Term** | **Count** | **%** | **PValue** | **Genes** | **Fold Enrichment** |
| Extracellular exosome | 28 | 50.0 | 2.78E-12 | P06733, P22061, P54136, P00390, Q15758, P12814, Q06323, Q9NR45, Q5TZA2, P50914, P41252, P35241, P78371, Q9Y230, P48643, P05198, P27824, P26038, P07339, Q06830, Q96G03, P11216, Q86VP6, P68104, P00338, P68366, P00352, P60842 | 4.48 |
| Extracellular vesicle | 28 | 50.0 | 7.61E-12 | P06733, P22061, P54136, P00390, Q15758, P12814, Q06323, Q9NR45, Q5TZA2, P50914, P41252, P35241, P78371, Q9Y230, P48643, P05198, P27824, P26038, P07339, Q06830, Q96G03, P11216, Q86VP6, P68104, P00338, P68366, P00352, P60842 | 4.29 |
| Extracellular organelle | 28 | 50.0 | 7.76E-12 | P06733, P22061, P54136, P00390, Q15758, P12814, Q06323, Q9NR45, Q5TZA2, P50914, P41252, P35241, P78371, Q9Y230, P48643, P05198, P27824, P26038, P07339, Q06830, Q96G03, P11216, Q86VP6, P68104, P00338, P68366, P00352, P60842 | 4.29 |
| Extracellular region part | 29 | 51.8 | 2.30E-07 | P06733, P22061, P54136, P00390, Q15758, P12814, Q06323, Q9NR45, A5YKK6, Q5TZA2, P50914, P41252, P35241, P78371, Q9Y230, P48643, P05198, P27824, P26038, P07339, Q06830, Q96G03, P11216, Q86VP6, P68104, P00338, P68366, P00352, P60842 | 2.62 |
| Intracellular part | 56 | 100.0 | 4.31E-06 | P06733, P22061, P54136, Q14847, P00390, P29692, P12814, P39656, A5YKK6, Q04637, P35221, P42704, P41252, Q92499, Q9Y230, P48643, P27824, P46821, O15371, Q2TB90, Q86VP6, P11216, P68104, P00338, Q92841, P46776, P52895, P32969, Q15758, Q06323, Q03135, P51648, Q9NR45, Q8NBQ5, Q5TZA2, P42166, P50914, P35241, P78371, Q9Y295, P05198, P26038, P09874, Q16891, P53597, P07339, P40939, Q06830, Q9Y6N5, Q96G03, P68400, P68366, P43487, P49748, P00352, P60842 | 1.25 |
| Intracellular | 56 | 100.0 | 4.42E-06 | P06733, P22061, P54136, Q14847, P00390, P29692, P12814, P39656, A5YKK6, Q04637, P35221, P42704, P41252, Q92499, Q9Y230, P48643, P27824, P46821, O15371, Q2TB90, Q86VP6, P11216, P68104, P00338, Q92841, P46776, P52895, P32969, Q15758, Q06323, Q03135, P51648, Q9NR45, Q8NBQ5, Q5TZA2, P42166, P50914, P35241, P78371, Q9Y295, P05198, P26038, P09874, Q16891, P53597, P07339, P40939, Q06830, Q9Y6N5, Q96G03, P68400, P68366, P43487, P49748, P00352, P60842 | 1.25 |
| Ribonucleoprotein complex | 13 | 23.2 | 1.65E-05 | O15371, P32969, A5YKK6, Q04637, P50914, P42704, Q92841, Q92499, Q9Y295, Q9Y230, P05198, P27824, P46776 | 4.56 |
| Non-membrane-bounded organelle | 32 | 57.1 | 1.71E-05 | P06733, Q14847, P32969, P29692, P12814, Q03135, Q8NBQ5, A5YKK6, Q04637, Q5TZA2, P42166, P50914, P35221, P42704, P35241, P78371, Q92499, Q9Y230, P48643, P05198, P27824, P46821, P26038, P09874, P40939, P68400, P68104, P68366, Q92841, P43487, P46776, P49748 | 1.98 |
| Membrane-bounded organelle | 52 | 92.9 | 3.24E-05 | P06733, P22061, P54136, P00390, P29692, P12814, P39656, A5YKK6, Q04637, P35221, P42704, P41252, Q92499, Q9Y230, P48643, P27824, Q2TB90, Q86VP6, P11216, P68104, P00338, Q92841, P46776, P32969, Q15758, Q06323, Q03135, P51648, Q9NR45, Q8NBQ5, Q5TZA2, P42166, P50914, P35241, P78371, Q9Y295, P05198, P26038, P09874, Q16891, P53597, P07339, P40939, Q06830, Q9Y6N5, Q96G03, P68400, P68366, P43487, P49748, P00352, P60842 | 1.33 |
| Protein complex | 22 | 39.3 | 9.50E-05 | O15371, P53597, P54136, P29692, Q06323, Q03135, A5YKK6, Q04637, P68400, P35221, P41252, P68104, P78371, Q92499, Q9Y230, P48643, P05198, P46821, P43487, Q16891, P09874, P60842 | 2.38 |
| Intracellular organelle | 50 | 89.3 | 1.04E-03 | P06733, P54136, Q14847, P00390, P29692, P12814, P39656, A5YKK6, Q04637, P35221, P42704, P41252, Q92499, Q9Y230, P48643, P27824, P46821, Q2TB90, Q86VP6, P11216, P68104, P00338, Q92841, P46776, P32969, Q15758, Q06323, Q03135, P51648, Q8NBQ5, Q5TZA2, P42166, P50914, P35241, P78371, Q9Y295, P05198, P26038, P09874, Q16891, P53597, P07339, P40939, Q06830, Q9Y6N5, Q96G03, P68400, P68366, P43487, P49748 | 1.26 |
| Cell-substrate junction | 7 | 12.5 | 1.19E-03 | P32969, P35221, Q14847, P35241, P12814, Q03135, P26038 | 5.76 |
| Intracellular organelle part | 42 | 75.0 | 1.51E-03 | P06733, P54136, Q14847, P32969, P00390, P29692, P12814, Q06323, Q03135, P51648, P39656, A5YKK6, Q5TZA2, P42166, P50914, P42704, P41252, P35241, P78371, Q92499, Q9Y295, Q9Y230, P48643, P27824, P46821, P09874, Q16891, P53597, P07339, P40939, Q2TB90, Q9Y6N5, Q96G03, P11216, Q86VP6, P68400, P68104, P68366, Q92841, P43487, P46776, P49748 | 1.38 |
| Organelle part | 42 | 75.0 | 3.14E-03 | P06733, P54136, Q14847, P32969, P00390, P29692, P12814, Q06323, Q03135, P51648, P39656, A5YKK6, Q5TZA2, P42166, P50914, P42704, P41252, P35241, P78371, Q92499, Q9Y295, Q9Y230, P48643, P27824, P46821, P09874, Q16891, P53597, P07339, P40939, Q2TB90, Q9Y6N5, Q96G03, P11216, Q86VP6, P68400, P68104, P68366, Q92841, P43487, P46776, P49748 | 1.34 |
| Anchoring junction | 7 | 12.5 | 5.08E-03 | P32969, P35221, Q14847, P35241, P12814, Q03135, P26038 | 4.30 |
| mitochondrial nucleoid | 3 | 5.4 | 6.91E-03 | P40939, P42704, P49748 | 23.64 |
| envelope | 10 | 17.9 | 8.41E-03 | P06733, P40939, P42166, P42704, Q2TB90, Q9Y6N5, P43487, P49748, Q16891, P09874 | 2.75 |
| cell part | 56 | 100.0 | 1.47E-02 | P06733, P22061, P54136, Q14847, P00390, P29692, P12814, P39656, A5YKK6, Q04637, P35221, P42704, P41252, Q92499, Q9Y230, P48643, P27824, P46821, O15371, Q2TB90, Q86VP6, P11216, P68104, P00338, Q92841, P46776, P52895, P32969, Q15758, Q06323, Q03135, P51648, Q9NR45, Q8NBQ5, Q5TZA2, P42166, P50914, P35241, P78371, Q9Y295, P05198, P26038, P09874, Q16891, P53597, P07339, P40939, Q06830, Q9Y6N5, Q96G03, P68400, P68366, P43487, P49748, P00352, P60842 | 1.08 |
| endomembrane system | 20 | 35.7 | 5.05E-02 | P06733, P07339, P29692, P12814, Q96G03, P11216, Q86VP6, P51648, Q03135, P39656, Q8NBQ5, P42166, P35221, P42704, P68104, P78371, P27824, P43487, P46776, P09874 | 1.49 |
| organelle lumen | 24 | 42.9 | 5.72E-02 | P53597, P07339, P54136, P40939, P00390, P29692, P12814, Q96G03, P11216, Q86VP6, Q06323, P68400, P42704, P41252, P68104, P78371, Q92841, Q92499, Q9Y295, Q9Y230, P27824, P49748, Q16891, P09874 | 1.39 |
| supramolecular polymer | 7 | 12.5 | 6.26E-02 | P06733, P42704, P12814, P68366, P78371, P48643, P46821 | 2.43 |
| whole membrane | 10 | 17.9 | 6.45E-02 | P06733, P07339, P35221, P42704, P68104, P51648, Q03135, P39656, A5YKK6, Q16891 | 1.93 |
| cell projection | 12 | 21.4 | 6.51E-02 | Q5TZA2, P35221, P68104, P35241, P12814, P27824, Q03135, P05198, P46821, P43487, P26038, P00352 | 1.76 |
| organelle membrane | 14 | 25.0 | 7.75E-02 | P06733, P07339, P40939, Q2TB90, Q9Y6N5, P51648, Q03135, P39656, A5YKK6, P42166, P42704, P68104, P49748, Q16891 | 1.60 |
| nuclear outer membrane | 2 | 3.6 | 7.99E-02 | P06733, P42704 | 23.64 |
