# Supplementary material for: Ectopic ATP synthase stimulates the secretion of extracellular vesicles in cancer cells
Source: Commun Biol. 2023 Jun 15;6:642. doi: 10.1038/s42003-023-05008-5 (PMC10272197; doi:10.1038/s42003-023-05008-5)
Supplement: Supplementary file 5 — supplementary data 3 [file 42003_2023_5008_MOESM5_ESM.docx]

| **Supplementary Data 3. Detailed proteomics data of 123 quantified proteins in L-EVs derived from A549** | | | | | | | | | |
| --- | --- | --- | --- | --- | --- | --- | --- | --- | --- |
| **Protein ID** | **Protein**  **Name (Gene Name)** | **Peptide**  **counts (all)** | **Unique**  **peptides** | **Sequence coverage [%]** | **Sequence**  **length** | **p-value** | **Ratio H/L** | **Ratio H/L normalized** | **Intensity** |
| **P30838** | **aldehyde dehydrogenase 3 family member A1(ALDH3A1)** | **7;1;1** | **6** | **20.3** | **453** | **1.3E-07** | **11.137** | **13.173** | **6.4E+09** |
| **P00352** | **aldehyde dehydrogenase 1 family member A1(ALDH1A1)** | **13** | **12** | **36.3** | **501** | **4.1E-06** | **11.693** | **11.704** | **1.1E+10** |
| **Q14974** | **karyopherin subunit beta 1(KPNB1)** | **10** | **10** | **16.1** | **876** | **9.8E-03** | **6.143** | **7.328** | **8.9E+08** |
| **P78527** | **protein kinase, DNA-activated, catalytic subunit(PRKDC)** | **25** | **25** | **8.8** | **4128** | **1.2E-02** | **6.541** | **7.164** | **2.0E+09** |
| **P0DMV9** | **heat shock protein family A (Hsp70) member 1B(HSPA1B)** | **9;9** | **5** | **20.9** | **641** | **1.2E-02** | **6.970** | **7.153** | **2.4E+09** |
| **P29692** | **eukaryotic translation elongation factor 1 delta(EEF1D)** | **4** | **4** | **21.7** | **281** | **2.2E-02** | **6.996** | **6.711** | **6.9E+08** |
| **Q14764** | **major vault protein(MVP)** | **17** | **17** | **28.2** | **893** | **2.3E-02** | **6.111** | **6.646** | **2.6E+09** |
| **P23396** | **ribosomal protein S3(RPS3)** | **7** | **7** | **36.2** | **243** | **3.4E-02** | **5.365** | **6.318** | **2.7E+09** |
| **P49327** | **fatty acid synthase(FASN)** | **35** | **35** | **22.2** | **2511** | **5.1E-02** | **5.664** | **5.952** | **5.8E+09** |
| **P42704** | **leucine rich pentatricopeptide repeat containing(LRPPRC)** | **12** | **12** | **12.2** | **1394** | **7.7E-02** | **4.926** | **5.558** | **5.1E+08** |
| **P13804** | **electron transfer flavoprotein subunit alpha(ETFA)** | **8** | **8** | **40.8** | **333** | **9.5E-02** | **4.145** | **5.351** | **6.3E+08** |
| **O75533** | **splicing factor 3b subunit 1(SF3B1)** | **4** | **4** | **5.2** | **1304** | **1.1E-01** | **4.791** | **5.177** | **1.8E+08** |
| **P63010** | **adaptor related protein complex 2 subunit beta 1(AP2B1)** | **4;1** | **4** | **6.7** | **937** | **1.7E-01** | **4.891** | **4.675** | **1.6E+08** |
| **P20290** | **basic transcription factor 3(BTF3)** | **5** | **5** | **46.1** | **206** | **1.9E-01** | **3.832** | **4.573** | **1.9E+09** |
| **P68400** | **casein kinase 2 alpha 1(CSNK2A1)** | **6;5** | **6** | **24** | **391** | **2.2E-01** | **3.650** | **4.385** | **9.0E+08** |
| **P06858** | **lipoprotein lipase(LPL)** | **1** | **1** | **3.6** | **475** | **2.2E-01** | **3.982** | **4.364** | **1.2E+08** |
| **Q16658** | **fascin actin-bundling protein 1(FSCN1)** | **1** | **1** | **3.2** | **493** | **2.3E-01** | **3.447** | **4.339** | **2.6E+08** |
| **P09525** | **annexin A4(ANXA4)** | **12** | **12** | **41.7** | **319** | **2.6E-01** | **2.888** | **4.170** | **4.5E+09** |
| **P08865** | **ribosomal protein SA(RPSA)** | **6** | **6** | **30.5** | **295** | **2.6E-01** | **3.524** | **4.167** | **2.5E+09** |
| **P31153** | **methionine adenosyltransferase 2A(MAT2A)** | **4;1** | **4** | **15.4** | **395** | **3.2E-01** | **3.696** | **3.898** | **7.7E+08** |
| **P26640** | **valyl-tRNA synthetase 1(VARS1)** | **6** | **6** | **6.7** | **1264** | **3.3E-01** | **3.207** | **3.858** | **3.6E+08** |
| **Q13085** | **acetyl-CoA carboxylase alpha(ACACA)** | **4** | **4** | **3.3** | **2346** | **3.4E-01** | **3.689** | **3.819** | **5.0E+07** |
| **P52895** | **aldo-keto reductase family 1 member C2(AKR1C2)** | **10** | **3** | **44.9** | **323** | **3.4E-01** | **3.631** | **3.804** | **7.2E+09** |
| **P26006** | **integrin subunit alpha 3(ITGA3)** | **6** | **6** | **7.9** | **1051** | **4.0E-01** | **3.393** | **3.557** | **1.1E+09** |
| **P04083** | **annexin A1(ANXA1)** | **10** | **10** | **37.6** | **346** | **4.4E-01** | **3.333** | **3.400** | **4.7E+09** |
| **P21980** | **transglutaminase 2(TGM2)** | **6** | **6** | **12.7** | **687** | **4.5E-01** | **3.136** | **3.362** | **1.0E+09** |
| **P51665** | **proteasome 26S subunit, non-ATPase 7(PSMD7)** | **1** | **1** | **7.4** | **324** | **4.6E-01** | **2.699** | **3.339** | **1.5E+08** |
| **Q92614** | **myosin XVIIIA(MYO18A)** | **1** | **1** | **1** | **2054** | **4.7E-01** | **3.018** | **3.296** | **7.4E+07** |
| **O00560** | **syndecan binding protein(SDCBP)** | **3** | **3** | **17.4** | **298** | **4.8E-01** | **3.232** | **3.274** | **9.3E+07** |
| **P07093** | **serpin family E member 2(SERPINE2)** | **2** | **2** | **7.5** | **398** | **4.9E-01** | **3.433** | **3.220** | **5.8E+07** |
| **Q15008** | **proteasome 26S subunit, non-ATPase 6(PSMD6)** | **4** | **4** | **13.4** | **389** | **5.0E-01** | **3.072** | **3.187** | **5.0E+08** |
| **P07437** | **tubulin beta class I(TUBB)** | **14;2** | **4** | **47.5** | **444** | **5.4E-01** | **2.916** | **3.063** | **1.3E+10** |
| **P50995** | **annexin A11(ANXA11)** | **8** | **8** | **19.8** | **505** | **5.4E-01** | **2.554** | **3.061** | **4.5E+08** |
| **P51148** | **RAB5C, member RAS oncogene family(RAB5C)** | **5;2** | **3** | **31** | **216** | **5.6E-01** | **3.057** | **2.997** | **4.7E+08** |
| **Q00610** | **clathrin heavy chain(CLTC)** | **36;8** | **36** | **32.1** | **1675** | **5.8E-01** | **2.503** | **2.919** | **9.6E+09** |
| **P22695** | **ubiquinol-cytochrome c reductase core protein 2(UQCRC2)** | **5** | **5** | **18.1** | **453** | **5.9E-01** | **2.776** | **2.903** | **9.6E+08** |
| **P15559** | **NAD(P)H quinone dehydrogenase 1(NQO1)** | **7** | **7** | **28.5** | **274** | **6.1E-01** | **2.554** | **2.836** | **7.0E+09** |
| **Q5T4S7** | **ubiquitin protein ligase E3 component n-recognin 4(UBR4)** | **12** | **12** | **4.6** | **5183** | **6.3E-01** | **2.611** | **2.777** | **3.1E+08** |
| **P13010** | **X-ray repair cross complementing 5(XRCC5)** | **5** | **5** | **13.3** | **732** | **6.7E-01** | **3.663** | **2.656** | **6.2E+08** |
| **Q9UIA9** | **exportin 7(XPO7)** | **3** | **3** | **4.3** | **1087** | **6.8E-01** | **2.503** | **2.634** | **1.1E+08** |
| **Q99832** | **chaperonin containing TCP1 subunit 7(CCT7)** | **7** | **7** | **19.5** | **543** | **7.1E-01** | **2.538** | **2.549** | **6.6E+08** |
| **P68371** | **tubulin beta 4B class IVb(TUBB4B)** | **13;3** | **0** | **44.7** | **445** | **7.3E-01** | **2.402** | **2.491** | **2.3E+09** |
| **P61163** | **actin related protein 1A(ACTR1A)** | **2** | **2** | **10.1** | **376** | **7.3E-01** | **2.077** | **2.475** | **1.1E+08** |
| **P31939** | **5-aminoimidazole-4-carboxamide ribonucleotide formyltransferase/IMP cyclohydrolase(ATIC)** | **15** | **15** | **38.5** | **592** | **7.5E-01** | **2.181** | **2.431** | **1.6E+09** |
| **Q7Z6Z7** | **HECT, UBA and WWE domain containing E3 ubiquitin protein ligase 1(HUWE1)** | **22** | **22** | **9.3** | **4374** | **7.8E-01** | **2.072** | **2.327** | **1.3E+09** |
| **Q9UJZ1** | **stomatin like 2(STOML2)** | **3** | **3** | **13.5** | **356** | **8.0E-01** | **2.290** | **2.288** | **2.0E+08** |
| **P22314** | **ubiquitin like modifier activating enzyme 1(UBA1)** | **10** | **10** | **14.9** | **1058** | **8.1E-01** | **1.941** | **2.244** | **6.0E+08** |
| **Q14204** | **dynein cytoplasmic 1 heavy chain 1(DYNC1H1)** | **30** | **30** | **10.6** | **4646** | **8.2E-01** | **2.009** | **2.223** | **1.8E+09** |
| **P31943** | **heterogeneous nuclear ribonucleoprotein H1(HNRNPH1)** | **3** | **0** | **11.1** | **449** | **8.3E-01** | **2.058** | **2.197** | **2.3E+08** |
| **O75390** | **citrate synthase(CS)** | **4** | **4** | **15** | **466** | **8.4E-01** | **1.939** | **2.171** | **3.8E+08** |
| **P78371** | **chaperonin containing TCP1 subunit 2(CCT2)** | **13** | **13** | **40** | **535** | **8.7E-01** | **2.044** | **2.099** | **2.1E+09** |
| **O00159** | **myosin IC(MYO1C)** | **8** | **8** | **11.4** | **1063** | **8.9E-01** | **1.702** | **2.021** | **2.9E+08** |
| **P52209** | **phosphogluconate dehydrogenase(PGD)** | **8** | **8** | **22.2** | **483** | **9.2E-01** | **1.874** | **1.960** | **7.1E+08** |
| **O95299** | **NADH:ubiquinone oxidoreductase subunit A10(NDUFA10)** | **1** | **1** | **6.2** | **355** | **9.2E-01** | **1.691** | **1.959** | **1.7E+08** |
| **P31949** | **S100 calcium binding protein A11(S100A11)** | **1** | **1** | **15.2** | **105** | **9.2E-01** | **1.773** | **1.938** | **5.0E+07** |
| **P25788** | **proteasome 20S subunit alpha 3(PSMA3)** | **2** | **2** | **10.2** | **255** | **9.3E-01** | **1.420** | **1.932** | **1.0E+08** |
| **P04844** | **ribophorin II(RPN2)** | **12** | **12** | **30.6** | **631** | **9.3E-01** | **1.586** | **1.917** | **7.1E+08** |
| **P16615** | **ATPase sarcoplasmic/endoplasmic reticulum Ca2+ transporting 2(ATP2A2)** | **6;3** | **6** | **8.7** | **1042** | **9.4E-01** | **1.898** | **1.887** | **2.1E+08** |
| **P35579** | **myosin heavy chain 9(MYH9)** | **21;2** | **17** | **15.6** | **1960** | **9.5E-01** | **1.430** | **1.880** | **4.6E+09** |
| **P50991** | **chaperonin containing TCP1 subunit 4(CCT4)** | **7** | **7** | **19.7** | **539** | **9.8E-01** | **1.857** | **1.800** | **1.9E+09** |
| **P56192** | **methionyl-tRNA synthetase 1(MARS1)** | **3** | **3** | **3.8** | **900** | **1.0E+00** | **2.139** | **1.737** | **2.9E+08** |
| **P46940** | **IQ motif containing GTPase activating protein 1(IQGAP1)** | **4** | **4** | **4.8** | **1657** | **1.0E+00** | **1.610** | **1.733** | **2.8E+08** |
| **Q9BQE3** | **tubulin alpha 1c(TUBA1C)** | **11;11;6;1** | **0** | **37.2** | **449** | **9.5E-01** | **1.810** | **1.663** | **1.6E+10** |
| **O00299** | **chloride intracellular channel 1(CLIC1)** | **4** | **4** | **28.6** | **241** | **9.2E-01** | **1.230** | **1.630** | **4.7E+08** |
| **P60709** | **actin beta(ACTB)** | **13;13;5** | **5** | **45.1** | **375** | **9.0E-01** | **1.620** | **1.602** | **4.3E+10** |
| **Q16181** | **septin 7(SEPTIN7)** | **2** | **2** | **8.5** | **437** | **9.0E-01** | **1.372** | **1.593** | **1.0E+08** |
| **P98160** | **heparan sulfate proteoglycan 2(HSPG2)** | **24** | **24** | **8.7** | **4391** | **8.9E-01** | **1.409** | **1.587** | **2.5E+09** |
| **P27105** | **stomatin(STOM)** | **3** | **3** | **17.7** | **288** | **8.9E-01** | **1.243** | **1.586** | **1.7E+08** |
| **P10809** | **heat shock protein family D (Hsp60) member 1(HSPD1)** | **14** | **14** | **38.7** | **573** | **8.8E-01** | **1.567** | **1.568** | **3.2E+09** |
| **P21333** | **filamin A(FLNA)** | **26;1** | **25** | **16.2** | **2647** | **8.4E-01** | **1.243** | **1.510** | **2.8E+09** |
| **O00116** | **alkylglycerone phosphate synthase(AGPS)** | **4** | **4** | **10** | **658** | **8.2E-01** | **1.259** | **1.485** | **2.2E+08** |
| **P30101** | **protein disulfide isomerase family A member 3(PDIA3)** | **8** | **8** | **25.1** | **505** | **8.1E-01** | **1.357** | **1.475** | **7.3E+08** |
| **Q9BSJ8** | **extended synaptotagmin 1(ESYT1)** | **4** | **4** | **6.2** | **1104** | **8.1E-01** | **1.522** | **1.472** | **1.6E+08** |
| **P55084** | **hydroxyacyl-CoA dehydrogenase trifunctional multienzyme complex subunit beta(HADHB)** | **2** | **2** | **7.8** | **474** | **7.9E-01** | **1.318** | **1.445** | **1.2E+08** |
| **Q86VP6** | **cullin associated and neddylation dissociated 1(CAND1)** | **15** | **15** | **17.5** | **1230** | **7.9E-01** | **1.370** | **1.438** | **9.2E+08** |
| **P17987** | **t-complex 1(TCP1)** | **9** | **9** | **27.3** | **556** | **7.7E-01** | **1.296** | **1.415** | **6.0E+08** |
| **O43169** | **cytochrome b5 type B(CYB5B)** | **3** | **3** | **36.7** | **150** | **7.7E-01** | **1.317** | **1.410** | **1.1E+09** |
| **P04843** | **ribophorin I(RPN1)** | **12** | **12** | **32.6** | **607** | **7.6E-01** | **1.254** | **1.394** | **9.0E+08** |
| **P39656** | **dolichyl-diphosphooligosaccharide--protein glycosyltransferase non-catalytic subunit(DDOST)** | **7** | **7** | **27.9** | **456** | **7.4E-01** | **1.492** | **1.363** | **4.0E+08** |
| **Q15149** | **plectin(PLEC)** | **35;3** | **35** | **11** | **4684** | **7.3E-01** | **1.329** | **1.361** | **2.6E+09** |
| **Q96TA1** | **niban apoptosis regulator 2(NIBAN2)** | **3** | **3** | **7.9** | **746** | **7.2E-01** | **1.308** | **1.343** | **5.0E+07** |
| **P06576** | **ATP synthase F1 subunit beta(ATP5F1B)** | **17** | **17** | **59.2** | **529** | **7.2E-01** | **1.118** | **1.342** | **4.6E+09** |
| **P12429** | **annexin A3(ANXA3)** | **3** | **3** | **12.7** | **323** | **6.7E-01** | **1.091** | **1.274** | **2.2E+08** |
| **P29401** | **transketolase(TKT)** | **5** | **5** | **17.8** | **623** | **6.7E-01** | **1.045** | **1.266** | **4.0E+08** |
| **P08195** | **solute carrier family 3 member 2(SLC3A2)** | **13** | **13** | **28.1** | **630** | **6.6E-01** | **1.345** | **1.254** | **2.9E+09** |
| **Q3B7T3** | **brain expressed associated with NEDD4 1(BEAN1)** | **1** | **1** | **3.9** | **259** | **6.1E-01** | **1.173** | **1.168** | **2.6E+08** |
| **P40227** | **chaperonin containing TCP1 subunit 6A(CCT6A)** | **8;1** | **8** | **21.5** | **531** | **5.9E-01** | **1.192** | **1.146** | **5.7E+08** |
| **P31930** | **ubiquinol-cytochrome c reductase core protein 1(UQCRC1)** | **5** | **5** | **17.9** | **480** | **5.8E-01** | **1.028** | **1.126** | **4.6E+08** |
| **Q9HDC9** | **adipocyte plasma membrane associated protein(APMAP)** | **2** | **2** | **9.4** | **416** | **5.6E-01** | **0.957** | **1.093** | **3.0E+08** |
| **P05783** | **keratin 18(KRT18)** | **22;3** | **14** | **60** | **430** | **5.1E-01** | **0.998** | **1.021** | **3.0E+10** |
| **P39060** | **collagen type XVIII alpha 1 chain(COL18A1)** | **9** | **9** | **9.9** | **1754** | **5.1E-01** | **1.086** | **1.009** | **2.1E+09** |
| **O60503** | **adenylate cyclase 9(ADCY9)** | **1** | **1** | **1.9** | **1353** | **5.0E-01** | **1.037** | **1.003** | **6.5E+08** |
| **P62304** | **small nuclear ribonucleoprotein polypeptide E(SNRPE)** | **1** | **1** | **12** | **92** | **4.9E-01** | **0.796** | **0.978** | **1.2E+08** |
| **P02751** | **fibronectin 1(FN1)** | **39** | **39** | **27.9** | **2477** | **4.9E-01** | **1.025** | **0.977** | **3.4E+10** |
| **Q9Y490** | **talin 1(TLN1)** | **30;3** | **30** | **18.6** | **2541** | **4.7E-01** | **0.753** | **0.945** | **1.6E+09** |
| **P61204** | **ADP ribosylation factor 3(ARF3)** | **8;8;5** | **4** | **54.7** | **181** | **4.6E-01** | **0.887** | **0.933** | **2.9E+09** |
| **P18206** | **vinculin(VCL)** | **5** | **5** | **7.3** | **1134** | **4.4E-01** | **0.717** | **0.883** | **2.3E+08** |
| **P25705** | **ATP synthase F1 subunit alpha(ATP5F1A)** | **11** | **11** | **28.2** | **553** | **4.3E-01** | **0.795** | **0.863** | **3.2E+09** |
| **P0C0L4** | **complement C4A (Rodgers blood group)(C4A)** | **3;2** | **1** | **2.5** | **1744** | **3.9E-01** | **0.676** | **0.786** | **4.1E+08** |
| **P10301** | **RAS related(RRAS)** | **3;1** | **3** | **22** | **218** | **3.8E-01** | **0.747** | **0.779** | **2.5E+08** |
| **Q96QK1** | **VPS35 retromer complex component(VPS35)** | **5** | **5** | **8.8** | **796** | **3.3E-01** | **0.684** | **0.675** | **3.8E+08** |
| **P40939** | **hydroxyacyl-CoA dehydrogenase trifunctional multienzyme complex subunit alpha(HADHA)** | **10** | **10** | **22** | **763** | **3.2E-01** | **0.596** | **0.647** | **5.4E+08** |
| **P62191** | **proteasome 26S subunit, ATPase 1(PSMC1)** | **4** | **4** | **17.3** | **440** | **3.2E-01** | **0.582** | **0.640** | **2.6E+08** |
| **P22626** | **heterogeneous nuclear ribonucleoprotein A2/B1(HNRNPA2B1)** | **4** | **4** | **15.9** | **353** | **3.1E-01** | **0.592** | **0.615** | **1.3E+09** |
| **P21399** | **aconitase 1(ACO1)** | **5** | **5** | **10.2** | **889** | **3.0E-01** | **0.595** | **0.590** | **4.4E+08** |
| **P06733** | **enolase 1(ENO1)** | **9** | **8** | **29.3** | **434** | **2.7E-01** | **0.581** | **0.536** | **1.8E+09** |
| **P01008** | **serpin family C member 1(SERPINC1)** | **4** | **1** | **11** | **464** | **2.5E-01** | **0.639** | **0.475** | **9.5E+07** |
| **P08559** | **pyruvate dehydrogenase E1 subunit alpha 1(PDHA1)** | **5;2** | **5** | **14.9** | **390** | **2.5E-01** | **0.459** | **0.464** | **3.5E+08** |
| **Q99829** | **copine 1(CPNE1)** | **2** | **2** | **5.8** | **537** | **2.4E-01** | **0.424** | **0.455** | **5.1E+07** |
| **Q07065** | **cytoskeleton associated protein 4(CKAP4)** | **4** | **4** | **10.1** | **602** | **2.4E-01** | **0.350** | **0.455** | **2.2E+08** |
| **O60763** | **USO1 vesicle transport factor(USO1)** | **8** | **8** | **13.4** | **962** | **2.3E-01** | **0.397** | **0.422** | **4.2E+08** |
| **Q9NZN3** | **EH domain containing 3(EHD3)** | **8;2** | **3** | **24.7** | **535** | **2.1E-01** | **0.356** | **0.377** | **1.6E+09** |
| **Q5T447** | **HECT domain E3 ubiquitin protein ligase 3(HECTD3)** | **3** | **3** | **5.3** | **861** | **2.1E-01** | **0.474** | **0.356** | **2.6E+08** |
| **P12270** | **translocated promoter region, nuclear basket protein(TPR)** | **1** | **1** | **0.8** | **2363** | **2.0E-01** | **0.315** | **0.331** | **8.7E+07** |
| **P07195** | **lactate dehydrogenase B(LDHB)** | **8** | **8** | **27.8** | **334** | **1.9E-01** | **0.285** | **0.298** | **1.3E+09** |
| **Q15907** | **RAB11B, member RAS oncogene family(RAB11B)** | **5;5;1** | **5** | **24.3** | **218** | **1.9E-01** | **0.263** | **0.289** | **1.3E+09** |
| **P06744** | **glucose-6-phosphate isomerase(GPI)** | **3;1** | **3** | **8.2** | **558** | **1.6E-01** | **0.189** | **0.214** | **6.7E+08** |
| **Q2TB90** | **hexokinase domain containing 1(HKDC1)** | **1** | **1** | **1** | **917** | **1.6E-01** | **0.152** | **0.192** | **2.2E+08** |
| **O43175** | **phosphoglycerate dehydrogenase(PHGDH)** | **3** | **3** | **6.4** | **533** | **1.5E-01** | **0.158** | **0.178** | **1.7E+09** |
| **P05141** | **solute carrier family 25 member 5(SLC25A5)** | **7;2** | **3** | **24.2** | **298** | **1.5E-01** | **0.168** | **0.157** | **1.4E+09** |
| **Q99880** | **H2B clustered histone 13(H2BC13)** | **2;2;2;2;2;2;2;2;2** | **1** | **19** | **126** | **1.2E-01** | **0.048** | **0.052** | **7.7E+09** |
| **P00742** | **coagulation factor X(F10)** | **4** | **4** | **8.2** | **488** | **1.2E-01** | **0.017** | **0.017** | **1.8E+10** |
