# Supplementary material for: Ectopic ATP synthase stimulates the secretion of extracellular vesicles in cancer cells
Source: Commun Biol. 2023 Jun 15;6:642. doi: 10.1038/s42003-023-05008-5 (PMC10272197; doi:10.1038/s42003-023-05008-5)
Supplement: Supplementary file 6 — supplementary data 4 [file 42003_2023_5008_MOESM6_ESM.docx]

| **Supplementary Data 4. Detailed proteomics data of 84 quantified proteins in S-EVs derived from A549** | | | | | | | | | |
| --- | --- | --- | --- | --- | --- | --- | --- | --- | --- |
| **Protein ID** | **Protein**  **Name (Gene Name)** | **Peptide**  **counts (all)** | **Unique**  **peptides** | **Sequence coverage [%]** | **Sequence**  **length** | **p-value** | **Ratio H/L** | **Ratio H/L normalized** | **Intensity** |
| **P14618** | **pyruvate kinase M1/2(PKM)** | **14** | **14** | **35.4** | **531** | **5.5E-38** | **18.921** | **17.643** | **8.2E+09** |
| **P13639** | **eukaryotic translation elongation factor 2(EEF2)** | **13** | **12** | **20.3** | **858** | **2.9E-14** | **8.579** | **10.824** | **1.1E+09** |
| **P17066** | **heat shock protein family A (Hsp70) member 6(HSPA6)** | **5;4** | **1** | **10** | **643** | **2.9E-13** | **10.876** | **10.429** | **8.4E+08** |
| **P25705** | **ATP synthase F1 subunit alpha(ATP5F1A)** | **5** | **5** | **13.4** | **553** | **4.9E-11** | **8.882** | **9.495** | **9.1E+08** |
| **P04075** | **aldolase, fructose-bisphosphate A(ALDOA)** | **7** | **7** | **30.2** | **364** | **2.5E-09** | **9.095** | **8.703** | **2.9E+09** |
| **P37802** | **transgelin 2(TAGLN2)** | **7;1** | **7** | **47.2** | **199** | **2.5E-06** | **7.259** | **7.085** | **2.3E+09** |
| **P63261** | **actin gamma 1(ACTG1)** | **12;12;5;2** | **5** | **42.9** | **375** | **1.1E-05** | **7.341** | **6.688** | **2.5E+10** |
| **P30838** | **aldehyde dehydrogenase 3 family member A1(ALDH3A1)** | **11;1;1** | **10** | **29.8** | **453** | **5.1E-05** | **6.871** | **6.241** | **4.8E+09** |
| **P33176** | **kinesin family member 5B(KIF5B)** | **1** | **1** | **1.1** | **963** | **1.0E-03** | **5.188** | **5.244** | **7.6E+08** |
| **P00488** | **coagulation factor XIII A chain(F13A1)** | **2** | **2** | **4** | **732** | **1.4E-03** | **5.734** | **5.142** | **1.2E+08** |
| **P11413** | **glucose-6-phosphate dehydrogenase(G6PD)** | **14** | **14** | **38.8** | **515** | **2.7E-02** | **4.102** | **3.870** | **8.4E+09** |
| **Q15582** | **transforming growth factor beta induced(TGFBI)** | **10** | **10** | **22.1** | **683** | **3.0E-02** | **3.803** | **3.812** | **1.3E+09** |
| **P35241** | **radixin(RDX)** | **5** | **3** | **10.8** | **583** | **8.6E-02** | **3.432** | **3.225** | **1.9E+08** |
| **Q14764** | **major vault protein(MVP)** | **13** | **13** | **21.1** | **893** | **1.2E-01** | **3.124** | **3.041** | **1.2E+09** |
| **P98160** | **heparan sulfate proteoglycan 2(HSPG2)** | **22** | **22** | **8.4** | **4391** | **1.2E-01** | **3.209** | **3.023** | **2.0E+09** |
| **P68363** | **tubulin alpha 1b(TUBA1B)** | **12;11;11;7;7;6;6;1** | **4** | **41.7** | **451** | **1.9E-01** | **2.656** | **2.694** | **8.7E+09** |
| **Q07020** | **ribosomal protein L18(RPL18)** | **2** | **2** | **13.8** | **188** | **2.0E-01** | **2.644** | **2.658** | **2.8E+08** |
| **P27708** | **carbamoyl-phosphate synthetase 2, aspartate transcarbamylase, and dihydroorotase(CAD)** | **23** | **23** | **17.2** | **2225** | **3.0E-01** | **2.638** | **2.338** | **2.3E+09** |
| **P12956** | **X-ray repair cross complementing 6(XRCC6)** | **9** | **9** | **23.2** | **609** | **3.2E-01** | **2.424** | **2.300** | **6.2E+08** |
| **P46776** | **ribosomal protein L27a(RPL27A)** | **2** | **2** | **14.2** | **148** | **3.2E-01** | **2.649** | **2.291** | **4.1E+08** |
| **P07195** | **lactate dehydrogenase B(LDHB)** | **8** | **8** | **28.7** | **334** | **3.7E-01** | **2.205** | **2.167** | **2.5E+09** |
| **Q14152** | **eukaryotic translation initiation factor 3 subunit A(EIF3A)** | **3** | **3** | **3.4** | **1382** | **4.0E-01** | **2.041** | **2.099** | **2.6E+08** |
| **P11216** | **glycogen phosphorylase B(PYGB)** | **12;2** | **10** | **18.4** | **843** | **4.0E-01** | **2.345** | **2.094** | **1.1E+09** |
| **P28074** | **proteasome 20S subunit beta 5(PSMB5)** | **5** | **5** | **19.4** | **263** | **4.2E-01** | **2.269** | **2.054** | **1.2E+09** |
| **Q14697** | **glucosidase II alpha subunit(GANAB)** | **8** | **8** | **11.7** | **944** | **4.7E-01** | **1.893** | **1.945** | **1.2E+09** |
| **P07437** | **tubulin beta class I(TUBB)** | **14;2** | **4** | **47.5** | **444** | **5.5E-01** | **1.839** | **1.780** | **9.1E+09** |
| **P27348** | **tyrosine 3-monooxygenase/tryptophan 5-monooxygenase activation protein theta(YWHAQ)** | **6** | **4** | **33.5** | **245** | **6.3E-01** | **1.322** | **1.629** | **1.8E+09** |
| **P49327** | **fatty acid synthase(FASN)** | **38** | **38** | **24.6** | **2511** | **6.9E-01** | **1.552** | **1.529** | **6.1E+09** |
| **Q07954** | **LDL receptor related protein 1(LRP1)** | **14** | **14** | **4.2** | **4544** | **6.9E-01** | **1.444** | **1.526** | **1.1E+09** |
| **P0DMV9** | **heat shock protein family A (Hsp70) member 1B(HSPA1B)** | **11;11** | **5** | **23.1** | **641** | **7.5E-01** | **1.341** | **1.416** | **2.4E+09** |
| **P06744** | **glucose-6-phosphate isomerase(GPI)** | **8** | **4** | **18.6** | **558** | **7.8E-01** | **1.239** | **1.373** | **1.9E+09** |
| **P08238** | **heat shock protein 90 alpha family class B member 1(HSP90AB1)** | **14;3** | **8** | **21.8** | **724** | **7.8E-01** | **1.280** | **1.362** | **1.1E+10** |
| **P21333** | **filamin A(FLNA)** | **33** | **32** | **20.7** | **2647** | **7.9E-01** | **1.281** | **1.346** | **5.6E+09** |
| **P62333** | **proteasome 26S subunit, ATPase 6(PSMC6)** | **3** | **3** | **11.8** | **389** | **8.0E-01** | **1.254** | **1.344** | **3.1E+08** |
| **P60900** | **proteasome 20S subunit alpha 6(PSMA6)** | **5** | **5** | **21.5** | **246** | **8.2E-01** | **1.080** | **1.308** | **2.0E+09** |
| **O60664** | **perilipin 3(PLIN3)** | **1** | **1** | **3.2** | **434** | **8.3E-01** | **1.224** | **1.286** | **1.5E+08** |
| **A6NHL2** | **tubulin alpha like 3(TUBAL3)** | **1** | **1** | **3.8** | **446** | **8.4E-01** | **1.222** | **1.275** | **8.3E+08** |
| **P62277** | **ribosomal protein S13(RPS13)** | **2** | **2** | **16.6** | **151** | **8.6E-01** | **1.238** | **1.232** | **9.6E+08** |
| **Q15365** | **poly(rC) binding protein 1(PCBP1)** | **2;1** | **1** | **12.1** | **356** | **8.8E-01** | **1.172** | **1.203** | **3.7E+08** |
| **P13804** | **electron transfer flavoprotein subunit alpha(ETFA)** | **3** | **3** | **12.9** | **333** | **8.8E-01** | **1.119** | **1.202** | **9.6E+07** |
| **O43708** | **glutathione S-transferase zeta 1(GSTZ1)** | **1** | **1** | **9.3** | **216** | **9.0E-01** | **1.143** | **1.176** | **1.8E+07** |
| **Q13200** | **proteasome 26S subunit ubiquitin receptor, non-ATPase 2(PSMD2)** | **4** | **4** | **6.6** | **908** | **9.2E-01** | **1.111** | **1.138** | **7.7E+08** |
| **P48643** | **chaperonin containing TCP1 subunit 5(CCT5)** | **3** | **3** | **7.9** | **541** | **9.3E-01** | **1.067** | **1.121** | **1.3E+08** |
| **P55072** | **valosin containing protein(VCP)** | **26** | **26** | **44.4** | **806** | **9.3E-01** | **1.220** | **1.118** | **1.2E+10** |
| **O75369** | **filamin B(FLNB)** | **15;2** | **14** | **9.1** | **2602** | **9.4E-01** | **1.061** | **1.106** | **2.1E+09** |
| **P07900** | **heat shock protein 90 alpha family class A member 1(HSP90AA1)** | **14;2;1;1;1** | **9** | **25.7** | **732** | **9.4E-01** | **1.103** | **1.099** | **8.6E+09** |
| **P67809** | **Y-box binding protein 1(YBX1)** | **3;1** | **2** | **17.6** | **324** | **9.5E-01** | **1.050** | **1.089** | **1.0E+09** |
| **P04632** | **calpain small subunit 1(CAPNS1)** | **3** | **3** | **19.4** | **268** | **9.5E-01** | **1.023** | **1.088** | **2.2E+08** |
| **Q6P2Q9** | **pre-mRNA processing factor 8(PRPF8)** | **3** | **3** | **2.1** | **2335** | **9.5E-01** | **1.043** | **1.083** | **1.8E+08** |
| **P67775** | **protein phosphatase 2 catalytic subunit alpha(PPP2CA)** | **1;1** | **1** | **8.1** | **309** | **9.6E-01** | **1.031** | **1.081** | **1.2E+08** |
| **P78347** | **general transcription factor IIi(GTF2I)** | **2** | **2** | **3.9** | **998** | **9.6E-01** | **0.988** | **1.072** | **6.0E+07** |
| **P08670** | **vimentin(VIM)** | **14;3;1;1;1;1** | **13** | **35.6** | **466** | **9.7E-01** | **0.976** | **1.053** | **4.9E+09** |
| **P00352** | **aldehyde dehydrogenase 1 family member A1(ALDH1A1)** | **16** | **15** | **40.3** | **501** | **9.8E-01** | **1.028** | **1.044** | **2.0E+10** |
| **O60884** | **DnaJ heat shock protein family (Hsp40) member A2(DNAJA2)** | **2** | **2** | **14.1** | **412** | **9.8E-01** | **1.004** | **1.041** | **1.4E+08** |
| **P39060** | **collagen type XVIII alpha 1 chain(COL18A1)** | **3** | **3** | **4** | **1754** | **9.8E-01** | **0.991** | **1.040** | **2.2E+08** |
| **P0C0L5** | **complement C4B (Chido blood group)(C4B)** | **4;4** | **1** | **3.2** | **1744** | **1.0E+00** | **0.999** | **1.009** | **7.1E+08** |
| **P35579** | **myosin heavy chain 9(MYH9)** | **28;4;3;1;1** | **28** | **19.3** | **1960** | **9.7E-01** | **0.967** | **0.999** | **6.8E+09** |
| **P14625** | **heat shock protein 90 beta family member 1(HSP90B1)** | **16;2** | **16** | **25.2** | **803** | **9.4E-01** | **0.947** | **0.991** | **4.4E+09** |
| **P07814** | **glutamyl-prolyl-tRNA synthetase 1(EPRS1)** | **6** | **6** | **6.6** | **1512** | **9.3E-01** | **0.995** | **0.987** | **2.4E+08** |
| **Q13813** | **spectrin alpha, non-erythrocytic 1(SPTAN1)** | **12** | **12** | **7.2** | **2472** | **9.3E-01** | **0.964** | **0.985** | **7.4E+08** |
| **P68371** | **tubulin beta 4B class IVb(TUBB4B)** | **13;3** | **0** | **44.7** | **445** | **9.1E-01** | **0.956** | **0.979** | **2.2E+09** |
| **P07099** | **epoxide hydrolase 1(EPHX1)** | **4** | **4** | **13.4** | **455** | **8.7E-01** | **0.932** | **0.967** | **5.0E+08** |
| **Q08211** | **DExH-box helicase 9(DHX9)** | **5** | **5** | **6.6** | **1270** | **8.1E-01** | **0.900** | **0.948** | **7.6E+08** |
| **P31943** | **heterogeneous nuclear ribonucleoprotein H1(HNRNPH1)** | **5;3;2** | **5** | **17.8** | **449** | **8.0E-01** | **0.887** | **0.942** | **7.2E+08** |
| **P46379** | **BAG cochaperone 6(BAG6)** | **1** | **1** | **2.7** | **1132** | **7.8E-01** | **0.884** | **0.937** | **5.4E+07** |
| **Q00610** | **clathrin heavy chain(CLTC)** | **33;6** | **33** | **29** | **1675** | **7.8E-01** | **0.891** | **0.936** | **6.4E+09** |
| **O00429** | **dynamin 1 like(DNM1L)** | **1** | **1** | **2.9** | **736** | **7.8E-01** | **0.950** | **0.936** | **4.7E+07** |
| **P30043** | **biliverdin reductase B(BLVRB)** | **3** | **3** | **26.2** | **206** | **7.8E-01** | **0.884** | **0.935** | **1.8E+08** |
| **Q15149** | **plectin(PLEC)** | **24;3** | **24** | **7.4** | **4684** | **7.7E-01** | **0.906** | **0.934** | **3.2E+09** |
| **P10809** | **heat shock protein family D (Hsp60) member 1(HSPD1)** | **9** | **9** | **27.6** | **573** | **7.6E-01** | **0.908** | **0.930** | **3.9E+09** |
| **O60701** | **UDP-glucose 6-dehydrogenase(UGDH)** | **9** | **9** | **29.8** | **494** | **7.0E-01** | **0.866** | **0.908** | **3.9E+09** |
| **P20700** | **lamin B1(LMNB1)** | **3** | **3** | **10.1** | **586** | **6.8E-01** | **0.869** | **0.900** | **4.8E+08** |
| **P02751** | **fibronectin 1(FN1)** | **38** | **38** | **26.5** | **2477** | **6.6E-01** | **0.901** | **0.894** | **3.1E+10** |
| **P09211** | **glutathione S-transferase pi 1(GSTP1)** | **2** | **2** | **14.3** | **210** | **6.6E-01** | **0.869** | **0.893** | **2.0E+09** |
| **O43707** | **actinin alpha 4(ACTN4)** | **23;5;2;1** | **14** | **33.3** | **911** | **6.4E-01** | **0.856** | **0.887** | **8.5E+09** |
| **Q99880** | **H2B clustered histone 13(H2BC13)** | **4;4;4;4;4;4;4;4;4;3;3;3;3;3;2;1** | **4** | **34.9** | **126** | **6.3E-01** | **0.870** | **0.885** | **1.2E+10** |
| **Q12906** | **interleukin enhancer binding factor 3(ILF3)** | **1** | **1** | **2.6** | **894** | **6.3E-01** | **0.863** | **0.884** | **3.2E+08** |
| **P31946** | **tyrosine 3-monooxygenase/tryptophan 5-monooxygenase activation protein beta(YWHAB)** | **5** | **3** | **25.6** | **246** | **5.9E-01** | **0.861** | **0.870** | **1.4E+08** |
| **Q16181** | **septin 7(SEPTIN7)** | **2** | **2** | **8.5** | **437** | **5.9E-01** | **0.844** | **0.869** | **9.9E+07** |
| **P00966** | **argininosuccinate synthase 1(ASS1)** | **2** | **2** | **10.9** | **412** | **5.2E-01** | **0.834** | **0.842** | **4.8E+07** |
| **O95433** | **activator of HSP90 ATPase activity 1(AHSA1)** | **1** | **1** | **6.5** | **338** | **4.9E-01** | **0.787** | **0.831** | **2.4E+08** |
| **P49902** | **5'-nucleotidase, cytosolic II(NT5C2)** | **3** | **3** | **9.6** | **561** | **4.8E-01** | **0.769** | **0.825** | **2.5E+08** |
| **O43169** | **cytochrome b5 type B(CYB5B)** | **3** | **3** | **36.7** | **150** | **4.8E-01** | **0.798** | **0.824** | **1.6E+09** |
