# Supplementary material for: Ectopic ATP synthase stimulates the secretion of extracellular vesicles in cancer cells
Source: Commun Biol. 2023 Jun 15;6:642. doi: 10.1038/s42003-023-05008-5 (PMC10272197; doi:10.1038/s42003-023-05008-5)
Supplement: Supplementary file 7 — supplementary data 5 [file 42003_2023_5008_MOESM7_ESM.docx]

**Supplementary Data 5. Transmembrane prediction of Fyn-T**

| **Amino acid** | **Prediction** |
| --- | --- |
| 1-224 | Outside |
| 225-234 | TMH (half) |
| 235-364 | Outside |
| 365-379 | TMH |
| 380-448 | Inside |
| 449-458 | TMH (half) |
| 459-51 | Inside |
| 513-519 | TMH (half) |
| 520-537 | Inside |
