# Supplementary material for: Ectopic ATP synthase stimulates the secretion of extracellular vesicles in cancer cells
Source: Commun Biol. 2023 Jun 15;6:642. doi: 10.1038/s42003-023-05008-5 (PMC10272197; doi:10.1038/s42003-023-05008-5)
Supplement: Supplementary file 8 — supplementary data 6 [file 42003_2023_5008_MOESM8_ESM.docx]

| **Supplementary Data 6. Key resource table** | | |
| --- | --- | --- |
|  |  |  |
| **REAGENT or RESOURCE** | **SOURCE** | **IDENTIFIER** |
| **Antibodies** | | |
| Anti-ATP synthase Immunocapture antibody [12F4AD8AF8] | Abcam | ab109867 |
| ATP5B antibody [6C4] | GeneTex | GTX132925 |
| ATP5A1 antibody [C2C3] | GeneTex | GTX104671 |
| CD63 antibody | GeneTex | GTX135220 |
| CD81 antibody | GeneTex | GTX637264 |
| CD40 antibody | GeneTex | GTX101447 |
| IgG (H+L) Goat anti-Mouse, Alexa Fluor™ 488, Superclonal™ | Invitrogin | PIA28175 |
| Goat Anti-Rabbit IgG H&L (HRP) | Abcam | ab97051 |
| Goat Anti-Mouse IgG H&L (HRP) | Abcam | ab97023 |
| Mouse IgG2b, kappa monoclonal [MPC-11] - Isotype Control | Abcam | ab18457 |
| FYN antibody [N1N3] | GeneTex | GTX101189 |
| Rabbit anti-GAPDH | Bioshop | GAP001R |
|  |  |  |
| **REAGENT or RESOURCE** | | |
| Dulbecco’s modified Eagle’s medium | Gibco | 12800-017 |
| RPMI Medium 1640 | Gibco | 31800-022 |
| Certified Fetal Bovine Serum (FBS) | Biological Industries | 04-001-1A |
| Paraformaldehyde | Sigma-Aldrich | P6148 |
| 4′,6-diamidino-2-phenylindole | Invitrogin | R37108 |
| Sodium deoxycholate | Sigma-Aldrich | D6750 |
| Triethylammonium bicarbonate | Sigma-Aldrich | T7408 |
| Sodium lauroyl sarcosine | Sigma-Aldrich | L9150 |
| Protease Inhibitor Cocktail, Mammalian. (Liquid) | Bioshop | PIC004 |
| Dithiothreitol, Electrophoresis Grade, min 99% | Bioshop | DTT001 |
| S-methyl methanethiosulfonate | Sigma-Aldrich | 208795 |
| Trypsin | ThermoFisher | 90057S |
| Trypan blue solution | Corning | 25-900-CI |
| Formaldehyde-13C, d2 solution | Sigma-Aldrich | 596388 |
| Formaldehyde solution | Sigma-Aldrich | F8775 |
| ammonia | WAKO | 010-03166 |
| formic acid | Sigma-Aldrich | F0507 |
| glutaraldehyde solution | Sigma-Aldrich | G5882 |
| Triton X-100 | Sigma-Aldrich | T8787 |
| 0.45 μm polyvinyl-difluoride (PVDF) membranes . | Millipore | IPVH85R |
| Polyethyleneimine, linear, M.W. 25000 | AlfaAesar | 9002-98-6 |
| puromycin | Selleckchem | 58-58-2 |
| citreoviridin | Cayman Chemical Company | SC-202545A |
| Adenosine 5′-diphosphate | Sigma-Aldrich | 01905 |
| Adenosine 5′-triphosphate disodium salt hydrate | Sigma-Aldrich | A2383 |
| A740003 | MCE | 861393-28-4 |
| mdivi-1 | Sigma-Aldrich | M0199 |
| Amersham™ Protran™ 0.45µm NC | GE healthcare | 10600002 |
| isopropyl-b-D-thiogalactopyranoside | Bioshop | IPT002 |
| guanidine hydrochloride | JT Baker | JT-0510-F9 |
| Dimethyl sulfoxide | Sigma-Aldrich | D1435 |
| Pen-Strep Ampho. Solution | Biological Industries | 03-033-1B |
| ampicillin | Bioshop | 69-52-3 |
| urea | Bioshop | 57-13-6 |
|  |  |  |
| **Critical Commercial Assays** |  |  |
| MitoTracker | Thermo Fisher Scientific | M7512 |
| ATP bioluminescence assay kit | Sigma-Aldrich | FLAA-1KT |
| calcium assay kit | Abcam | ab112115 |
| cDNA Synthesis Kit | Bio-Rad | 170-8891 |
| nucleic acid stain | Seeing Bioscience | E-100 |
| DH5a cells | RBC Bioscience | P1101-20006 |
| Pierce BCA Protein Assay kit | Thermo Fisher Scientific | 23225 |
| ELISA MAX™ Deluxe Set Human IL-2 | Biolegend | 431804 |
| ELISA MAX™ Deluxe Set Human IFN-γ | Biolegend | 430104 |
|  |  |  |
| **Experimental Models: Cell Lines** |  |  |
| A549 | ATCC | CCL-185 |
| SK-N-BE(2)C | ATCC | CRL-2268 |
| IMR-90 | ATCC | CCL-186 |
| Jurkat T-cell | ATCC | TIB-152 |
|  |  |  |
| **Deposited Data** |  |  |
| ProteomeXchange (plasma membrane proteome) | This Manuscript | PXD014995 |
|  |  |  |
| **Oligonucletide** |  |  |
| NM_012062.3-1152s21c1 | RNA Technology Platform and Gene Manlpulation Core, Institute of Molecular Biology, Academia Sinica | TRCN0000318424 |
| NM_012062.3-2085s21c1 |  | TRCN0000318426 |
| Vector : pLKO_TRC005 |  | ASN0000000006 |
|  |  |  |
| **Software and Algorithms** |  |  |
| MaxQuant 2.0.3.0 | MPI Biochemistry | <https://www.maxquant.org/> |
| ICY | bioimageanalysis | <http://icy.bioimageanalysis.org/> |
| GraphPad Prism 8 | GraphPad Software | https://www.graphpad.com/scientific-software/prism |
| GenePix Pro 6.0 | Molecular Devices | <https://support.moleculardevices.com/> |
| PyMOL | Schrödinger | [PyMOL \| pymol.org](https://pymol.org/2/) |
| LigPlot^+^ 2.2.5 | European Molecular Biology Laboratory | [LIGPLOT home page (ebi.ac.uk)](https://www.ebi.ac.uk/thornton-srv/software/LIGPLOT/) |
| Membrain | Shanghai Jiaotong University | <http://www.csbio.sjtu.edu.cn/bioinf/MemBrain/> |
|  |  |  |
| **Other** |  |  |
| ELISA Reader | BioRad | Thermo Multiskan FC |
| ultracentrifugation | Hitachi | CP80WX |
| Confocal laser microscope | Zeiss | LSM780 |
| LTQ-Orbitrap XL | Thermo Electron | LTQ Orbitrap XL |
| nanoACQUITY UPLC | Waters | 176016000 |
| NanoSight | Duxbury | NS300 |
| Transmission electron microscope | Hitachi | H-7650 |
| luminescent image analyzer | ProteinSimple | FluorChem M |
| microarray scanner | Axon GenePix | GenePix 4100A |
