# Supplementary material for: Ectopic ATP synthase stimulates the secretion of extracellular vesicles in cancer cells
Source: Commun Biol. 2023 Jun 15;6:642. doi: 10.1038/s42003-023-05008-5 (PMC10272197; doi:10.1038/s42003-023-05008-5)
Supplement: Supplementary file 10 — Reporting Summary [file 42003_2023_5008_MOESM10_ESM.pdf]

# nature portfolio

Corresponding author(s): Hsueh-Fen Juan

Last updated by author(s): May 10, 2023

# Reporting Summary

Nature Portfolio wishes to improve the reproducibility of the work that we publish. This form provides structure for consistency and transparency in reporting. For further information on Nature Portfolio policies, see our Editorial Policies and the Editorial Policy Checklist.

## Statistics

For all statistical analyses, confirm that the following items are present in the figure legend, table legend, main text, or Methods section.

| n/a | Confirmed | |
|---|---|---|
| ☐ | ☒ | The exact sample size (*n*) for each experimental group/condition, given as a discrete number and unit of measurement |
| ☐ | ☒ | A statement on whether measurements were taken from distinct samples or whether the same sample was measured repeatedly |
| ☐ | ☒ | The statistical test(s) used AND whether they are one- or two-sided<br>*Only common tests should be described solely by name; describe more complex techniques in the Methods section.* |
| ☐ | ☒ | A description of all covariates tested |
| ☐ | ☒ | A description of any assumptions or corrections, such as tests of normality and adjustment for multiple comparisons |
| ☐ | ☒ | A full description of the statistical parameters including central tendency (e.g. means) or other basic estimates (e.g. regression coefficient) AND variation (e.g. standard deviation) or associated estimates of uncertainty (e.g. confidence intervals) |
| ☐ | ☒ | For null hypothesis testing, the test statistic (e.g. *F*, *t*, *r*) with confidence intervals, effect sizes, degrees of freedom and *P* value noted<br>*Give P values as exact values whenever suitable.* |
| ☒ | ☐ | For Bayesian analysis, information on the choice of priors and Markov chain Monte Carlo settings |
| ☒ | ☐ | For hierarchical and complex designs, identification of the appropriate level for tests and full reporting of outcomes |
| ☒ | ☐ | Estimates of effect sizes (e.g. Cohen's *d*, Pearson's *r*), indicating how they were calculated |

*Our web collection on statistics for biologists contains articles on many of the points above.*

## Software and code

Policy information about availability of computer code

| | |
|---|---|
| Data collection | 1. The nanoACQUITY ultra-performance liquid chromatography system (Waters) was used in conjunction with the LTQ-Orbitrap XL mass spectrometer (Thermo Electron) to obtain the mass spectrometry data.<br>2. FluorChem M (ProteinSimple) was utilized to analyze the protein bands.<br>3.Immunofluorescent images were taken using a Zeiss LSM780 confocal microscope (Zeiss).<br>4. Images of extracellular vesicles were visualized with a Hitachi H-7650 transmission electron microscope.<br>5. Size distribution and concentration of extracellular vesicles were analyzed using NanoSight NS300 |
| Data analysis | 1. MaxQuant (version 2.0.3.0) was used to analyze the raw MS spectra data for protein identification.<br>2. ImageJ was performed to analyze the signal of extracellular vesicles.<br>3. Icy (deChaumont et al, 2012) was used to quantify mitochondrial perimeter.<br>4. Zen 2009 software (Zeiss) was used to provide fluorescence images.<br>5. Excel (2019) was used to generate all plots in this manuscript. |

For manuscripts utilizing custom algorithms or software that are central to the research but not yet described in published literature, software must be made available to editors and reviewers. We strongly encourage code deposition in a community repository (e.g. GitHub). See the Nature Portfolio guidelines for submitting code & software for further information.

## Data

Policy information about availability of data

All manuscripts must include a data availability statement. This statement should provide the following information, where applicable:

- Accession codes, unique identifiers, or web links for publicly available datasets
- A description of any restrictions on data availability
- For clinical datasets or third party data, please ensure that the statement adheres to our policy

The original mass spectrometry data has been deposited in the ProteomeXchange Consortium through the PRIDE partner repository, with dataset identifiers PXD014995

## Human research participants

Policy information about studies involving human research participants and Sex and Gender in Research.

| | |
|---|---|
| Reporting on sex and gender | NA |
| Population characteristics | NA |
| Recruitment | NA |
| Ethics oversight | NA |

Note that full information on the approval of the study protocol must also be provided in the manuscript.

# Field-specific reporting

Please select the one below that is the best fit for your research. If you are not sure, read the appropriate sections before making your selection.

- [x] Life sciences
- [ ] Behavioural & social sciences
- [ ] Ecological, evolutionary & environmental sciences

For a reference copy of the document with all sections, see nature.com/documents/nr-reporting-summary-flat.pdf

# Life sciences study design

All studies must disclose on these points even when the disclosure is negative.

| | |
|---|---|
| Sample size | No sample-size calculation was performed. |
| Data exclusions | No data were excluded from the analyses. |
| Replication | In all experiments, three technical replicates were performed for each of the two independent biological replicates. |
| Randomization | Cells were seeded in parallel and randomly allocated to different treatments. |
| Blinding | Blinding was not applied during experiments. |

# Reporting for specific materials, systems and methods

We require information from authors about some types of materials, experimental systems and methods used in many studies. Here, indicate whether each material, system or method listed is relevant to your study. If you are not sure if a list item applies to your research, read the appropriate section before selecting a response.

## Materials & experimental systems

| n/a | Involved in the study |
| --- | --- |
| ☐ | ☒ Antibodies |
| ☐ | ☒ Eukaryotic cell lines |
| ☒ | ☐ Palaeontology and archaeology |
| ☒ | ☐ Animals and other organisms |
| ☒ | ☐ Clinical data |
| ☒ | ☐ Dual use research of concern |

## Methods

| n/a | Involved in the study |
| --- | --- |
| ☒ | ☐ ChIP-seq |
| ☒ | ☐ Flow cytometry |
| ☒ | ☐ MRI-based neuroimaging |

# Antibodies

| | |
| --- | --- |
| Antibodies used | 1. The primary antibodies against the following proteins were used in this study: ATP synthase complex (Immunofluore.cence, Flow cytometry; Abcam ab109867), ATP5B (Western blotting, Immunofluorescence; GeneTex GTX132925), ATP5A1 (Western blotting; GeneTex GTX104671), CD63 (Western blotting, Immunofluorescence; GeneTex GTX135220), CD81 (Western blotting, Immunofluorescence; GeneTex GTX637264), CD40 (Western blotting, Immunofluorescence; GeneTex GTX101447), FYN (Western blotting, Immunofluorescence; GeneTex GTX101189), GAPDH (Western blotting; Bioshop GAP001R).<br>2. The secondary antibodies used in this study are listed as follows: Goat Anti-Mouse IgG H&L (HRP) (Western blotting; Abcam ab97023), Goat Anti-Rabbit IgG H&L (HRP) (Western blotting; Abcam ab97051), Alexa488-conjugated Goat anti-mouse IgG (Immunofluorescence; Invitrogen Cat#A11001), Alexa488-conjugated Goat anti-rabbit IgG (Immunofluorescence; Invitrogen Cat#A11008). |
| Validation | 1. Anti-ATP synthase Immunocapture antibody (Abcam ab109867) antibody reacts with human species and is suitable for Flow Cyt. This antibody was validated using immunofluorescence and dot blot in this study.<br>2. ATP5B (GeneTex GTX132925), ATP5A1 (GeneTex GTX104671) react with human species and are suitable for WB. These antibody were validated using Western blotting, immunofluorescence and dot blot in this study.<br>3. CD63 (GeneTex GTX135220), CD81 (GeneTex GTX637264), CD40 (GeneTex GTX101447) react with human species and are suitable for WB. These antibody were validated using western blotting in this study.<br>4. FYN (GeneTex GTX101189) reacts with human species and is suitable for WB, ICC/IF, IHC-Wm. This antibody was validated using immunofluorescence in this study.<br>5. GAPDH (Bioshop GAP001R) reacts with human species and is suitable for WB. This antibody was validated using Western blotting in this study. |

# Eukaryotic cell lines

Policy information about cell lines and Sex and Gender in Research

| | |
| --- | --- |
| Cell line source(s) | Human lung cancer cell line A549 (ATCC Cat# CCL-185), human neuroblastoma cell line SK-N-BE(2)C (ATCC Cat# CRL-2268, RRID:CVCL_0529) and human breast cancer cell line T47D (ATCC Cat# CRL-3437) were purchased from the American Type Tissue Collection (ATCC). Human T lymphcyte cell line Jurkat T cell (ATCC Cat# TIB-152) were gifted from Dr. Hao-Sen Chiang, Department of Life Science, National Taiwan University, Taiwan. |
| Authentication | All cell lines were authenticated by matching the STR profile to the ATCC public STR Database. |
| Mycoplasma contamination | All cell lines used in this study were negative for mycoplasma contamination. |
| Commonly misidentified lines (See ICLAC register) | No commonly misidentified cell lines were used. |
